# An intricate balance of hydrogen bonding, ion atmosphere and dynamics facilitates a seamless uracil to cytosine substitution in the U-turn of the neomycin-sensing riboswitch

**DOI:** 10.1093/nar/gky490

**Published:** 2018-06-09

**Authors:** Miroslav Krepl, Jennifer Vögele, Holger Kruse, Elke Duchardt-Ferner, Jens Wöhnert, Jiri Sponer

**Affiliations:** 1 Institute of Biophysics of the Czech Academy of Sciences, Kralovopolska 135, 612 65 Brno, Czech Republic; 2 Institute of Molecular Biosciences and Center for Biomolecular Magnetic Resonance (BMRZ), Goethe-University Frankfurt, Max-von-Laue-Str. 9, 60438 Frankfurt, Germany; 3 Regional Centre of Advanced Technologies and Materials, Department of Physical Chemistry, Faculty of Science, Palacky University Olomouc, 17. listopadu 12, 771 46 Olomouc, Czech Republic

## Abstract

The neomycin sensing riboswitch is the smallest biologically functional RNA riboswitch, forming a hairpin capped with a U-turn loop—a well-known RNA motif containing a conserved uracil. It was shown previously that a U→C substitution of the eponymous conserved uracil does not alter the riboswitch structure due to C protonation at N3. Furthermore, cytosine is evolutionary permitted to replace uracil in other U-turns. Here, we use molecular dynamics simulations to study the molecular basis of this substitution in the neomycin sensing riboswitch and show that a structure-stabilizing monovalent cation-binding site in the wild-type RNA is the main reason for its negligible structural effect. We then use NMR spectroscopy to confirm the existence of this cation-binding site and to demonstrate its effects on RNA stability. Lastly, using quantum chemical calculations, we show that the cation-binding site is altering the electronic environment of the wild-type U-turn so that it is more similar to the cytosine mutant. The study reveals an amazingly complex and delicate interplay between various energy contributions shaping up the 3D structure and evolution of nucleic acids.

## INTRODUCTION

**Figure 1.**
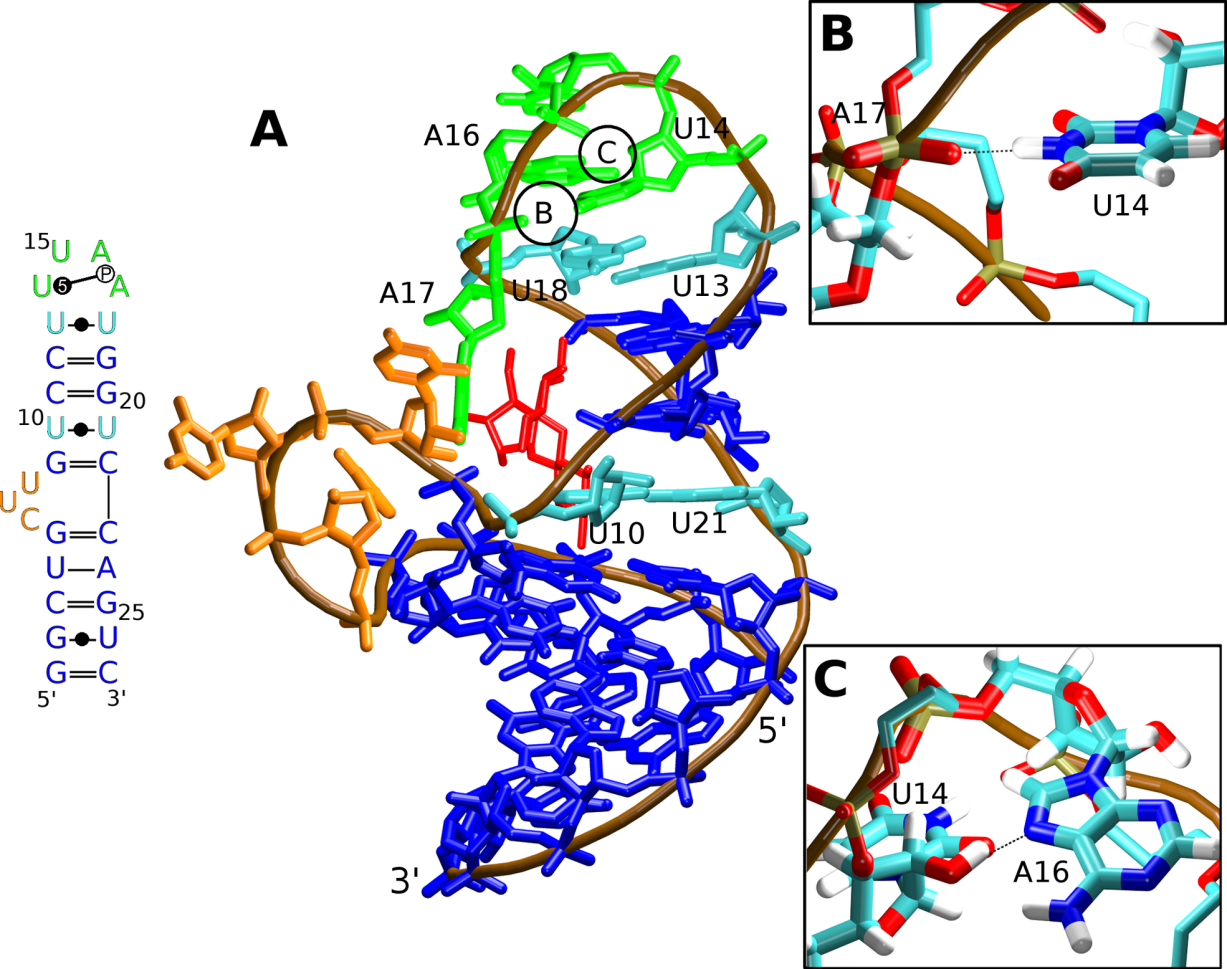
(**A**) The secondary structure (left; standard Leontis & Westhof RNA base pair annotation extended by the classification of base-phosphate interactions is used, see also http://ndbserver.rutgers.edu/ndbmodule/ndb-help.html) (6,7) and the 3D representation (right) of the neomycin sensing riboswitch (PDB: 2n0j). The canonical and GU wobble base pairs are in blue, the *c*WW U/U base pairs are in cyan, the bulge is in orange, and the U-turn loop is in green. The RNA backbone is traced in brown and the bound ribostamycin ligand is shown in red. The chain termini and selected nucleotides are labeled. The circles B and C mark positions of the U14(N3)/A17(OP2) and U14(O2′)/A16(N7) signature H-bonds (the dashed black lines) with their structural details shown in the inset figures (**B**) and (**C**), respectively.

Riboswitches are RNA structural elements commonly found in the 5′-untranslated regions of mRNAs. They are able to alter their structure in response to ligand binding and can thereby regulate RNA expression. One example for such a riboswitch is the synthetic neomycin sensing riboswitch (NSR) (1). It was developed by combined *in-vitro* and *in-vivo* selection to regulate translation initiation in *Saccharomyces cerevisiae* in response to the aminoglycoside antibiotics neomycin or ribostamycin. Containing only 27 nucleotides, it is the smallest known biologically functional riboswitch. In presence of the ligand, it assumes a very stable hairpin-like structure (Figure 1A) that impedes the scanning of the mRNA by the small ribosomal subunit and thus prevents its translation (2). While the hairpin folds even without the ligand, it is significantly less stable in the absence of the ligand, allowing the small ribosomal subunit to read through it and reach the start codon (2).

The apex of the NSR hairpin folds into a U-turn—a well-known RNA submotif widespread in diverse RNA structures (3,4). U-turns facilitate a sharp bend of the sugar-phosphate backbone and are thus one of the structural elements that can cap a hairpin loop (Figure 1A). Its consensus sequence contains a 5′-UNR-3′ nucleotide triplet, where N is any nucleotide and R is a purine. In addition to stacking interactions between the bases, the canonical U-turn possesses two signature H-bonds. One hydrogen bond is formed between the N3 atom of the conserved uracil and the non-bridging oxygen of the phosphate group downstream of the purine nucleotide (Figure 1B). The second hydrogen bond involves the 2′-hydroxyl group of the conserved uridine and the N7 atom of the purine base (Figure 1C). In addition, the U-turn loop is commonly closed by a non-canonical reverse Hoogsteen A/U, sheared G/A, or Y/Y base pair where Y is a pyrimidine (3). In the case of the NSR, there is a *c*WW U/U (5,6) closing base pair and an additional adenine is inserted at the 3′-end of the U-turn. Thus, the NSR hairpin is capped by a hexaloop with a 5′-U_13_U_14_U_15_A_16_A_17_U_18_-3′ sequence. Its key structural elements are the *c*WW U13/U18 base pair, the 5BPh U14(N3)/A17(OP2) base-phosphate interaction (7), and the U14(O2′)/A16(N7) H-bond. The remainder of the NSR hairpin is composed of canonical base pairs except the *c*WW U10/U21 base pair and a three-nucleotide bulge formed by the C6, U7 and U8 nucleotides (Figure 1A).

While the 3D structure of the U-turn motif is strongly conserved in evolution, in the NSR the conserved uracil in its 5′-UNR-3′ U-turn consensus sequence can be replaced by cytosine without altering the structure of the motif or disrupting the function of the NSR riboswitch (8). The U→C substitution in U-turns has also been observed in other biologically relevant systems (8). At first sight, the conservation of the structure is puzzling as the N3 atom in cytosine is normally not protonated at physiological pH and thus cannot form the critical H-bond interaction between the N3 atom and the downstream phosphate group (Figure 1B). However, NMR experiments for the NSR showed that the cytosine substituting uracil in the U-turn becomes N3-protonated and can therefore form a perfectly isosteric substitution for the uracil, enabling the formation of the critical H-bond to the backbone phosphate group (8). Remarkably, the cytosine remains protonated even at pH values as high as 8.3, suggesting that in this particular environment its p*K*_a_ must be shifted by more than 4 pH units compared to cytosine residues in unfolded RNAs where the p*K*_a_ of the cytidine N3 atom is ∼4.2 (9). Thus, the NSR U14C+ mutant is one of the very few reported RNA systems containing protonated nucleotides with pK_a_'s shifted significantly above neutrality (10–12). Furthermore, NMR measurements of *trans*-hydrogen bond N–P and H–P scalar couplings show that these couplings are slightly larger in the U14C+ mutant than in the wild-type RNA suggesting a shorter and thus a slightly stronger base-phosphate hydrogen bonding interaction in the mutant (8,13).

Classical atomistic molecular dynamics (MD) simulations are a computational method for studying structural dynamics of molecules using a carefully calibrated set of molecular mechanics (MM) models—the force fields (14). MD simulations have already been successfully applied to various RNA systems in the past, including the U-turn motif (15–20). Compared to experimental methods of structure determination, one main advantage of MD is the ability to observe the movement of atoms with an almost unlimited temporal and spatial resolution. Practical limitations are simulation length and the accuracy of the force fields (14).

For a description of the potential energy surfaces, quantum chemical (or quantum mechanical, QM) calculations are physically more accurate than MM. QM methods are indispensable for refinements of MM force-field parameters (21). Unfortunately, QM calculations are computationally expensive and do not allow extensive sampling of the conformational space of larger biomolecular systems. They are typically used for small model systems although large-scale QM calculations have been for example applied to riboswitches (22,23). The MM calculations can be combined with QM in a multi-scale approach into so-called QM/MM calculations (24–31). By describing only a small part of the whole system with quantum chemistry (the ‘QM region/zone’), it is possible to study electronic-structure effects that cannot be described by pure MM methods, while still partially retaining the ability to calculate larger systems.

In this work, we study the NSR by a combination of explicit-solvent MD simulations, QM and QM/MM calculations, as well as NMR experiments. In our calculations, we use the experimentally determined structure of the NSR in complex with ribostamycin (RIO) (PDB: 2n0j) (32). The structure was obtained by high-resolution solution NMR spectroscopy. Except for the three-nucleotide bulge (Figure 1A) which is dynamically disordered according to the NMR-data, the positions of all nucleotides and of the RIO ligand were clearly determined by a large number of NMR-restraints such as NOEs. The structure is thus useful for theoretical study as there is large amount of primary experimental data that can be used to evaluate the performance of the computational methods.

The main goal of our study was to provide an atomistic description of the structural and dynamic consequences of the isosteric uracil → cytosine substitution in the U-turn and to describe the specific dynamics of the wild-type and of the mutant NSR. To that end, we use the wild-type NSR structure to prepare the U14C+ mutant and conduct MD simulations. In the past, MD simulations were shown to be an efficient tool to study the structural response of RNA molecules to different base protonation states, as exemplified by studies of catalytic RNA molecules (33–35). However, so far very few MD simulation studies dealt with an isosteric substitution of a non-protonated for a protonated nucleotide (36,37). The simulations presented here are very effective in describing the NSR’s internal dynamics and solute–solvent interactions; data typically unobservable in NMR experiments (38,39). However, we also show that the various approximations used in the MM force field cannot fully describe all effects of the U14C+ mutation, especially those related to subtle changes in the electronic environment of the RNA. Thus, we supplement our MD simulations with QM and QM/MM calculations, which inherently account for these effects. The combined use of different computational methods ensures that all the molecular aspects of the U14C+ mutation are properly addressed. The computations show that the uracil → cytosine substitution in the U-turn is associated with an intricate shift in the balance among diverse energy terms, unmasking the complexity of the physical chemistry of molecular interactions in nucleic acids.

## MATERIALS AND METHODS

### MD simulations

We have used the first frame of the NMR ensemble (PDB: 2n0j) (32) as the starting structure of all simulations. See Supplementary Data for general comments about the utilization of the NMR structures in MD simulations. The structure of the NSR containing the U14C+ mutation was prepared by molecular modeling. NSR structures without the ribostamycin ligand and the structure of the free ribostamycin were prepared by deleting the respective molecules from the starting structure. The topology and coordinate files were generated by the tleap module of AMBER 16 (40). We have used the bsc0χ_OL3_ force field (21,41,42) for RNA and the GAFF force field (43) for the RIO ligand. The partial charges for RIO were derived by antechamber using the AM1-BCC method (44,45) and the parameters for protonated cytidine (C14+) were taken from Ref. (36). Both RIO and protonated cytidine parameters are available in the Supplementary Data. The systems were solvated in an octahedral box of SPC/E waters (46) with a minimal distance of 11 Å between the solute and the box boundary. Either KCl or NaCl ions (47) were used to neutralize the system, achieving an overall excess salt concentration of ∼0.15 M. The simulation equilibration and production protocol is described in detail in the Supplementary Data.

### QM/MM optimizations

Non-periodic QM/MM optimizations using an additive scheme with electrostatic (point-charge) embedding were performed using an in-house modified version of the QM/MM module (48) within the sander program of AMBER 14 to enable calculations with Turbomole (49). We have used a manually selected snapshot from MD simulations of each system (wild-type and mutant) as the initial structures for the QM/MM optimizations. The QM region was centered around the U14(N3)/A17(OP2) and C14+(N3)/A17(OP2) interactions, respectively. It contained 285 atoms for the system with the U14 base, 317 atoms for the system with the U14 base and one specific K^+^ ion included (see below), and 255 atoms for the system with the C14+ base. Note that to simplify the QM region definition the structures without the RIO ligand were used for QM/MM calculations. The QM region was manually selected to contain the area of interest along with an additional layer of RNA atoms as buffer zone between the area of interest and MM parts of the system. The boundary region was handled by cutting only through C-C single bonds, using the default settings for the H link-atom scheme as described in Ref. (48). The QM region within the context of the full NSR system is shown in the Supplementary Figure S1. The QM/MM systems were placed in the center of a sphere of SPC/E waters with a radius of 34 Å. Positions of the water molecules were equilibrated prior to the QM/MM calculations. For the QM region calculations we employed the TPSS-D3 dispersion-corrected density functional theory (DFT) treatment with an all-electron atomic-orbital def2-TZVP basis set for all elements (50,51), a well-tested approach for QM calculations of nucleic acids (52). All DFT-D3 calculations employed Becke-Johnson damping (53,54).

### Analyses

The calculations were analyzed using the cpptraj module (55) of AMBER 16. The VMD (56) program was used for visualization, and graphs and figures were produced by LibreOffice and Raster3D (57), respectively.

The primary way of assessing the simulation stability was evaluation of the average distance violations of the experimental NOE (Nuclear Overhauser effect) ensemble-averaged distances. We have calculated a weighted average of the NOE distances in the simulation ensemble and compared this value with the upper-bound experimental NOE distances. A complete list of the simulation NOE violations for each system along with a commentary is available in the Supplementary Data. The H-bond interactions were analyzed by monitoring the distances and angles between the relevant heavy atoms. H-bonds were counted when the heavy-atom distance was <3.5 Å and the donor-hydrogen-acceptor angle was >120°. In addition, the simulation structural stability was extensively monitored by visual analysis of the trajectories. The cation-binding sites in simulations were evaluated by computing the K^+^ (or Na^+^) ion density grid and by detailed analysis of the interactions between the cations and individual RNA atoms. All predicted ion sites were visually inspected.

### NMR spectroscopy

Wild-type and C14+ NSR variants were prepared by *in vitro* transcription using commercially available unlabeled nucleotide triphosphates (SigmaAldrich) and folded into monomeric hairpin forms as described in detail previously (2,32). For the wild-type RNA, a selectively ^13^C,^15^N-U-labeled sample was prepared using commercially available ^13^C,^15^N-UTP (Silantes). Ribostamycin (SigmaAldrich) was added in slight excess to the NMR samples and 1:1 complex formation was confirmed by 1D-^1^H-NMR-spectra. All spectra were recorded on Bruker Avance III 600, 700 and 800 MHz NMR-spectrometers equipped with cryogenic ^1^H,^13^C,^15^N or ^1^H,^13^C,^31^P triple resonance probes. For recording of the directly detected ^13^C-1D-spectrum with ^15^N-decoupling a probe with the ^13^C-channel on the inner coil was used to improve the signal-to-noise ratio. All NMR spectra where imino proton resonances were detected were recorded at 10°C unless noted otherwise. The ^13^C-1D-spectra were recorded at 25°C. Spectra were processed with Topspin 3.1 (Bruker). Either 25 mM potassium phosphate (pH 6.5), 50 mM KCl or 50 mM BisTris (pH 6.3) were used as buffer for the different experiments. For the constant time 1D-^1^H-experiments with or without ^31^P-decoupling for measuring the ^2h^J_H,P_ cross-hydrogen bond scalar couplings (58), seven different constant-time delays were employed for both the cross and the reference experiment and all measurements were repeated twice. The signal attenuations as a function of the constant time delay (τ_m_) were then globally fitted to the equation *I*_cross_/*I*_ref_ = cos(π^2h^J_H,P_τ_m_) using Origin. All signal assignments were derived using standard procedures as described previously (2). Note that the uridine C4 and C2 assignments were derived from an 2D-H(N)CO-experiment and are therefore limited to those uridine residues with an observable imino proton resonance.

## RESULTS

### Comparison with primary NMR data reveals satisfactory overall performance of the force field with a localized inaccuracy in the U-turn region

The NSR structure can be divided into four segments—two A-RNA double helices, a three-nucleotide bulge and the U-turn loop. The upper A-RNA segment contains two *c*WW U/U base pairs (Figure 1). All segments were well reproduced in the simulations (Table 1) with only a few NOE distance violations (Table 2, Supplementary Figure S2). This indicates good performance of the force field, at least for standard simulations starting from the experimental structure.

**Table 1.** List of simulations

Simulated system^a^	Number of simulations × length [μs]
2n0j_wt	1 × 10 μs^b^, 5 × 1 μs
2n0j_RIO_wt	1 × 10 μs^b^, 3 × 1 μs
2n0j_RIO_wt_CaseP_OPC^c^	1 × 1 μs
2n0j_RIO_wt_HBfix_A^d^	1 × 1 μs
2n0j_RIO_wt_HBfix_B^d^	3 × 1 μs
2n0j_RIO_wt_HBfix_C^d^	1 × 1 μs
2n0j_C14+	1 × 10 μs^b^, 5 × 1 μs
2n0j_RIO_C14+	1 × 10 μs^b^, 3 × 1 μs
2n0j_RIO_wt_Na^e^	4 × 1 μs
free_RIO^f^	1 × 1 μs
1bvj^g^	1 × 1 μs
3rg5^g^	1 × 0.5 μs

^a^The abbreviations ‘wt’, ‘C14+’, and ‘RIO’ in the simulation names refer to systems containing the wild-type uracil 14, protonated cytosine 14, and the ribostamycin ligand, respectively.

^b^Only the first microsecond of the ten-microsecond simulations, along with all the one-microsecond simulations, is included in the analyses presented in the main text. The rest of the ten-microsecond simulations (i.e. time interval of 1–10 μs) is described in the Supplementary Data.

^c^The Case *et al*. parameters for RNA phosphates (59) in combination with OPC waters (60) were used.

^d^The U14(O2′)/A16(N7) H-bond interaction was stabilized using 1 kcal/mol (variant A) or 2 kcal/mol (variant B) HBfix potential function (61). In variant C, we used 1 kcal/mol repulsive HBfix potential to destabilize the spurious U14(O2′)/U15(O5′) interaction, in addition to the HBfix potential used in variant A. All HBfix potentials were applied in the 2–3 Å hydrogen-acceptor distance range (61).

^e^Na^+^ ions were used instead of K^+^.

^f^Simulation of a free ribostamycin.

^g^Control simulations of other U-turn-containing RNA molecules—HIV-1 RNA A-Rich Hairpin Loop (PDB: 1bvj) (62) and a fragment (residues 47–47K) of the Mouse tRNA(Sec) (PDB: 3rg5) (63).

**Table 2.** Average number of violated NOE distances (larger than 0.3 Å) in MD simulations of the NSR^a^

System^b^	#^c,d^
2n0j_wt	9 of 714
2n0j_RIO_wt	8 of 894
2n0j_RIO_wt_CaseP_OPC	10 of 894
2n0j_RIO_wt_HBfix_A	9 of 894
2n0j_RIO_wt_HBfix_B	6 of 894
2n0j_RIO_wt_HBfix_C	8 of 894
2n0j_RIO_wt_Na	9 of 894
2n0j_C14+	9 of 714
2n0j_RIO_C14+	8 of 894

^a^The list and visualizations of the specific violated NOE distances for the individual simulation ensembles are reported in the Supplementary Data.

^b^For systems where multiple parallel simulations were conducted (see Table 1), the NOE violations were computed for the combined one-microsecond simulation ensembles.

^c^The second number stated is the total number of the measured experimental NOE distances applicable to the specified system.

^d^Note that up to six NOE distance violations were typically related to the U14(HO2′) atom and the unsatisfactory simulation description of the U14(O2′)/A16(N7) U-turn signature interaction (see Supplementary Data).

The only notable inaccuracy of the force-field description was identified in the U-turn motif where the U14(O2′)/A16(N7) signature interaction was sometimes replaced by the U14(O2′)/U15(O5′) interaction (Supplementary Figure S3). This appears to be incorrect as there is a strong experimental evidence for the presence of the U14(O2′)/A16(N7) hydrogen bond in the NSR structure (Supplementary Figure S4) (2,32). Fortunately, the rearrangement to the spurious U14(O2′)/U15(O5′) interaction was reversible on the simulation timescale. It also remained entirely localized and did not lead to additional structural perturbations of the U-turn. Therefore, it did not affect the other results described below except introducing a few localized NOE violations for distances associated with the U14(HO2′) atom (Supplementary Figure S2). Further details about the U14(O2′)/A16(N7) signature interaction and about the efforts to fix its behavior in simulations are described in the Supplementary Data.

### NSR structure and dynamics is strongly influenced by the RIO ligand

**Figure 2.**
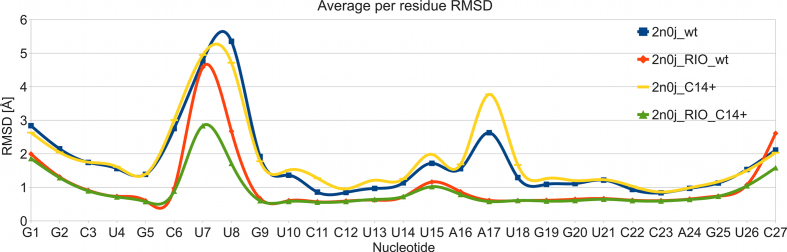
Average heavy atom per residue RMSD in the MD simulations of the individual NSR systems. An averaged structure of each MD trajectory ensemble was used as the reference structure.

We observed universally larger atomic fluctuations of the NSR system in our simulations without the RIO ligand (Figure 2). This was an expected result as according to the experiments the RIO ligand significantly stabilizes the structure of this riboswitch (32). In general, the largest RNA fluctuations were observed for the bulge (nts 6–8) and the U-turn loop (nts 14–17) (Figure 2). Further details of the RIO ligand behavior in MD simulations and its effects on RNA dynamics are described in the Supplementary Data.

### The wild-type (U14) and mutant (C14+) systems show minor difference in U13/U18 base pairing

**Figure 3.**
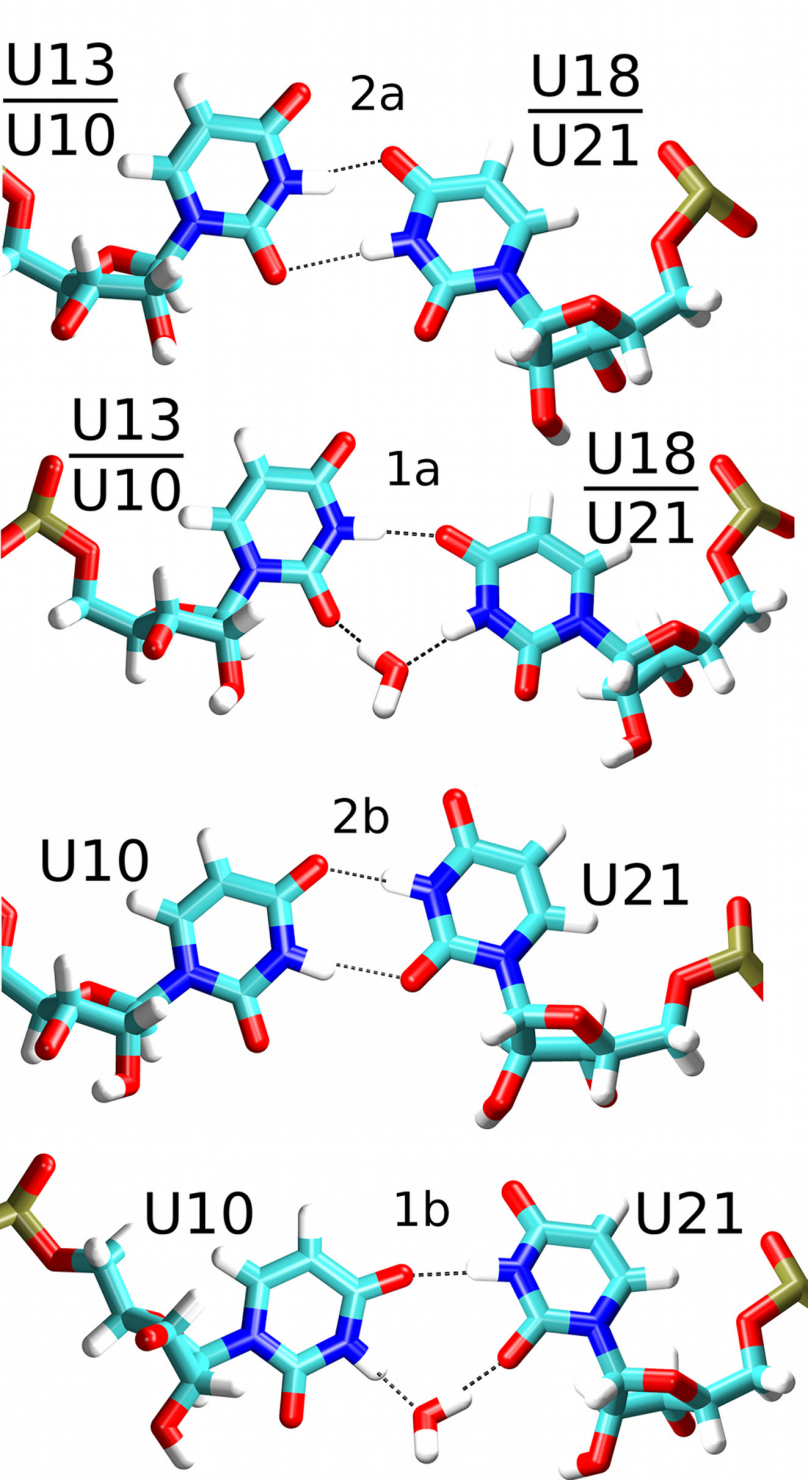
Observed conformations of the U/U base pairs. In simulations, the *c*WW U13/U18 base pair possessed either two direct H-bonds (top, conformation 2a) or one direct and one water-mediated H-bond (top middle, conformation 1a). Both possible symmetry variants of the *c*WW U10/U21 base pair (conformations 2a and 2b, and their water-mediated variants 1a and 1b) were observed in simulations of the NSR in the absence of RIO. In the presence of RIO only conformations 2a and 1a were observed. The dashed black lines indicate H-bonds.

The NSR system with a protonated cytosine 14 (henceforth referred to as ‘C14+’ or ‘mutant’ system) behaved nearly identically to the wild-type (also referred to as ‘U14’ system) in MD simulations, both in terms of NOE violations (Table 2 and Supplementary Figure S2) and structural dynamics (Figure 2). This observation is in full agreement with the previous NMR experiments (8). However, we have observed a slightly different behavior of the U13/U18 base pair which closes the U-turn loop (Figure 1A). A *c*WW U/U base pair with direct U13(N3)/U18(O4) and U18(N3)/U13(O2) H-bonds (conformation 2a; see Figure 3 and Table 3) dominated the simulation ensemble in the presence of RIO for both systems. However, there was also a minor population of a base pair variant where the interaction between U18(N3) and U13(O2) atoms became temporarily water-mediated (conformation 1a; see Figure 3); it was slightly more populated in the C14+ system (Table 3). While NMR experiments did not indicate an altered behavior for the U13/U18 base pair in the C14+ mutant in the presence of RIO (8), the difference observed in simulations with RIO is most likely beyond the detection limit of NMR. The water-mediated variant became the dominant arrangement in the C14+ system without RIO ligand (Table 3). In agreement with the NMR data, the alternative *c*WW U/U base pair arrangements with U13(N3)/U18(O2) and U18(N3)/U13(O4) H-bonds or with a direct U18(N3)/U13(O4) H-bond and a water-mediated U13(N3)/U18(O2) interaction were never sampled in the simulations.

**Table 3.** Populations of the U13/U18 and U10/U21 H-bonding arrangements and the median distances between the A16(OP2) and U18(O4′) atoms and planes of bases 14 and 16, respectively, in MD simulations of the NSR

	U13/U18		U10/U21^a^					
Base pair variant/simulation^b^	2a	1a	2a	1a	2b	1b	A16(OP2)/Base14 anion-π	U18(O4′)/A16 lone pair-π
**NMR^c^**	100%	0%	100%	0%	0%	0%	2.93 ± 0.07 Å	3.01 ± 0.04 Å
**2n0j_wt**	87%	13%	46%	11%	38%	5%	3.22 ± 0.25 Å	3.10 ± 0.13 Å
**2n0j_RIO_wt**	96%	4%	60%	40%	0%	0%	3.22 ± 0.22 Å	3.00 ± 0.09 Å
**2n0j_RIO_wt_CaseP_OPC**	97%	3%	66%	34%	0%	0%	3.22 ± 0.16 Å	3.06 ± 0.09 Å
**2n0j_RIO_wt_HBfix_A**	99%	1%	79%	21%	0%	0%	3.14 ± 0.17 Å	3.02 ± 0.09 Å
**2n0j_RIO_wt_HBfix_B**	98%	2%	62%	38%	0%	0%	3.10 ± 0.14 Å	3.00 ± 0.09 Å
**2n0j_RIO_wt_HBfix_C**	99%	1%	69%	31%	0%	0%	3.10 ± 0.14 Å	3.01 ± 0.09 Å
**2n0j_RIO_wt_Na**	96%	4%	60%	40%	0%	0%	3.29 ± 0.26 Å	3.00 ± 0.09 Å
**2n0j_C14+**	27%	73%	43%	10%	38%	9%	2.95 ± 0.11 Å	3.29 ± 0.19 Å
**2n0j_RIO_C14+**	84%	16%	60%	40%	0%	0%	2.96 ± 0.11 Å	3.00 ± 0.09 Å

^a^There are two possible *c*WW base pair arrangements for base pair U10/U21 (see Figure 3 and Ref. (6)). In arrangement ‘2a’, there are U21(N3)/U10(O2) and U10(N3)/U21(O4) H-bonds while in arrangement ‘2b’, there are U10(N3)/U21(O2) and U21(N3)/U10(O4) H-bonds. In case of the single H-bond variants (indicated as arrangements ‘1a/1b’), the H-bond involving the O2 atom is water-mediated (Figure 3). Note that due to steric hindrance, only one *c*WW base pair arrangement and one water-mediated variant was observed for the U13/U18 base pair (Figure 3).

^b^For systems where multiple parallel simulations were conducted (see Table 1), the analyses were computed for the combined one-microsecond simulation ensembles.

^c^The ‘NMR’ dataset refers to the experimental NMR ensemble of the wild-type NSR in complex with RIO (PDB: 2n0j) (32).

A *c*WW U/U base pairing was observed also for the NSR’s other U/U base pair (U10/U21; see Figure 1). Its base pairing arrangements were not influenced by the C14+ mutation; rather, they depended solely on the presence of the RIO ligand. In the simulations without the ligand, both isosterically possible (6) *c*WW two H-bond arrangements of the U10/U21 base pair were observed (conformations 2a and 2b; see Figure 3 and Table 3), owing to a larger conformational freedom of the NSR system without the ligand (Figure 2). Consequently, also two different alternative arrangements with one direct H-bond and one water-mediated interaction could be observed in the ligand-free state (conformations 1a and 1b; see Figure 3 and Table 3). In contrast, only one of the two possible arrangements with two direct H-bonds (conformation 2a) and one of the two possible arrangements with one direct H-bond (conformation 1a) for the U10/U21 base pair were observed in simulations with RIO (Table 3), a consequence of conformational restrictions imposed by the bound ligand (Figure 2).

The NMR structures of the NSR/RIO complex showed a *c*WW U10/U21 base pair with a direct hydrogen bond between the U10 imino proton and U21(O4) and revealed a C1′–C1′ atom distance for the U10/U21 base pair intermediary to distances typical for the two H-bond and one H-bond arrangements of U/U base pairs (2). Furthermore, the U21 imino proton resonance in both the wild-type and the C14+ complex is strongly broadened and shifted upfield to ∼9.2 ppm (2,8). This experimental observation could be rationalized by an equilibrium between the 2a and 1a conformations of the U10/U21 base pair; see Figure 3 and Table 3. This interpretation is strongly supported by MD simulations of the NSR/RIO complex, showing a rapid exchange between the 2a (population ∼60%) and 1a (∼40%) conformations (Table 3) for the U10/U21 base pair.

### The U14C+ mutation alters the anion-π interaction of the Y14 base with the A16 phosphate

**Figure 4.**
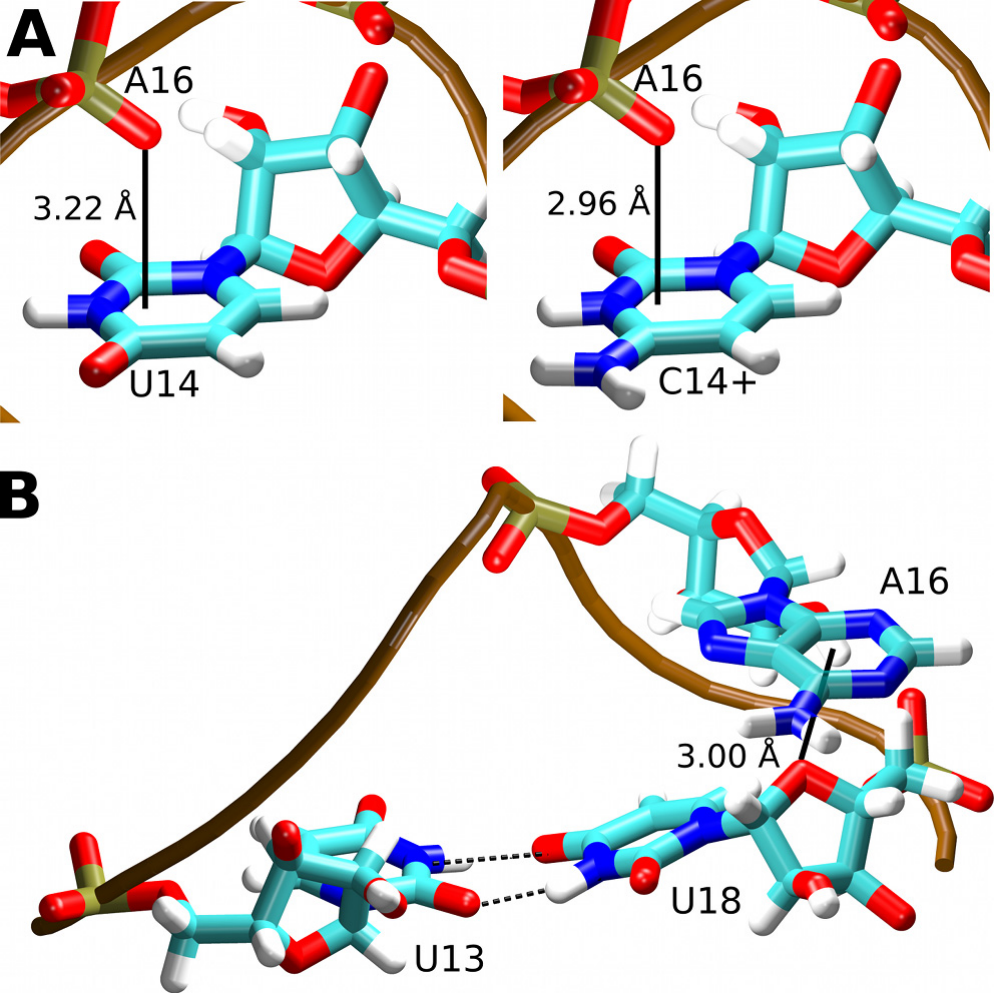
(**A**) The anion-π interactions (solid black lines) of the A16(OP2) atom and the U14 (left) and C14+ (right) bases. (**B**) Lone pair–π interaction (solid black line) between the U18(O4′) atom and the A16 base. The median distances observed in the MD simulations between the planes of the bases and the oxygen atoms are labeled.

An anion-π interaction between a backbone phosphate group non-bridging oxygen and the base of the conserved uracil was earlier suggested to play a role in the stabilization of the U-turn motif (64). Therefore, we have monitored the distance between the U14/C14+ bases and the OP2 oxygen of the A16 nucleotide (Figure 4A) in our MD simulations. The median distance between the plane of the base and the OP2 atom was 3.22 Å in the U14 simulations and 2.96 and 2.95 Å in the C14+ simulations with and without RIO, respectively (Table 3). There was no concomitant change in the RNA backbone dihedrals (not shown). The change in the distance between the A16 phosphate group and the base plane of nucleotide 14, as observed in MD simulations, could explain the significant chemical shift difference observed for the A16 phosphate group resonance in 1D-^31^P-NMR spectra between the wild-type and the C14+ NSR/RIO complexes (Supplementary Figure S5).

The NSR structure contains an additional oxygen lone pair-π interaction (65,66) between the U18(O4′) atom and the A16 base (Figure 4B). This interaction is not a general feature of the U-turn motif as its presence in NSR is due to a system-specific insertion of the A17 nucleotide into the canonical sequence of the U-turn. This interaction was fully maintained in MD simulations of both the wild-type and the mutant RNA, with the median distance between the plane of the A16 base and the O4′ atom being nearly identical to the NMR ensemble (Table 3). The distance was slightly increased in the simulations of the NSR without the RIO ligand. This can be attributed to the increased fluctuations of the NSR without the ligand (Figure 2) and the larger prevalence of the 1a conformation of the U13/U18 base pair in those simulations (Figure 3 and Table 3) as the change from the 2a to the 1a conformation is associated with a small positional shift of the U18 nucleotide.

Lastly, note that the force field used in our MD simulations does not include any π-orbital parameterization. It has nevertheless been shown in the past that stacking interactions of nucleic acid bases can be realistically described using the AMBER-type of force field, without any specific π-terms (67). The base stacking, despite being often referred to as π–π interaction, is in fact a common Van der Waals interaction without any specific role of the π orbitals (67). Similarly, although the nucleobase—phosphate oxygen interaction has been called an anion–π interaction, it does not necessarily mean that there is some significant molecular-orbital interplay between the two interacting partners. In fact, Wheeler and Bloom showed that similar interactions can be understood as straightforward charge–dipole electrostatic interactions (68) which are thus well describable by the simulation force field, as demonstrated by our simulations of the NSR.

### Adaptation of the U-turn cation atmosphere helps to accommodate the U14C+ mutation

**Figure 5.**
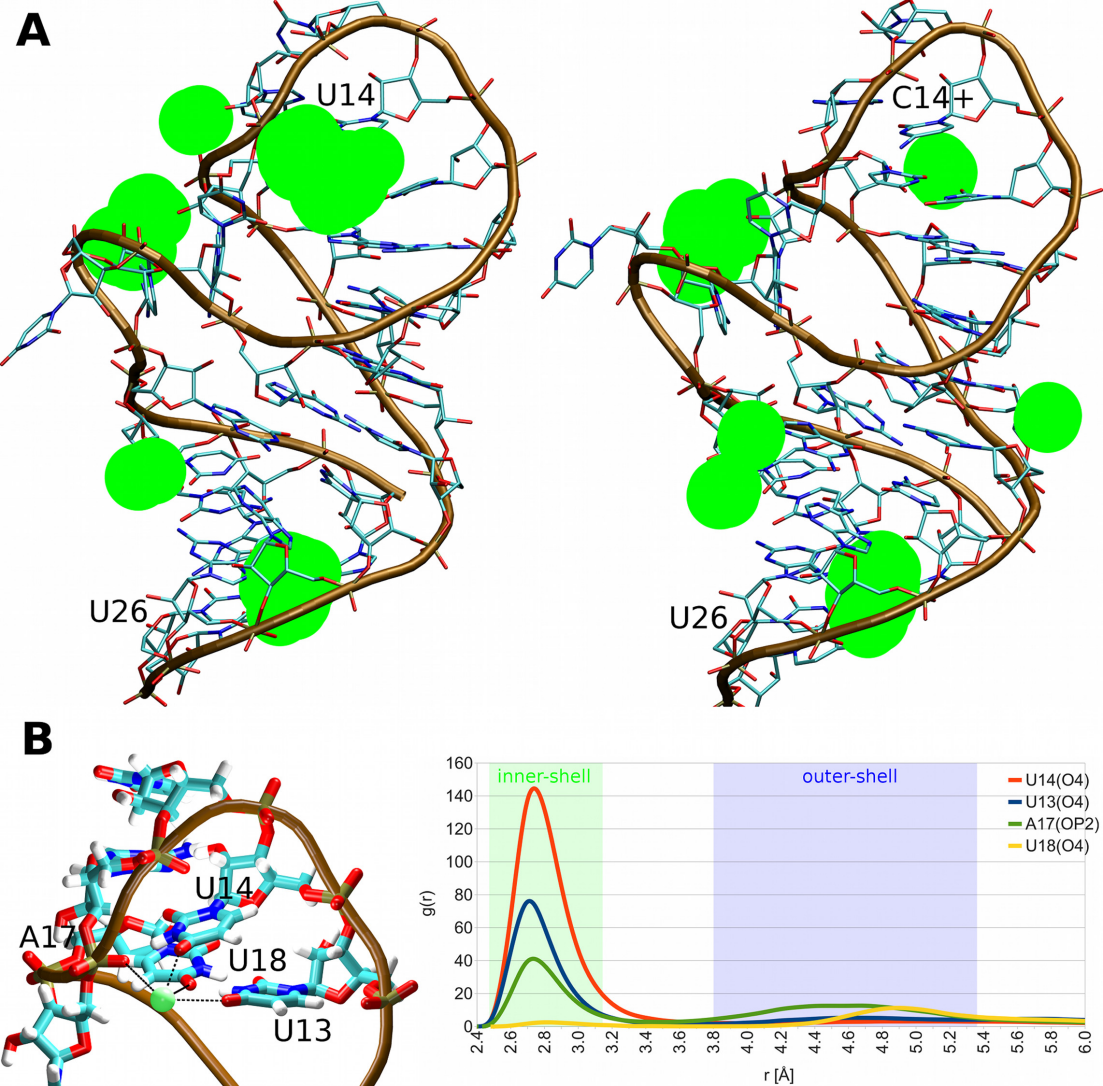
(**A**) Potassium ion binding to the NSR as seen in simulations. The green spheres represent the five highest density regions for the K^+^ ions in simulations of the wild-type NSR system (left) and the C14+ mutant (right), both bound to RIO (the ligand is not displayed). (**B**) Structural details of the key ion-binding site in the wild-type NSR with the K^+^ ion in green (left). This ion binding site is completely abolished in the C14+ mutant. The RNA coordinating residues are labeled. The ion coordination is indicated by black dashed lines. The graph (right) shows normalized radial distribution functions of K^+^ ions pair-wise distances (*r*) for the individual coordinating atoms in the wild-type simulations with RIO.

While the U14C+ mutation caused only very minor changes to the internal structure of the RNA molecule itself (see above), the MD simulations have revealed a major change in the solvent behavior in the vicinity of the mutated base. The data suggest that a profound difference in the ion-RNA interactions is counteracting the changes in the intrinsic RNA interactions caused by the U14C+ mutation, thus attenuating its impact. Specifically, we observed a strong K^+^ ion binding site in simulations of the wild-type NSR (close to 100% occupancy). This K^+^ ion was virtually always forming an inner-shell (direct) interaction with the U14(O4) atom and thus we call it the ‘U14(O4) ion-binding site’. Also, depending on its fluctuations, it formed either inner-shell or less commonly outer-shell (water-mediated) interactions with the U13(O4) and A17(OP2) atoms. Lastly, it formed an outer-shell interaction with the U18(O4) atom (Figure 5B). A very similar result was obtained also in simulations with Na^+^ ions (Supplementary Figure S6).

The U14(O4) ion-binding site was completely abolished by the U14C+ mutation and no new ion site was formed to replace it in the vicinity of C14+ (Figure 5A). In the wild-type simulations, the ion-binding site appeared to stabilize the U-turn signature interaction U14(N3)/A17(OP2) by facilitating an ionic bridge between the U14(O4) and A17(OP2) atoms. We suggest that the absence of this ion-binding site is also responsible for the slightly altered behavior of the U13/U18 base pair in C14+ simulations as the U13(O4) and U18(O4) atoms contribute to this ion-binding site in the wild-type simulations (Figure 5B). In other words, the lack of electrostatic screening from the ion site may alter the population of the U13/U18 base pairing arrangements in the C14+ simulations (see above and Table 3). Overall, the simulations suggest that the U14(O4) ion-binding site can be considered as an integral part of the network of interactions stabilizing the NSR. The role played by the protonated cytosine is thus dual; to keep the signature H-bond and to compensate for the missing positive charge of the bound ion.

**Figure 6.**
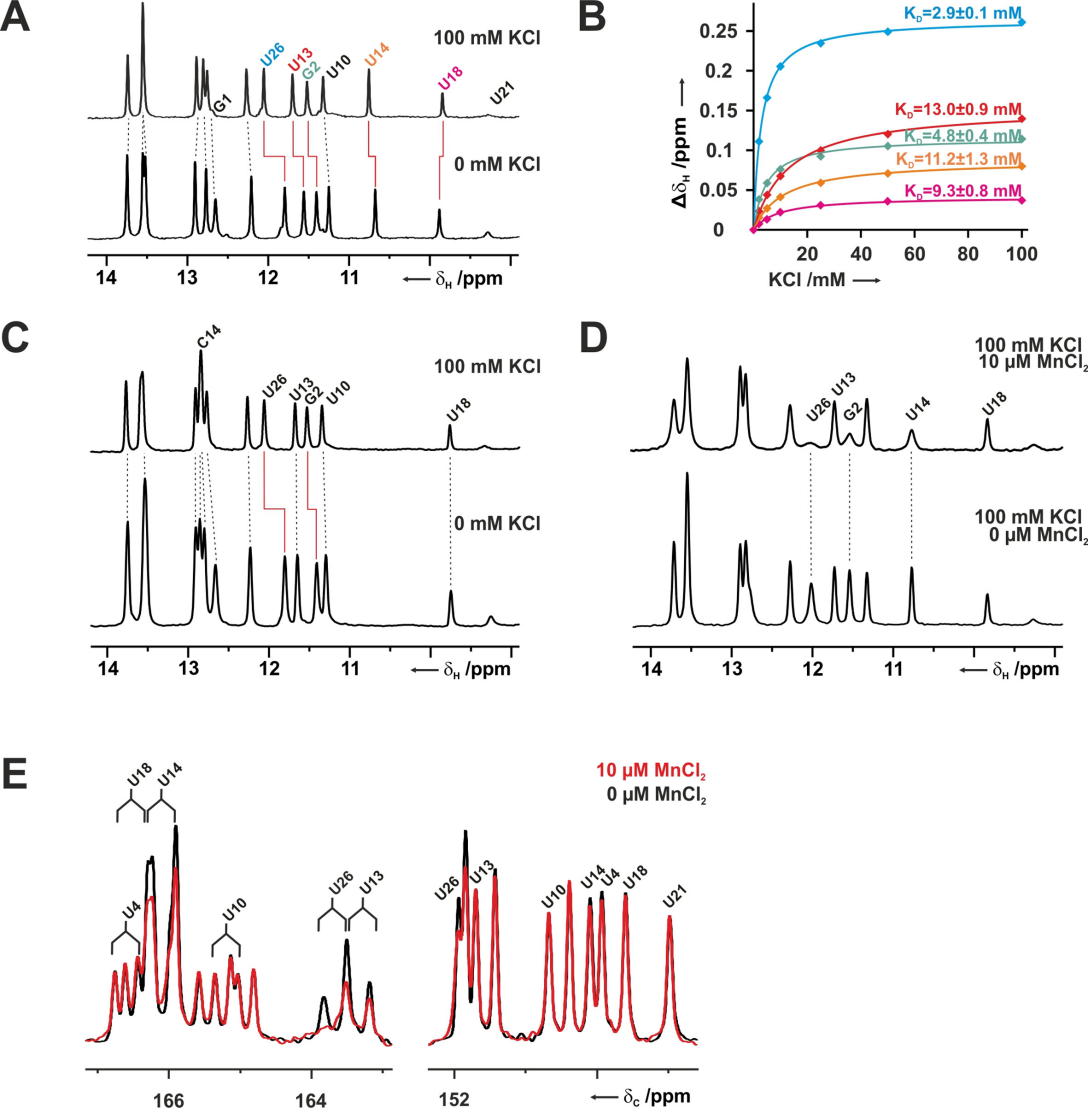
Potassium ion binding to the NSR/RIO complexes by NMR. (**A**) Chemical shift changes of the imino protons induced by the addition of 100 mM KCl to the wild-type NSR/RIO complex in 25 mM BisTris buffer at 10°C. (**B**) Chemical shift changes as a function of potassium ion concentration for G2 (red) and U26 (blue) of the G/U wobble base pair and U13 (green) and U14 (orange) from the U-turn loop. (**C**) Chemical shift changes of the imino protons induced by the addition of 100 mM KCl to the C14+ NSR/RIO complex in 50 mM BisTris buffer at 10°C. (**D**) The addition of 10 μM MnCl_2_ in the presence of 100 mM KCl led to selective line broadening for the imino proton signals of G2 and U26 as well as U14 for the wild-type NSR/RIO complex. (**E**) In an 1D-^13^C-direct detection experiment the addition of 10 μM MnCl_2_ (red spectrum) in the presence of 100 mM KCl led to selective line broadening for the C4 carbon signals (left) of U26 (G/U wobble base pair), U13 and U14 in the wild-type NSR/RIO complex. The C4 signals appear as doublets in this experiment due to the scalar coupling with C5. In contrast, signal attenuation due to line broadening of C2 signals (right) is only observed for unassigned signals that correspond to either U7 or U8 from the flexible bulge region.

The original NMR data for the wild-type NSR and the C14+ mutant were recorded exclusively in KH_x_PO_4_/KCl containing buffer and therefore did not allow conclusions about putative K^+^ ion-binding sites in this system (2,8). In order to experimentally investigate K^+^-binding to the NSR/RIO complex by NMR we exchanged the RNA/ligand complex into a 50 mM BisTris buffer at the same pH as used in the original study (pH 6.3) without any other added monovalent ions. Fortunately, the imino proton 1D-spectra of both the wild-type and the C14+ RIO complex in BisTris buffer were very similar to those recorded previously in the K^+^-containing buffers. This showed that the RNAs bound the ligand and adopted the same structure under these conditions. It also allowed the direct transfer of previous NMR signal assignments to the new conditions. Titration of the wild-type complex in BisTris-buffer with KCl led to significant chemical shift changes for the imino protons of a few selected nucleotides (Figure 6A). This included G2 and U26, which form a wobble G/U base pair and the neighboring G1. Wobble G/U base pairs are known K^+^-binding sites in general and bound stably to a K^+^ ion in all simulations (Figure 5A). In the spectra of the wild-type, the U13 and U14 imino protons showed large chemical shift changes upon K^+^-addition as well. Furthermore, the signals shifted downfield to higher ppm in agreement with stronger deshielding of the imino protons indicating an increase in the distance between the N and H of the donor imino group and a concomitant decrease in the distance between the proton and the hydrogen bond acceptor group. Thus, the chemical shift changes upon K^+^-titration do not only confirm potassium binding to the site predicted by the MD-simulations but also indicate a strengthening of the hydrogen bonding interaction network in the U-turn motif induced by K^+^-binding. The U18 imino proton shows only a very small upfield chemical shift change upon KCl addition. Plotting of the observed chemical shift changes against the KCl concentration yielded dissociation constants for the K^+^-interactions with this RNA (Figure 6B). The G2/U26 wobble base pair bound K^+^ with an apparent *K*_D_ of ∼ 3 mM whereas the U-turn motif bound K^+^ with an apparent *K*_D_ of ∼ 11 mM (U13, U14 and U18). Notably, for the C14+ NSR/RIO complex, addition of KCl only led to chemical shift changes for the G1, G2 and U26 imino groups but not for the C14+, U13 and U18 imino proton signals, suggesting that in the mutant system the K^+^ does not bind to the U-turn motif, in full agreement with the simulations (Figure 6C). To further verify that the U-turn motif in the wild-type is a hotspot for the binding of positively charged ions we titrated the RNA RIO complex with 10 μM MnCl_2_ in the presence of 100 mM KCl. Mn^2+^ is a paramagnetic ion inducing fast relaxation and subsequent line broadening for the NMR-signals of nuclei in close spatial proximity to its preferred binding sites (69,70). It is commonly used to map divalent cation binding sites in RNA (71) and is not a perfect mimic for K^+^-ions due to its significantly smaller ionic radius. However, it should be well suited to identify areas of high negative charge density in RNA structures. Addition of MnCl_2_ to the wild-type complex lead to line broadening for the G2 and U26 imino protons but also for the U14 imino signal in the U-turn supporting the results of the KCl titrations (Figure 6D). To directly probe interactions between Mn^2+^-ions and the C2 and C4 carbonyl oxygens of the uridine bases, 1D-^13^C-spectra were recorded for a selectively U-^13^C,^15^N-labeled RNA. Strong signal attenuations in the presence of 10 μM MnCl_2_ were observed for the C4 carbon signals of U26 (G/U wobble base pair), U13 and U14 (Figure 6E). On the other hand, the C2 carbon signal intensities of U13 and U14 were not affected by the presence of MnCl_2_ and only a small attenuation of the C2 signal intensity was seen for U26. Again, this is in agreement with the computational results that identified the O4 carbonyl groups of U13 and U14 as preferred coordination sites for cations (see above and Figure 5B).

### The potassium binding site near the conserved uracil is unrelated to known magnesium binding sites of the U-turn motif

The cation binding site in the vicinity of the U13, U14 and U18 C4 carbonyl groups identified in NSR by our MD simulations is in a somewhat similar location as a magnesium binding site predicted by NMR in a structurally related U-turn motif occurring in the stem loop V of the VS ribozyme (69). Since our NMR experiments with the NSR were conducted in the absence of Mg^2+^, this raises the possibility that the observed potassium binding site in the U-turn structure might instead be occupied by Mg^2+^ ions under more physiological ionic conditions (∼2 mM Mg^2+^, 150–200 mM K^+^). To explore this possibility, we titrated the NSR/RIO complex in 50 mM BisTris buffer at pH 6.3 with MgCl_2_. The addition of Mg^2+^ caused large chemical shift changes for the imino proton signals of G2, U13 and U18 and a less pronounced chemical shift change for U14 (Supplementary Figure S7A). Interestingly, under saturating conditions the maximum chemical shift change caused by MgCl_2_ was larger for the U13 and U18 but substantially smaller for U14 than the chemical shift change at saturating concentrations of KCl. This could indicate that the Mg^2+^ binding site is closer to U13 and U18 and further away from U14 than the K^+^ binding site. As expected, the affinities of divalent Mg^2+^ for the binding sites in the NSR/RIO complex were higher than those determined for the monovalent K^+^ ion. Fitting of binding isotherms yielded a K_D_ of 35 μM and ∼250 μM for Mg^2+^ binding to the G/U wobble base pair and the U13/U18 region, respectively. Interestingly, the dependence of the chemical shift changes on the MgCl_2_ concentration could only be fitted satisfactorily to a model where two Mg^2+^-ions bind cooperatively to each site (Supplementary Figure S7B).

In order to probe if K^+^ could compete with Mg^2+^ for the binding site in the U-turn the sample containing saturating Mg^2+^ concentrations (2.5 mM) in 50 mM BisTris buffer, pH 6.3, was then titrated with KCl. Interestingly, KCl addition lead to further chemical shift changes of the U14 imino resonance similar in size and directions to those observed in the absence of MgCl_2_ (Supplementary Figure S7C). The *K*_D_ for K^+^ for this site in the presence of MgCl_2_ is 19 mM and therefore only approximately two-fold higher than in the absence of MgCl_2_. This suggests that the U-turn motif of the NSR/RIO complex contains a K^+^-binding site located in the proximity of U14 that is independent of the Mg^2+^-binding. In contrast, the U18 imino proton chemical shift changed only into the direction observed in the absence of Mg^2+^ upon addition of larger amounts of KCl resulting in a *K*_D_ for K^+^ under these conditions of ∼218 mM. This suggests a scenario where under physiological salt conditions the U-turn motif of the NSR is stabilized by a K^+^-ion bound close to U14 and a preferentially Mg^2+^-bound site in the vicinity of U18.

Note that we ultimately refrained from performing MD simulations of the NSR in presence of the Mg^2+^ ions. The force-field performance for the divalent ions is less reliable since these ions involve significant elements of polarization and charge-transfer effects, both of which are neglected by currently used force fields (14). Further, Mg^2+^ ions represent a major challenge also in terms of sampling, especially since the experimentally used ∼2 mM concentration of Mg^2+^ ions would correspond to only ∼0.2 Mg^2+^ ions per simulation box (14). Thus, the behavior of the Mg^2+^ ions in simulations is sometimes over-interpreted in the literature and we decided to not attempt simulations on the K^+^ versus Mg^2+^ competition, since we assume that such investigations would be well beyond the accuracy limits of the MD simulation methodology (14). We have nevertheless performed additional MD simulations with Na^+^ ions instead of K^+^ (Table 1), obtaining very similar results with both ion types (Figure 5 and Supplementary Figure S6), including the crucial U14(O4) ion-binding site. This shows that formation of the key cation-binding sites in NSR simulations is not limited only to K^+^.

### The QM/MM analysis of the signature H-bond in the U14 and C14+ systems—general comments

The MD simulations are unsuitable to study electronic structure effects associated with the different electronic environments of the U14(N3)/A17(OP2) and C14+(N3)/A17(OP2) H-bonds as these are only indirectly described by the MM model through Coulomb electrostatics and Van der Waals terms (14). A hallmark of H-bonding electronic structure effects is the elongation of the X–H bonds, where X is the donor heavy atom; for more details see, e.g. (72)). The force field substantially underestimates this effect and cannot reflect its true physical (molecular orbital) origin. This may affect the accuracy of an MD description of the H-bonding (73) and the simulations alone cannot account for elongation and polarization of the N3–H3 bond, which could substantially differ for U14 and C14+ bases.

Therefore, we have used QM/MM optimizations of carefully selected simulation snapshots of the complete solvated NSR to further investigate the geometry of the U14(N3)/A17(OP2) and C14+(N3)/A17(OP2) interactions (see the Methods and Table 4). Standard MM optimizations of the same systems were performed for comparison. Lastly, isolated uracil and protonated cytosine bases were optimized by MM and QM to get reference nucleobase geometries for the N3–H3 bond length. To evaluate the X–H bond elongation upon H-bonding, the reference value was subtracted from the N3–H3 bond length in structures with formed H-bonds.

**Table 4.** Geometry parameters of the U14(N3)/A17(OP2) and C14+(N3)/A17(OP2) H-bonds in the optimized structures

	**N3–H3 bond and interatomic distances (in Å)**					
	**N3–H3**		**H3/OP2**		**N3/OP2**	
**Method^a^**	**MM**	**QM/MM**	**MM**	**QM/MM**	**MM**	**QM/MM**
**System**						
2n0j_wt	1.011	1.034	1.86	1.84	2.86	2.87
2n0j_wt_K^+^	1.010	1.036	1.78	1.75	2.76	2.77
2n0j_C14+	1.010	1.047	1.78	1.65	2.73	2.66

**Method^a^**	**MM**	**QM**				
**System**						
Uracil base (reference)	1.004	1.017				
Cytosine+ base (reference)	1.003	1.017				

^a^Method used to perform the optimization: MM—molecular mechanics (bsc0_χOL3_); QM—TPSS-D3; QM/MM—TPSS-D3 in the QM part of the system and bsc0_χOL3_ in the MM part of the system. The reference MM and QM optimizations of isolated bases were performed in continuum solvent as implemented in AMBER (40,74) and in COSMO model (75), respectively. All other calculations (QM/MM and MM of the complete NSR) were performed in explicit SPC/E solvent.

### The QM/MM calculations indicate different N3–H3 bond elongation in U14 and C14+ systems

The QM/MM calculations of the fully solvated NSR system showed elongation of the N3–H3 bond upon H-bond formation of +0.017 Å and +0.030 Å for the wild-type and C14+ systems, respectively (Table 4). This confirms that the protonated cytosine forms a considerably stronger H-bond in terms of the local electronic structure redistributions. In contrast, pure MM optimizations showed significantly smaller and undifferentiated X-H bond elongations compared to QM/MM, giving N3–H3 bond elongation values of +0.007 Å for both uracil and protonated cytosine (Table 4). This suggests that during the MD simulations, which in addition typically apply the SHAKE algorithm, the force field underestimates the geometrical and energy differences between the U14 and C14+ N3–H3/OP2 H-bonding.

### QM/MM calculations and NMR experiments suggest an ion-assisted binding for the U14(N3)/A17(OP2) interaction

The QM/MM calculations showed that both the N3/OP2 and H3/OP2 interatomic distances are shorter in the C14+ system than in the wild-type U14 system (Table 4). However, the inclusion of the highly-occupied U14(O4) binding-site K^+^ ion (commonly observed in the wild-type MD simulations; see above and Figure 5) into the calculation somewhat lowers this difference. The presence of the K^+^ ion in the wild-type system renders the U14(N3)/A17(OP2) interaction geometry more similar to the C14+(N3)/A17(OP2) interaction (Table 4). It also slightly alters the bond orders in the uracil base (Supplementary Figure S8). Lastly, QM calculations of small model systems representing the U14(N3)/A17(OP2) and C14+(N3)/A17(OP2) interactions (see Supplementary Data) show that although the interaction energy is much greater for the C14+ system than for the isolated U14 system, inclusion of the K^+^ ion into the U14 system makes its interaction energy similar to the C14+ system. Thus, the QM/MM and QM calculations clearly reveal the active role played by the U14(O4) ion-binding site in the overall balance of the interactions.

**Figure 7.**
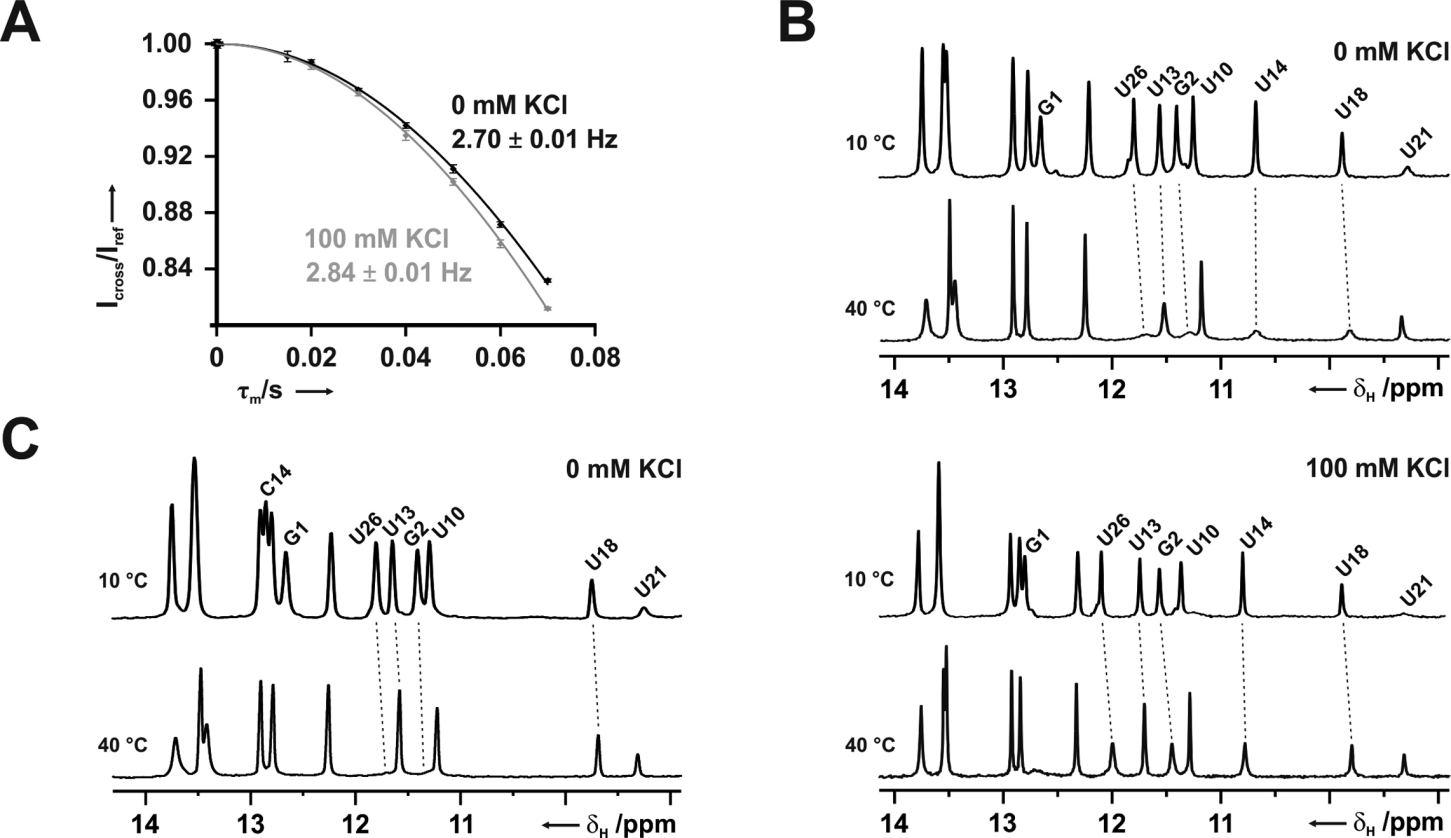
Influence of K^+^ on hydrogen bonding and U-turn stability. (**A**) Signal intensities ratios for U14 imino proton signal in the cross and reference experiments of a 1D-^1^H-constant time (τ_m_) experiment with and without ^31^P-decoupling for the measurement of the ^2h^J_H,P_-cross hydrogen bond scalar coupling between the U14 imino proton and the backbone phosphate group in 50 mM BisTris, pH 6.3. Measurements were performed in the absence and presence of 100 mM KCl, respectively. (**B**) Temperature dependent 1D-^1^H-imino proton spectra at 10 and 40°C for the wild-type NSR RIO complex in the absence (top) and presence (bottom) of 100 mM KCl, respectively. (**C**) Temperature dependent 1D-^1^H-imino proton spectra at 10 and 40°C for the C14+ NSR RIO complex in the absence of KCl.

These theoretical results are in good agreement with previous NMR-results (13) where the ^2h^J_H,P_ and ^3h^J_N,P_ scalar couplings across the hydrogen bond between the U14/C14+ imino group and the phosphate were larger for the C14+ NSR complex than for the wild-type, suggesting shorter N–P and H–P distances across this hydrogen bond in the mutant. However, in order to directly probe the influence of potassium binding on the hydrogen bond geometry in the wild-type system, as suggested by the QM/MM and QM calculations, we re-measured the ^2h^J_H,P_ cross hydrogen bond coupling constant for the wild-type NSR/RIO complex in BisTris-buffer, with and without 100 mM KCl, respectively. While the measured coupling constant was ∼2.7 Hz in the absence of KCl, it increased to ∼2.85 Hz in the presence of 100 mM KCl (Figure 7A), in agreement with the observed shortening of the H–P distance across the hydrogen bond in the QM calculations (Table 4). The corresponding coupling constant in the C14+ system was even larger (13).

The calculations also clearly predict that the U14(O4) ion-binding site is important for the thermodynamic stability of the wild-type NSR U-turn. In other words, lacking potassium ions, the U-turn motif in the C14+ system might be more stable than in the wild-type RNA, while the addition of potassium ions should lead to a local stabilization in the wild-type. This prediction is easily testable by simple 1D-NMR experiments in BisTris-buffer recorded at different temperatures since the imino protons exchange with the solvent protons. This exchange leads to line broadening which decreases signal intensities. The decrease of signal intensities with rising temperatures is more severe for less stable hydrogen bond interactions. Comparison of imino proton spectra for the wild-type NSR at 10 and 40°C in 50 mM BisTris buffer, pH 6.3, in the absence of potassium ions shows that at 40°C the imino proton signals for G1, G2, U26 at the end of the RNA helix as well as those for U13, U14 and U18 in the U-turn and its closing U/U base pair are severely broadened (Figure 7). Note that this is not due to loss of ligand binding since the signals of U10 and U21 in the center of the ligand binding site remain observable. By contrast, in the presence of 100 mM KCl the broadening of the imino proton signals of G2, U26, U13, U14 and U18 is much less severe (Figure 7B), in agreement with a substantial potassium ion-induced thermodynamic stabilization of these structural elements, as suggested by the calculations. In contrast, for the C14+ system the intensities of the U13 and U18 imino protons are only slightly affected by raising the temperature from 10 to 40°C suggesting a stable U-turn structure even in the absence of KCl (Figure 7C). Comparison of imino proton spectra of the wild-type NSR/RIO complex at 10°C and 40°C in the absence or presence of 100 mM NaCl shows that sodium ions are also able to stabilize the U-turn structure (Supplementary Figure S9). Nevertheless, based on detailed analysis of the chemical shift changes induced by NaCl addition we cannot rule out some subtle differences in the mode of sodium ion binding to the U-turn compared to potassium. Unfortunately, with the available experimental means, details of such differences remain elusive. MD simulations (see above) suggest essentially identical binding of sodium and potassium to the U-turn. However, the force-field description might not be quantitative enough to unmask subtle variability of the sodium vs. potassium binding due to the general force-field approximations (14).

### A neutral C14 base combined with a protonated phosphate is an energetically unfavorable state

Lastly, we used QM calculations to explore the possibility of a neutral C14+ system where the A17 phosphate group would be protonated and the cytosine 14 in the canonical form, thus satisfying the same signature H-bond interaction. The large p*K*_a_ difference (4.2 and 2.0 for the cytosine N3 atom and backbone phosphate group, respectively) (9) would make such a protonation state very rare but it was shown that even rare protonation states can influence RNA biochemistry (76). In this particular case, the computations confirm that formation of the protonated phosphate in the C14+ NSR complex is highly unfavorable, as expected from the p*K*_a_ values of the participating groups, though we do not entirely rule out that this state could be transiently reached at room temperature. Full details of the computations are given in Supplementary Data.

## DISCUSSION

### Combination of diverse computations and NMR measurements reveals an intricate balance of forces that is responsible for the smooth U14C+ substitution in NSR

In this work, we present a comprehensive multiscale computational and NMR experimental study of the neomycin sensing riboswitch (NSR), the smallest known biologically functional riboswitch (32). We used series of μs-scale explicit solvent MD simulations to examine its structural dynamics. In particular, we examined the impact of the U14C+ mutation of the conserved uracil in the U-turn loop, which has been experimentally shown to be benign to the riboswitch structure and function. This mutation has been shown to be structurally and functionally acceptable in other U-turn motifs as well (8). We also used QM and large-scale QM/MM calculations to characterize in more detail the physical nature of the key molecular interaction in the NSR affected by the mutation, namely the N3/OP2 U-turn signature H-bond (Figure 1B). Finally, we used a series of NMR spectroscopy measurements to corroborate the results obtained by the computations.

The main message of our work is that in order to fully understand the essence of the seamless U14C+ substitution in the U-turn of the NSR, we had to use an unprecedented portfolio of advanced computations and NMR measurements. This combination of techniques unmasked an intricate and context-dependent interplay of diverse molecular interactions and their mutually compensatory role. We suggest that complex interrelations of energy contributions might be rather common in nucleic acids and may have been so far overlooked, as it is not possible to describe them when using just one type of technique. In other words, molecular interactions shaping up nucleic acids may be sometimes much more complicated than commonly assumed, which may, for example, contribute to difficulties when attempting to predict structure, thermodynamics and other properties of nucleic acids from their sequences in a quantitative manner (14,77–79).

### The U14C+ mutation dramatically alters the RNA interaction with ions

The U14C+ mutation had very little impact on the structure of the NSR RNA itself except for a slightly altered behavior of the U13/U18 base pair (Table 3). Further, the simulations predicted a shorter anion-π interaction distance for the C14+ system than for the U14 system (Figure 4). This compaction can be explained by the different charge distributions of the U14 and C14+ bases, namely the +1 positive net charge distributed over the C14+ base.

The most significant change revealed by the simulations was, however, the altered distribution of the cation environment caused by the C14+ mutation. Specifically, a highly-occupied inner-shell (chelated) monovalent cation-binding site seen in the wild-type simulations at the U14(O4) atom was completely abolished in the C14+ simulations (Figure 5). Further, the NMR experiments showed a strong response to changes in potassium concentration associated with the U14 nucleotide whereas no such change was observed for the C14+ nucleotide (Figure 6).

Interestingly, the *c*WW U13/U18 closing base pair of the U-turn in the NSR system was also involved in coordination of this ion site. This could potentially mean that this ion site would be altered or non-existent in other systems containing U-turns since the closing base pair can be different. For example, in many U-turns, there is a *t*HS A/G as the closing base pair instead of *c*WW U/U (3). Still, NSR simulations strongly suggest that the O4 atom of the conserved uracil (U14 in the NSR) is the most significant atom for the ion coordination (see Figure 5B). We subsequently conducted control MD simulations of two additional RNA systems containing the U-turn motif (see Table 1) with a different closing base pair. Importantly, even in these systems we still observed a highly occupied monovalent ion-binding site coordinated by the O4 atom of the conserved uracil (Supplementary Figure S10), similar to our observations in the NSR. Lastly, our NMR experiments showed that magnesium ions do not supplant the potassium at this ion site in the NSR/RIO complex and that potassium is bound in the vicinity of U14 even in the presence of Mg^2+^. Thus, we suggest that this monovalent cation-binding site may be characteristic of the U-turn motif structure in general. Strangely though, our database search of the RNA structural motifs using the FR3D program (80) revealed only a few examples of RNA structures with U-turn motifs displaying such ion-binding sites. Specifically, there is a K^+^ ion near residue 14 in the 16S rRNA X-ray structure (PDB: 2vqe) (81) and a lysine side-chain near residue 11 in some frames of the NMR ensemble of the bacteriophage Phi21 N Peptide-boxB RNA complex (PDB: 1nyb) (82). There is also a Mg^2+^ ion near residue 14 in the structure of the *Thermus thermophilus* 30S ribosomal subunit (PDB: 1ibl) (83). A possible explanation of this is that while the ion-binding site showed nearly 100% occupancy in the MD simulations, it was also in a fast-exchange regime with the bulk solvent with a maximum observed binding time of 3.3 ns and an average residence time of ca. 250 ps. In other words, it is a high occupancy but short-binding-time cation-binding site (14). The high occupancy means that it is strongly favored in terms of free energy but it is not separated from the bulk by a high free-energy barrier. Due to its inherently dynamical behavior, this ion-binding site might be systematically underreported in the X-ray structures due to the difficulty in identifying such dynamical elements in the electron density maps. Allocation of monovalent and divalent cations (or even water molecules) might also be uncertain in mid-to-lower resolution X-ray structures (84). On the other hand, simulations are well suited for the study of dynamical ion binding, provided a reasonable force-field selection and a careful comparison with experimental data is made (14). In the past, MD simulations were successfully used to predict and study dynamical ion binding sites in RNA (14,85) and simulations have already previously shown that the U-turn motif can bind Mg^2+^ ions (19,20).

### Multi-scale computations and NMR experiments suggest ‘ion-assistance’ in stabilization of the U14(N3)/A17(OP2) signature H-bond which is replaced by ‘excess-charge assistance’ after the U14C+ mutation

Using the QM/MM optimizations, we have provided a physical explanation for the response of the N3/OP2 signature interaction to the U14C+ mutation. The C14+(N3)/A17(OP2) H-bond interaction distance observed in the mutant system is shorter than the wild-type U14(N3)/A17(OP2) interaction (Table 4). The N3–H3 bond elongation upon H-bond formation is larger for the system with protonated cytosine. In other words, the C14+(N3)/A17(OP2) interaction is stronger than the U14(N3)/A17(OP2) interaction due to a partial ionic-bonding character (negative phosphate, positive base) which leads to a shortening of the H-bond. However, when including the nearby K^+^ ion observed in the wild-type MD simulations (see above and Figure 5), the U14(N3)/A17(OP2) H-bond is also compacted as the K^+^ cation provides a similar ionic charge distribution of the backbone/base interaction as in the C14+ system (Table 4). This interpretation is further supported by QM computations on small model systems (Supplementary Data) and by new NMR measurements (Figure 7). Note that this information could not be inferred from the MD simulations, as the force-field description does not include the electronic structure changes accompanying the H-bond formation.

In conclusion, the presence of the U14 cation-binding site in the wild-type structure reduces the differences in the local interaction networks between the U14 and C14+ systems. Thus, the interactions after the U14C+ substitution mimic the native U14 system much more closely than expected when considering only the isolated RNA molecule. The two systems could, however, respond differently to varying salt and pH conditions.

### Comment on the accuracy of the description of the NSR system in MD simulations

With close to 99% satisfied NOE distances (Table 2), the NSR system was very well described by the bsc0χ_OL3_ force field (21,41,42). The only exception was the O2′/N7 U-turn signature H-bond that was sometimes unstable in simulations as the ribose hydroxyl group formed an interaction with the nearby phosphate group instead (Supplementary Figure S3). However, this change did not destabilize the remainder of the U-turn structure and was reversible, indicating only a subtle force-field imbalance. One of the reasons for the observed local discrepancy between the MD simulations and the primary NMR data could be the over-stabilization of the hydroxyl/phosphate interactions. This is a known imbalance in RNA simulations with current AMBER RNA force-field versions (61,86,87). There have been some attempts to improve the balance of interactions in RNA simulations (14,61,86–88) by adjusting the VdW Lennard-Jones parameters and/or by using different water models (59,60). As detailed in the Supplementary data, in our particular case the suggested modified phosphate parameters and water model did not fix the problem. In contrast, the simulation behavior could be corrected by application of the recently suggested HBfix potential term, gently supporting the native O2′/N7 interaction (Supplementary Table S1) (14,61). This confirms that the imbalance of the force field is quite small in this particular case.

In full agreement with the experiments (32), the presence of the RIO ligand stabilized the NSR structure in simulations. We have noticed increased RNA thermal fluctuations in the simulations without the bound ligand (Figure 2) and ultimately observed partial but permanent perturbation of its structure on an extended, ten-microsecond timescale (Supplementary Data). In contrast, the NSR with the bound ligand remained entirely stable during the extended simulations, just as suggested by the experiments (32).

In summary, upon a comparison with the available RNA simulation literature (14) we suggest that the NSR/RIO complex is one of the most satisfactorily behaving RNA systems in MD simulations with the applied AMBER force fields. The plentitude of its primary NMR data can provide a useful benchmark for testing of future force-field modifications.

### Concluding remarks

Our study confirms the high value of MD simulations in complementing experimental methods such as NMR measurements in studies of biomolecular systems. Some of the presented data, such as the description of the integral ion binding site near the U-turn signature interaction, would be highly difficult to obtain using standard experimental methods for structure determination. We obtained stable MD trajectories for both the wild-type and mutated NSR and thus provided structural background for the data observed in the biochemical experiments. The simulations were in very good agreement with preceding NMR studies and motivated us to carry out additional in-depth NMR investigations. At the same time, we show that judicious application of QM/MM multi-scale methods can alleviate some of the inherent limitations imposed by the approximations of the force fields used in MD simulations. Overall, the combination of advanced computations and NMR experiments explains why the protonated cytosine can so efficiently replace uracil in the NSR and in similar RNA structures containing the U-turn motif. In the studied RNA context, the different distribution of donor and acceptor groups of the uracil and protonated cytosine moieties and their different formal charge are counterbalanced by the interplay with the complementary cation environment around the RNA. Our in-depth study reveals an amazing complexity of the molecular interactions involved in the stabilization of the NSR U-turn structure. We suggest that such a complex interplay between diverse molecular interactions may be common in nucleic acids.

## Supplementary Material

Supplementary Data
