# Supplementary material for: An intricate balance of hydrogen bonding, ion atmosphere and dynamics facilitates a seamless uracil to cytosine substitution in the U-turn of the neomycin-sensing riboswitch
Source: Nucleic Acids Res. 2018 Jun 9;46(13):6528–43. doi: 10.1093/nar/gky490 (PMC6061696; doi:10.1093/nar/gky490)
Supplement: Supplementary Data [file gky490_supplemental_files.docx]

# Supplementary Information Methods

**Comments on use of NMR structures in MD simulations and selection of the starting structure from the NMR ensemble.**

The use of biomolecular structures determined by NMR spectroscopy as starting structures in MD simulations can be less straightforward than use of structures determined by other experimental methods (e.g. X-ray crystallography and Cryo-Electron Microscopy). The difference is that distance and torsion angle restraints derived from solution NMR spectroscopy experiments can almost never be unambiguously converted into a single structural model of the studied biomolecule. Thus, the NMR structures are typically deposited in the database as an ensemble of structures where each individual model (frame) represents an alternative structural model which fits the available experimental data. In practice, for state-of-the-art high quality NMR structures with an abundance of distance and torsion angle restraints, this gives a set of highly similar structures with structural differences mostly localized in the flexible parts of the biomolecule for which too few or no distance and torsion angle constraints could be derived from the NMR experiments.

Therefore, one of the issues can be selection of the starting structure for MD simulations from among the frames of the deposited NMR ensemble. Since it would be impractical to simulate all the available structural models from the NMR ensemble, in most cases, a single model needs to be selected as the starting structure. One of the possible approaches is manual inspection of the NMR ensemble and selection of the model containing the structural features which are of interest to the study. This approach is especially useful for poorly restrained NMR ensembles with larger differences between the individual models. However, depending on the project’s specifics, starting the simulations from two or more models from the NMR ensemble may be necessary if they differ to a point where a biased result may be obtained if simulations were started only with one model. Note that with poorly determined NMR structures, the refined NMR structures can be dramatically affected by the refinement protocol whereas for NMR structures based on large sets of restraints the influence of the refinement protocol should become negligible.

For NMR structures where structural differences among the NMR ensemble models are minor or negligible (such as the NSR studied in this work), it is often advantageous to simply start the MD simulations from the first frame of the NMR ensemble. Depending on the specific NMR refinement protocol used by the experimentalists, the first frame sometimes corresponds to the structural model with the lowest potential energy, the least amount of restraint violations or it represents the cluster with largest population if some clustering algorithm was applied to obtain the NMR ensemble during the refinement. However, in high quality NMR structures with large amounts of experimental constraints and low RMSD values between structures, the differences between the individual structures in this regard should be minor. We generally do not recommend starting MD simulations from averaged structure computed from all frames of the NMR ensemble as here atom positions are averaged and therefore for instance standard bond lengths and angles are not kept. Thus, this can lead to an unphysical starting geometry, which is very high in energy and may “blow up” in subsequent simulation.

Lastly, note that the potential selection bias associated with the MD simulation starting structure selection for NMR structures is largely rendered insignificant by applying simulation protocol in which the NMR restraints are temporarily used in the initial portions of the production simulations (see below for details). At the same time, this approach can dramatically increase the stability of NMR structures in subsequent unrestrained MD simulations ([1](#_ENREF_1)) and we recommend its application whenever possible.

Note that the NSR structure can be considered as a high-quality experimental structure based on the large number of distance and torsion angle restraints obtained to determine this structure as well as on the low RMSD value between the different structures in the NMR ensemble. This is also subsequently confirmed by the neat performance of the MD simulation technique.

**MD simulations protocol.**

The minimization and equilibration of the solvated NSR system was done by the sander.MPI program of AMBER16 in a series of alternating minimization and equilibration runs with gradually decreasing positional restraint applied to the solute.

Specifically, the systems prepared in tLeap were first subjected to a cycle of 1000 minimization steps with solute positional restraint of 25 kcal/mol/Å^2^ applied. Note that first 500 steps of all minimization runs were done with the steepest descent method while switching to the conjugated gradients method for the rest of the run. The first minimization was followed by NVT (constant volume) 100 ps-long simulation run in which we gradually heated the system from 100 K to 300 K with the same positional restrained applied as in the previous step. The Berendsen weak-coupling thermostat with a default coupling constant of 1 ps was used to regulate the temperature in this simulation. This was followed by another cycle of 1000 minimization steps with solute positional restraint lowered to 5 kcal/mol/Å^2^. Afterwards, we followed with NPT (constant pressure) 50 ps-long simulation run with the same positional restraint applied as in the previous step. The Berendsen weak-coupling barostat and thermostat were used to regulate the pressure and temperature, respectively, using the coupling value of 0.2 ps for both. Afterwards, the cycles of minimizations and constant pressure 50 ps-long simulation runs were repeated with the same settings but with gradually lowering solute positional restraint of 4, 3, 2, 1, and 0.5 kcal/mol/Å^2^, respectively.

After the initial equilibration of the systems, the first 120 ns of the production simulations were computed with the pmemd module (CPU implementation) of AMBER 16. Standard flat-well distance restraints based on the experimentally measured NOE upper bound hydrogen-hydrogen distances were used to stabilize the initial phase of the simulations (for details of this procedure, see the Ref. ([1](#_ENREF_1))). After 120 ns, completely unrestrained simulations were executed using the pmemd.cuda (GPU-based SPFP implementation) module ([2](#_ENREF_2),[3](#_ENREF_3)). Note that only the fully unrestrained parts of MD trajectories (the period beyond 120 ns) were used for the analyses (see main text), to avoid any bias of the simulation ensemble results by the restraints ([4](#_ENREF_4)). In all MD simulations, we have used the particle mesh Ewald method ([5](#_ENREF_5),[6](#_ENREF_6)) to calculate the electrostatic interactions and applied periodic boundary conditions. The cut off distance for the non-bonded Lennard-Jones interactions was set to 9 Å. We have used the SHAKE algorithm ([7](#_ENREF_7)) along with the hydrogen mass repartitioning ([8](#_ENREF_8)), allowing a 4 fs integration step. The Berendsen weak-coupling ([9](#_ENREF_9)) thermostat with a coupling value of 1 ps and the Monte-Carlo barostat ([10](#_ENREF_10)) with an average exchange ratio of 31% were used to maintain the systems at a temperature of 300 K and a pressure of 1 bar, respectively.

In specific simulations, we have used alternative Van der Waals parameters for phosphates as defined by Case et al. ([11](#_ENREF_11)) in combination with OPC waters ([12](#_ENREF_12)) instead of SPC/E. In addition, we have used the HBfix potential function ([13](#_ENREF_13)) in some simulations to stabilize one of the signature H-bond interactions of the U-turn motif (see main text Figure 1C). The HBfix potential is a weak short-range structure-specific bias of selected H-bonds aiming to compensate for missing polarization effects and other deficiencies of the MM description of H-bonds. It has been successfully applied to achieve folding of a GNRA tetraloop ([13](#_ENREF_13)) and to stabilize the U1A protein/RNA interface ([14](#_ENREF_14)). The details of HBfix are extensively described in Ref. ([13](#_ENREF_13)).

**QM/MM calculations of the small model systems - methods.**

Bulk water solvation effects in QM calculations of smaller model systems without explicit-solvent sphere were approximated by the COSMO model ([15](#_ENREF_15)). This continuum solvent approximation describes the dielectric mean-field effects of a solvent environment. For the interaction energy calculations of the model systems, we used the hybrid functional B3LYP ([16](#_ENREF_16)) together with COSMO solvation to treat the zwitterionic model systems with a higher accuracy. The full level of theory was COSMO-B3LYP-D3/def2-TZVP. The energies were calculated on structures previously optimized by the COSMO-TPSS-D3/def2-TZVP method. All DFT-D3 calculations employed Becke-Johnson damping ([17](#_ENREF_17),[18](#_ENREF_18)).

# Supplementary Information Results

**List of average simulation NOE violations greater than 0.3 Å for the individual simulation ensembles:**

*(hydrogen atom names, violated distance in Å)*

**2n0j_wt:**

U14(HO2')/U18(H1') 0.431

U14(HO2')/U18(H3) 0.656

C6(H6)/G9(H8) 0.583

U14(H2')/A16(H61,H62) 0.564

U14(HO2')/U18(H4') 1.091

U14(HO2')/U18(H5) 0.720

U14(HO2')/U18(H6) 1.812

G19(H8)/G20(H1') 0.674

**2n0j_RIO_wt:**

U15(H4')/A16(H8) 0.319

G9(H1)/RIO(H21) 0.501

C6(H6)/G9(H8) 2.602

U14(H2')/A16(H61,H62) 1.478

U14(HO2')/U18(H4') 0.738

U14(HO2')/U18(H5) 0.950

U14(HO2')/U18(H6) 1.131

A16(H2')/A17(H2') 0.491

**2n0j_RIO_wt_CaseP_OPC:**

U14(HO2')/U18(H3) 0.384

U15(H4')/A16(H8) 0.372

G9(H1)/RIO(H21) 0.524

C6(H5)/C6(H3') 0.444

C6(H6)/G9(H8) 2.704

U14(H2')/A16(H61,H62) 1.871

U14(HO2')/U18(H4') 0.651

U14(HO2')/U18(H5) 1.235

U14(HO2')/U18(H6) 1.248

A16(H2')/A17(H2') 0.680

**2n0j_RIO_wt_HBfix_A:**

G9(H1)/RIO(H21) 0.443

C6(H6)/G9(H8) 2.737

U7(H4')/U7(H5) 0.325

G9(H5'')/U10(H5) 0.535

G9(H4')/U10(H5) 0.509

U14(H2')/A16(H61,H62) 1.456

U14(HO2')/U18(H5) 0.713

U14(HO2')/U18(H6) 0.642

A16(H2')/A17(H2') 0.502

**2n0j_RIO_wt_HBfix_B:**

G9(H1)/RIO(H21) 0.508

C6(H6)/G9(H8) 2.593

U14(H2’)/A16(H61,H62)1.354

U14(HO2’)/U18(H5) 0.465

U14(HO2’)/U18(H6) 0.470

A16(H2’)/A17(H2’) 0.381

**2n0j_RIO_wt_HBfix_C:**

G9(H1)/RIO(H21) 0.518

C6(H6)/G9(H8) 2.829

U7(H4’)/U7(H5) 0.322

U14(H2’)/A16(H61,H62)1.347

U14(HO2’)/U15(H4’) 0.379

U14(HO2’)/U18(H5) 0.503

U14(HO2’)/U18(H6) 0.462

A16(H2’)/A17(H2’) 0.387

**2n0j_RIO_wt_Na:**

U14(HO2')/U18(H3) 0.474

U15(H4')/A16(H8) 0.370

G9(H1)/RIO(H21) 0.507

C6(H6)/G9(H8) 2.646

U14(H2')/A16(H61,H62) 2.054

U14(HO2')/U18(H4') 0.910

U14(HO2')/U18(H5) 1.211

U14(HO2')/U18(H6) 1.349

A16(H2')/A17(H2') 0.566

**2n0j_C14+:**

C12(H42)/U18(H3) 0.534

U13(H6)/U18(H3) 0.612

U14(HO2')/U18(H1') 0.850

G9(H4')/U10(H5) 0.724

U13(H5)/U18(H3) 0.759

U14(H2')/A16(H61,H62) 1.415

U14(HO2')/U18(H4') 1.560

U14(HO2')/U18(H6) 0.692

G19(H8)/G20(H1') 0.643

**2n0j_RIO_C14+:**

U14(HO2')/U18(H1') 0.436

U14(HO2')/U18(H3) 0.559

G9(H1)/RIO(H21) 0.503

C6(H6)/G9(H8) 2.630

U14(H2')/A16(H61,H62) 2.125

U14(HO2')/U18(H4') 1.095

U14(HO2')/U18(H5) 1.472

U14(HO2')/U18(H6) 1.584

**Commentary about the specific NOE violations**: The NOE violations consistently observed in all simulation ensembles were those related to the U14(OH2′) atom. These violations were caused by the incorrect simulation description of the U14(O2′)/A16(N7) signature H-bond interaction which was sometimes reversibly replaced by the spurious U14(O2′)/U15(O5′) interaction (see below). The same problem was the cause of the U14(H2')/A16(H61,H62) NOE violation, which was also universally observed. The list shows that these violations could be reduced in number and size by application of the HBfix potential (the 2n0j_RIO_wt_HBfix_A/B/C simulation ensembles). In simulations which included the RIO ligand, we universally observed the G9(H1)/RIO(H21) NOE violation which was, however, relatively minor on the scale of ~ 0.5 Å. The NSR/RIO simulations also showed over 2 Å-NOE-violation for the C6(H6)/G9(H8) interatomic distance. This NOE violation was much smaller or non-existent in simulations without the RIO ligand. For this single hydrogen-hydrogen distance, the simulations may have revealed a minor struggle to exactly reproduce the RNA geometry within the confines imposed by the bound ligand. However, based on the NMR-data, nucleotide C6 is partially flexible ([19](#_ENREF_19)) which renders a quantitative interpretation of NOEs in this region difficult. The remainder of the listed NOE violations was minor and random in character, i.e. not universally observed in the simulation ensembles. Note that there were no NOE violations which would be uniquely observed only for the wild-type or the mutant systems.

**Summary of the extended simulations of the NSR system**

There is experimental evidence that the structure of the NSR is much less stable without the bound ribostamycin (RIO) ligand. Therefore, when performing simulations of the NSR system (PDB: 2n0j) with the ligand removed by molecular modeling, the RNA can be expected to eventually unfold. Our initial one-microsecond scale simulations (main text Table 1) have shown increased thermal fluctuations of the RNA in absence of the ligand but no unfolding events. Therefore, we decided to extend the timescale of four selected simulations (wild-type and C14+ systems, with and without RIO, respectively) up to 10 μs. These simulations have finally shown partial unfolding of the NSR systems without RIO. Specifically, the characteristic structure of the U-turn was permanently lost after 2.2 μs and 6.1 μs in simulations of the wild-type and C14+ system, respectively (Figure S11). In both cases, the loss proceeded by simultaneous disruption of the U-turn structure and movement of the A17 base into a position where it would prevent U-turn restoration. Afterwards, the A17 base would form random stacking interactions with the bases normally constituting the U-turn, further degrading the structure of the loop. Note that the A17 base is extensively interacting with RIO within the NSR/RIO complex, thus preventing a similar disruption occurring in the simulations of the NSR system with RIO. Indeed, both wild-type and C14+ structures of the NSR/RIO complex were perfectly maintained for the entire ten-microsecond simulation timescale with no signs of unfolding.

Lastly, note that we regularly observed short reversible disruptions of the U-turn structure even in the course of the one-microsecond simulations (Figure S12). These disruptions were always reversible on the one-microsecond timescale. Representing a small part of the simulation ensemble (under 5%), they did not increase the average NOE violations. Thus, they were not in conflict with the experimental data and might represent a realistic dynamical behavior of the system on the microsecond timescale. Nevertheless, for the purpose of the statistical analyses presented in main text Table 3, we have discarded such parts of the trajectories, in order to not bias the ensemble data. Even though the substates may be realistic, we do not think their population is fully converged on the microsecond timescale, or even on the extended ten-microsecond timescale. We suggest that progression of these minor disruptions, coupled with simultaneous structural interference by the A17 base, is what eventually resulted into permanent loss of the U-turn structure in NSR simulations without the RIO. In simulations, the RIO ligand appears to function as an obstruction against excessive structural changes of the U-turn structure, both by stiffening the loop and by stably binding structural elements that would otherwise interfere in canonical U-turn structure, such as the A17 base.

**Behavior of the U14(O2′)/A16(N7) U-turn signature interaction in NSR simulations**

As noted in the main text, the simulations revealed a minor inaccuracy of the force-field description of the U-turn motif where the U14(O2′)/A16(N7) signature interaction was often replaced by the U14(O2′)/U15(O5′) interaction as an alternative (Figure S3). However, there is strong experimental evidence for the presence of the U14(O2′)/A16(N7) hydrogen bond in the NSR structure. Specifically, there are NOE contacts involving adjacent protons and a ^1^H-NMR signal for the 2’-hydroxyl group of U14, demonstrating that the hydroxyl proton is strongly protected from exchange with the bulk solvent ([19](#_ENREF_19),[20](#_ENREF_20)). The U14 2’-hydroxyl proton furthermore showed a cross hydrogen bond scalar coupling to the A16(N7) nitrogen in a long range HSQC-experiment, thereby confirming directly the presence of this hydrogen bond (Figure S4). In contrast, no cross-hydrogen bond scalar coupling between the U14 2’-OH proton and the U15 phosphate group was observed in long-range ^1^H,^31^P-HSQC experiments ([21](#_ENREF_21)) arguing against a significant population of this alternative H-bonding pattern.

In spite of the experimental data indicating its stable presence, the population of the native U14(O2′)/A16(N7) H-bond amounted to only ~40-50 % in the wild-type MD simulations (Table S1). Fortunately, the rearrangement to the spurious U14(O2′)/U15(O5′) interaction remained entirely localized and did not further propagate into the structure of the U-turn. However, it caused several simulation NOE violations associated with the U14(HO2′) atom (main text Table 2, Supplementary Figure S2). A qualitatively identical behavior for this U-turn signature interaction was also observed in the control simulations of the two additional RNA systems containing the U-turn motif (see main text Table 1). We suggest that the observed change is due to an overstabilization of the hydroxyl-phosphate interactions, which is a well-known problem of the non-bonded parameters of the AMBER force field ([13](#_ENREF_13),[22](#_ENREF_22),[23](#_ENREF_23)).

It has been proposed that the use of alternative phosphate group VdW parameters ([11](#_ENREF_11)) combined with the OPC water model ([12](#_ENREF_12)) can mitigate the problem with hydroxyl-phosphate interactions in MD simulations ([24](#_ENREF_24)). Thus, we tried one such simulation for the NSR/RIO system but no statistically significant improvement has been achieved (Table S1). On the other hand, the signature interaction could be improved by application of the HBfix potential function (Table S1) ([13](#_ENREF_13)). In the present work we have initially used a mild bias of 1 kcal/mol to stabilize the U14(O2′)/A16(N7) interaction in the NSR/RIO system (see main text Table 1). This significantly improved the population of the correct signature interaction in the simulation ensemble to 87% (Table S1). Further improvements of the signature interactions (to 95% and 98%, see Table S1) were achieved when we applied either a 2 kcal/mol stabilizing bias or when we combined the 1 kcal/mol stabilizing bias for the correct interaction with a 1 kcal/mol destabilizing bias for the incorrect interaction (see main text Table 1). Note that the NOE distance violations associated with the U14(HO2′) atom were not completely eliminated by HBfix, but they were significantly fewer and smaller (Figure S2). Importantly, there were no new NOE violations or other structural issues introduced by the use of the HBfix potential function.

**The presence of RIO attenuates the RNA dynamics in simulations with bound ligand.**

The fluctuations of the RNA bulge involving nucleotides C6, U7 and U8 were dependent on the presence of the ligand. Specifically, in simulations with RIO, the C6 base was stably stacked with the A17 base which, in turn, was stacked with RIO in agreement with previous NMR-data ([19](#_ENREF_19)). In absence of the ligand, both the C6 and A17 nucleotides became more flexible. On the other hand, the U7 and U8 bases were extensively fluctuating in all simulations, regardless of the ligand’s presence (main text Figure 2 and Supplementary Figure S13). This result is in complete agreement with the earlier NMR experiments where high ^13^C-HetNOE values were observed for both the H6-C6 and H1’-C1’ moieties in U7 and U8, whereas for residue C6 only the H1’C1’-^13^C-HetNOE value was increased ([25](#_ENREF_25)). Thus, in the C6 nucleotide the ribose is flexible but the base moiety is conformationally restricted due to its stacking interaction with the base of A17. The presence of the ligand also strongly dampened conformational fluctuations in the U-turn loop (main text Figure 2). Again, this agrees very well with the NMR observations that showed base and sugar HetNOE values for all loop nucleotides to be similar to those of the stable stem nucleotides in the RIO bound state ([19](#_ENREF_19),[20](#_ENREF_20)).

Comparison with simulation of free RIO indicates that the ligand dynamics is greatly suppressed upon binding to the NSR (Figure S14) and all the native RNA/RIO intermolecular H-bonds were maintained with high occupancy in the simulations (Table S2). We observed the formation of two additional H-bonds in our simulations that are absent in the NMR ensemble. However, their heavy atom distances were as close as 3.7 Å in the NMR ensemble, and nearly satisfied our H-bond criterion (see main text Methods). Thus, the formation of these two new H-bonds in our simulations did not represent a significant structural change.

Lastly, note that the simulation ion sites in the NSR system were also affected by the presence of the RIO ligand. Specifically, in the systems without RIO, the K^+^ ions would fill the regions normally occupied by the positively charged amino groups of the ribostamycin (the RIO has a formal charge of +4). Thus, in the simulations without RIO, there were additional K^+^ ion binding sites observed inside the RIO binding pocket. However, all ion sites (including the one at the U14(O4) atom; see main text) observed in the simulations with RIO (main text Figure 5) were also observed in simulations without the ligand (Figure S15).

**QM calculations of the small model systems.** The large-scale QM/MM optimizations of the entire NSR reported in the main text were further complemented by QM calculations of *small model systems* containing uracil and protonated cytosine monomers with N3-H3/OP2 H-bonding to a sugar-phosphate backbone segment (Figure S16) using the same QM methods as in the QM/MM calculation of the fully solvated NSR (see main text). The goal of these calculations was to dissect the influence of the full structural context of the NSR upon the studied interactions. Please see Ref. ([26](#_ENREF_26)) for further examples and justifications of similar calculations.

The results showed that the N3-H3 bond elongation (stretching) was even larger (+0.032 Å and +0.038 Å for uracil and cytosine, respectively; see Table S3) in QM optimizations of the small model systems than in the complete and fully solvate NSR (+0.017 Å and +0.030 Å for uracil and cytosine, respectively; see main text Table 4). This indicates that the H-bonds are slightly weakened (screened) by the full structural context of the NSR compared to an idealized case in which they would be isolated from all the other interactions.

We have also further explored the role of the K^+^ ion in the wild-type NSR by computing the interaction energy between the bases and the sugar-phosphate backbone fragment in the small model systems (Figure S16) using DFT-D3 (B3LYP-D3/def2-TZVP level) and the COSMO solvent approximation (see the Supplementary Methods). The interaction energy was -5.7 and -16.5 kcal/mol for the wild-type and C14+ systems, respectively. However, when including the K^+^ ion as part of the wild-type system, at the position suggested by MD, the interaction energy became -20.2 kcal/mol. Note that in the model including the K^+^ ion (Figure S16), the ion and the U14 base were taken as a unified fragment for the calculation of the interaction energy to mirror the charge-distribution found in the C14+ system (negative phosphate and positive base). From this charge distribution the reported interaction energies should not be understood in terms of H-bonding alone, but as having a significant ionic bonding component as well. While the C14+ system provides the ionic bonding by design, the U14(N3)/A17(OP2) interaction needs the help from a cation, which is why we suggest to think about it in terms of an ion-assisted (H-bonding) binding process. However, note that this local interaction is only one of the components comprising the whole structure of the NSR system. A general rule, which is not always respected in literature, is that no extrapolations should be made about the thermodynamic stability of the whole system based on an analysis of a single interaction in QM calculations ([27](#_ENREF_27)).

**The QM/MM calculations disfavor the bifurcated variant of the U-turn signature interaction in the C14+ system.**

Earlier NMR data, including the C14+ amino group chemical shifts and the absence of cross-hydrogen bond scalar couplings between the N4 amino protons and the A17 phosphate group, tentatively suggested that there is no interaction between them in the C14+ mutant system ([28](#_ENREF_28)). Nevertheless, in principle, only a miniscule structural change would be required for the formation of a bifurcated C14+(N4,N3)/A17(OP2) H-bond (Figure S17) instead of the native C14+(N3)/A17(OP2) H-bond.

Indeed, our MD simulations often showed a suitable H-bonding geometry (see Methods) for the bifurcated H-bond (Figure S17), which had 77% and 60% populations in the C14+ simulations with and without RIO, respectively. This did not compromise the native signature interaction via the N3 atom that was populated close to 100% in all simulations. Still, the MD simulations indicated a possibility of the C14+(N4,N3)/A17(OP2) H-bond formation in the C14+ system as an auxiliary interaction. Since the current experimental evidence cannot completely either rule out or confirm the existence of this bifurcated H-bond in the C14+ NSR system, we decided to further explore its stability by performing a QM/MM optimization of a simulation snapshot containing the bifurcated C14+(N4,N3)/A17(OP2) H-bond, using identical methodology as for the other QM/MM calculations. During the optimization the interaction with the N4 atom was lost and an exclusive C14+(N3)/A17(OP2) hydrogen bonding interaction was established (Table S4). Somewhat surprisingly, a similar outcome occurred also in optimizations using the MM force field, even when using SHAKE to constrain the X-H bonds. The bifurcated H-bond geometry was not entirely lost during the MM optimizations, however, its H-bond angle for the N4/OP2 interaction was lowered and was very close to the cut-off value (see the Methods). Thus, the C14+(N4,N3)/A17(OP2) H-bond bifurcation seen in the MD simulation trajectory could be a consequence of thermal sampling (the optimizations correspond to a 0 K potential energy surface). Still, the force field might be incorrectly supporting its excessive occurrence in the MD simulations since there is no parameter in the force field addressing the directionality of hydrogen bonds.

**A neutral C14 base combined with a protonated phosphate is an energetically unfavorable state.** In one of the QM calculations, we explored the possibility of a neutral C14+ system where the A17 phosphate would be protonated and the cytosine 14 in its canonical, unprotonated form, thus satisfying the same signature H-bond interaction. The large pK­a^­­­^ ­difference (4.2 and 2.0 for the cytosine N3 atom and an RNA phosphate, respectively) ([29](#_ENREF_29)) would make such a protonation state very rare in solution in an isolated system. However, it was shown that even rare protonation states can influence RNA biochemistry  ([30](#_ENREF_30)) Therefore, we have performed a relaxed potential energy surface (PES) scan for the proton transfer from C14+ to the neighboring phosphate group in a small model system (Figure S18) to evaluate the possibility of a neutral C14 system instead of the zwitterionic one. The C14+ hydrogen was displaced along the N3…OP2 vector in several steps as shown in Figure S18. On each point of the PES a restrained optimization at the COSMO-B3LYP-D3/def2-TZVP level of theory was performed using the same restraints as described in Figure S16 combined with additional constraints that keep the three atoms involved in the proton transfer (N3…H…OP2) frozen. Finally, single point energy refinements on the optimized structures were performed at the DLPNO-CCSD(T)/def2-TZVP level of theory with the C-PCM solvation model using the ORCA program suite  ([31](#_ENREF_31),[32](#_ENREF_32)). According to the calculations, the proton transfer energy barrier height is 11.1 kcal/mol and the neutral system (right side of Figure S18) is 6.9 kcal/mol higher in energy than the zwitterionic system. The forward activation energy barrier is relatively high, making the protonated phosphate highly unfavorable, but in principle accessible at room temperature.

# Supplementary Information References

1. Krepl, M., Cléry, A., Blatter, M., Allain, F.H.T. and Sponer, J. (2016) Synergy between NMR Measurements and MD Simulations of Protein/RNA Complexes: Application to the RRMs, the Most Common RNA Recognition Motifs. *Nucleic Acids Res.*, **44**, 6452-6470.

2. Salomon-Ferrer, R., Götz, A.W., Poole, D., Le Grand, S. and Walker, R.C. (2013) Routine Microsecond Molecular Dynamics Simulations with AMBER on GPUs. 2. Explicit Solvent Particle Mesh Ewald. *J. Chem. Theory Comput.*, **9**, 3878-3888.

3. Le Grand, S., Götz, A.W. and Walker, R.C. (2013) SPFP: Speed without Compromise—A Mixed Precision Model for GPU Accelerated Molecular Dynamics Simulations. *Comput. Phys. Commun.*, **184**, 374-380.

4. Pitera, J.W. and Chodera, J.D. (2012) On the Use of Experimental Observations to Bias Simulated Ensembles. *J. Chem. Theory Comput.*, **8**, 3445-3451.

5. Darden, T., York, D. and Pedersen, L. (1993) Particle Mesh Ewald - An N.Log(N) Method for Ewald Sums in Large Systems. *J. Chem. Phys.*, **98**, e10089.

6. Essmann, U., Perera, L., Berkowitz, M.L., Darden, T., Lee, H. and Pedersen, L.G. (1995) A Smooth Particle Mesh Ewald Method. *J. Chem. Phys.*, **103**, 8577-8593.

7. Ryckaert, J.P., Ciccotti, G. and Berendsen, H.J.C. (1977) Numerical-Integration of Cartesian Equations of Motion of a System with Constraints - Molecular-Dynamics of N-Alkanes. *J. Comput. Phys.*, **23**, 327-341.

8. Hopkins, C.W., Le Grand, S., Walker, R.C. and Roitberg, A.E. (2015) Long-Time-Step Molecular Dynamics through Hydrogen Mass Repartitioning. *J. Chem. Theory Comput.*, **11**, 1864-1874.

9. Berendsen, H.J.C., Postma, J.P.M., Vangunsteren, W.F., Dinola, A. and Haak, J.R. (1984) Molecular-Dynamics with Coupling to an External Bath. *J. Chem. Phys.*, **81**, 3684-3690.

10. D.A. Case, R.M.B., W. Botello-Smith, D.S. Cerutti, T.E. Cheatham, III, T.A. Darden, R.E. Duke, T.J. Giese, H. Gohlke, A.W. Goetz, N. Homeyer, S. Izadi, P. Janowski, J. Kaus, A. Kovalenko, T.S. Lee, S. LeGrand, P. Li, C. Lin, T. Luchko, R. Luo, B. Madej, D. Mermelstein, K.M. Merz, G. Monard, H. Nguyen, H.T. Nguyen, I. Omelyan, A. Onufriev, D.R. Roe, A. Roitberg, C. Sagui, C.L. Simmerling, J. Swails, R.C. Walker, J. Wang, R.M. Wolf, X. Wu, L. Xiao, D.M. York, and P.A. Kollman. (2016). University of California, San Francisco.

11. Steinbrecher, T., Latzer, J. and Case, D.A. (2012) Revised AMBER Parameters for Bioorganic Phosphates. *J. Chem. Theory Comput.*, **8**, 4405-4412.

12. Izadi, S., Anandakrishnan, R. and Onufriev, A.V. (2014) Building Water Models: A Different Approach. *J. Phys. Chem. Lett.*, **5**, 3863-3871.

13. Kuhrova, P., Best, R., Bottaro, S., Bussi, G., Sponer, J., Otyepka, M. and Banas, P. (2016) Computer Folding of RNA Tetraloops: Identification of Key Force Field Deficiencies. *J. Chem. Theory Comput.*, **12**, 4534–4548.

14. Šponer, J., Krepl, M., Banáš, P., Kührová, P., Zgarbová, M., Jurečka, P., Havrila, M. and Otyepka, M. (2017) How to Understand Atomistic Molecular Dynamics Simulations of RNA and Protein–RNA Complexes? *Wiley Interdiscip. Rev.: RNA*, **8**, e1405.

15. Klamt, A. and Schuurmann, G. (1993) Cosmo - A New Approach to Dielectric Screening in Solvents with Explicit Expressions for the Screening Energy and Its Gradient. *J. Chem. Soc., Perkin Trans. 2*, **2**, 799-805.

16. Becke, A.D. (1993) Density-functional Thermochemistry. III. The Role of Exact Exchange. *J. Chem. Phys.*, **98**, 5648-5652.

17. Grimme, S., Ehrlich, S. and Goerigk, L. (2011) Effect of the Damping Function in Dispersion Corrected Density Functional Theory. *J. Comput. Chem.*, **32**, 1456-1465.

18. Stephens, P.J., Devlin, F.J., Chabalowski, C.F. and Frisch, M.J. (1994) Ab Initio Calculation of Vibrational Absorption and Circular Dichroism Spectra Using Density Functional Force Fields. *J. Phys. Chem.*, **98**, 11623-11627.

19. Duchardt-Ferner, E., Gottstein-Schmidtke, S.R., Weigand, J.E., Ohlenschläger, O., Wurm, J.-P., Hammann, C., Suess, B. and Wöhnert, J. (2016) What a Difference an OH Makes: Conformational Dynamics as the Basis for the Ligand Specificity of the Neomycin-Sensing Riboswitch. *Angew. Chem. Int. Ed.*, **55**, 1527-1530.

20. Weigand, J.E., Schmidtke, S.R., Will, T.J., Duchardt-Ferner, E., Hammann, C., Wöhnert, J. and Suess, B. (2011) Mechanistic Insights into an Engineered Riboswitch: A Switching Element which Confers Riboswitch Activity. *Nucleic Acids Res.*, **39**, 3363-3372.

21. Duchardt-Ferner, E., Ferner, J. and Wöhnert, J. (2011) Rapid Identification of Noncanonical RNA Structure Elements by Direct Detection of OH...O.P, NH...O.P, and NH2...O.P Hydrogen Bonds in Solution NMR Spectroscopy. *Angew. Chem. Int. Ed.*, **50**, 7927-7930.

22. Mlynsky, V., Kuhrova, P., Zgarbova, M., Jurecka, P., Walter, N.G., Otyepka, M., Sponer, J. and Banas, P. (2015) Reactive Conformation of the Active Site in the Hairpin Ribozyme Achieved by Molecular Dynamics Simulations with epsilon/zeta Force Field Reparametrizations. *J. Phys. Chem. B*, **119**, 4220-4229.

23. Condon, D.E., Kennedy, S.D., Mort, B.C., Kierzek, R., Yildirim, I. and Turner, D.H. (2015) Stacking in RNA: NMR of Four Tetramers Benchmark Molecular Dynamics. *J. Chem. Theory Comput.*, **11**, 2729-2742.

24. Bergonzo, C. and Cheatham, T.E. (2015) Improved Force Field Parameters Lead to a Better Description of RNA Structure. *J. Chem. Theory Comput.*, **11**, 3969-3972.

25. Duchardt-Ferner, E., Weigand, J.E., Ohlenschläger, O., Schmidtke, S.R., Suess, B. and Wöhnert, J. (2010) Highly Modular Structure and Ligand Binding by Conformational Capture in a Minimalistic Riboswitch. *Angew. Chem. Int. Ed.*, **49**, 6216-6219.

26. Zgarbová, M., Jurečka, P., Banáš, P., Otyepka, M., Šponer, J.E., Leontis, N.B., Zirbel, C.L. and Šponer, J. (2011) Noncanonical Hydrogen Bonding in Nucleic Acids. Benchmark Evaluation of Key Base–Phosphate Interactions in Folded RNA Molecules Using Quantum-Chemical Calculations and Molecular Dynamics Simulations. *J. Phys. Chem. A*, **115**, 11277-11292.

27. Šponer, J., Šponer, J.E., Mládek, A., Banáš, P., Jurečka, P. and Otyepka, M. (2013) How to Understand Quantum Chemical Computations on DNA and RNA Systems? A Practical Guide for Non-specialists. *Methods*, **64**, 3-11.

28. Gottstein-Schmidtke, S.R., Duchardt-Ferner, E., Groher, F., Weigand, J.E., Gottstein, D., Suess, B. and Wöhnert, J. (2014) Building a Stable RNA U-turn with a Protonated Cytidine. *RNA*, **20**, 1163-1172.

29. Hiller, D.A. and Strobel, S.A. (2011) The Chemical Versatility of RNA. *Philos. Trans. Royal Soc. B*, **366**, 2929-2935.

30. Kimsey, I.J., Petzold, K., Sathyamoorthy, B., Stein, Z.W. and Al-Hashimi, H.M. (2015) Visualizing Transient Watson-Crick-like Mispairs in DNA and RNA Duplexes. *Nature*, **519**, 315-320.

31. Riplinger, C. and Neese, F. (2013) An Efficient and Near Linear Scaling Pair Natural Orbital Based Local Coupled Cluster Method. *J. Chem. Phys.*, **138**, e034106.

32. Barone, V. and Cossi, M. (1998) Quantum Calculation of Molecular Energies and Energy Gradients in Solution by a Conductor Solvent Model. *J. Phys. Chem. A*, **102**, 1995-2001.

33. Castrignanò, T., Chillemi, G., Varani, G. and Desideri, A. (2002) Molecular Dynamics Simulation of the RNA Complex of a Double-Stranded RNA-Binding Domain Reveals Dynamic Features of the Intermolecular Interface and Its Hydration. *Biophys. J.*, **83**, 3542-3552.

34. Krepl, M., Blatter, M., Cléry, A., Damberger, F.F., Allain, F.H.T. and Sponer, J. (2017) Structural Study of the Fox-1 RRM Protein Hydration Reveals a Role for Key Water Molecules in RRM-RNA Recognition *Nucleic Acids Res.*, **45**, 8046-8063.

35. Mayer, I. (2006) Energy Partitioning Schemes. *Phys. Chem. Chem. Phys.*, **8**, 4630-4646.

# Supplementary Information Tables

Table S1. Populations of the U14(O2′)/A16(N7) H-bond in MD simulations of the Neomycin sensing riboswitch.

| **system^a^** | **U14(O2′)/A16(N7)** |
| --- | --- |
| **2n0j_wt** | 51% |
| **2n0j_RIO_wt** | 38% |
| **2n0j_RIO_wt_CaseP_OPC** | 47% |
| **2n0j_RIO_wt_HBfix_A** | 81% |
| **2n0j_RIO_wt_HBfix_B** | 95% |
| **2n0j_RIO_wt_HBfix_C** | 98% |
| **2n0j_RIO_wt_Na** | 33% |
| **2n0j_C14+** | 67% |
| **2n0j_RIO_C14+** | 23% |

^a^For systems where multiple parallel simulations were conducted (see main text Table 1), the H-bond populations were computed for the combined one-microsecond simulation ensembles (see the main text).

Table S2. List of the NSR/RIO intermolecular H-bond occupancies in the NMR ensemble and selected simulation ensembles of the NSR.^a^

| **H-bond** | **NMR** | **2n0j_RIO_wt** | **2n0j_RIO_wt_HBfix_B** | **2n0j_RIO_**  **wt_Na** | **2n0j_RIO_**  **C14+** |
| --- | --- | --- | --- | --- | --- |
| G5(N7)/RIO(N21) | 0% | 90% | 90% | 87% | 91% |
| G9(OP1)/RIO(N) | 100% | 85% | 84% | 57%^b^ | 95% |
| U10(O4)/RIO(N) | 85% | 95% | 95% | 96% | 97% |
| G19(N7)/RIO(N12) | 80% | 97% | 97% | 97% | 98% |
| G19(O6)/RIO(O13) | 100% | 85% | 85% | 86% | 84% |
| G19(OP2)/RIO(O35) | 100% | 82% | 86% | 88% | 89% |
| G20(N7)/RIO(N12) | 0% | 96% | 96% | 97% | 97% |
| G20(O6)/RIO(N23) | 100% | 98% | 98% | 98% | 98% |
| U21(O4)/RIO(N23) | 95% | 92% | 92% | 92% | 93% |

^a^The reported occupancies of the H-bonds are almost always below 100%, partly due to use of arbitrary interatomic distance and angle values as cut-offs for definition of the H-bonding geometry (see the Methods). This definition discards some thermal fluctuation configurations as not H-bonded, accounting for as much as 2~3% of the simulation snapshots. Another factor is reversible water-mediation of the solvent exposed H-bonds which can occur by temporary insertion of water molecules between the H-bond constituting atoms (Figure S19). Such dynamical water insertion is very typical for H-bonds constituted by charged atomic groups  ([33](#_ENREF_33),[34](#_ENREF_34)). ^b^The H-bond occupancy was affected by a Na^+^ specific ion site forming in the simulations (see Figure S20).

Table S3. Geometry parameters of the U14(N3)/A17(OP2) and C14+(N3)/A17(OP2) H-bonds in the optimized structures of the small model systems.

|  | **N3-H3 bond and interatomic distances (in Å)** | | |
| --- | --- | --- | --- |
|  | **N3-H3** | **H3/OP2** | **N3/OP2** |
| **method^a^** | **QM** | **QM** | **QM** |
| **models^b^** |  |  |  |
| model_wt | 1.049 | 1.67 | 2.71 |
| model_wt_K^+^ | 1.048 | 1.68 | 2.71 |
| model_C14+ | 1.055 | 1.65 | 2.63 |

^a^Method used to perform the optimization: MM – molecular mechanics (bsc0_χOL3_), QM – TPSS-D3. ^b^See Figure S16 for visualization of the small model systems.

Table S4. The geometry parameters of the C14+(N4,N3)/A17(OP2) bifurcated H-bond in the QM/MM and MM optimization calculations.

|  |  | **Interatomic distance [Å] and angle [°]** | | | |
| --- | --- | --- | --- | --- | --- |
| **method^a^** | **initial** |  | **MM** | **MM_SHAKE_** | **QM/MM** |
| **N3-H3** | 1.018 | 🡪 | 1.012 | 1.009 | 1.042 |
| **H3-OP2** | 2.04 | 🡪 | 1.84 | 1.84 | 1.76 |
| **N3-OP2** | 2.91 | 🡪 | 2.78 | 2.77 | 2.74 |
| **N3/H3/OP2 (angle)** | 141.78 | 🡪 | 152.01 | 150.87 | 154.60 |
| **N4-H41** | 1.005 | 🡪 | 1.015 | 1.010 | 1.020 |
| **H41-OP2** | 2.00 | 🡪 | 2.41 | 2.25 | 2.63 |
| **N4-OP2** | 2.88 | 🡪 | 3.11 | 3.02 | 3.19 |
| **N4/H41/OP2 (angle)** | 145.15 | 🡪 | 125.66 | 131.65 | 114.39 |

^a^Method used to perform the optimization: **MM** – molecular mechanics (bsc0_χOL3_); **MM_SHAKE_** – molecular mechanics (bsc0_χOL3_) with increased bond force constant (approximating SHAKE restraints) applied to the X-H bonds; QM/MM – TPSS-D3 in the QM part of the system, bsc0_χOL3_ in the MM part of the system.

# Supplementary Information Figures


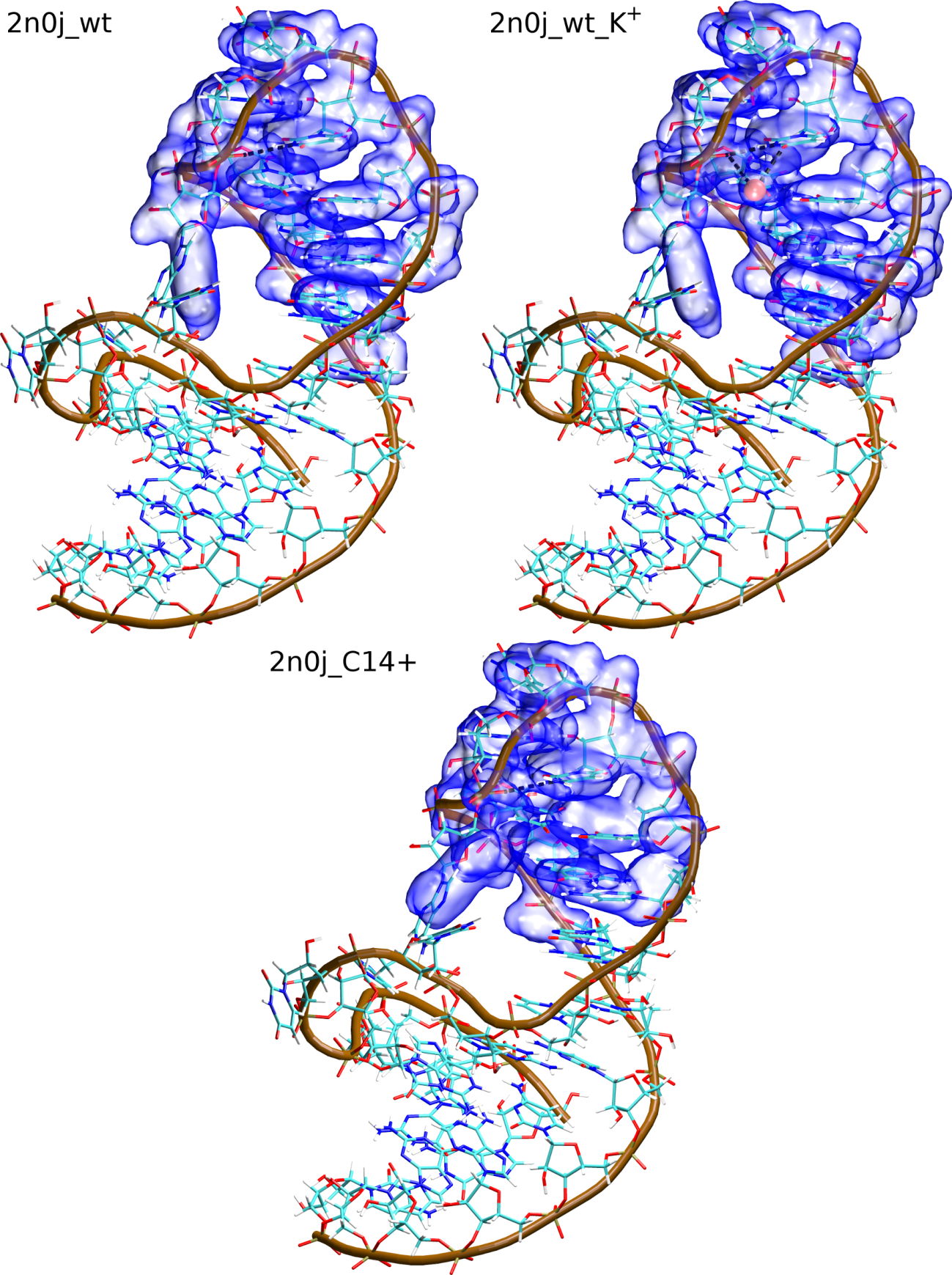


Figure S1. Definition of QM regions in QM/MM computations. The blue highlight indicates parts of the NSR system included in the QM region of the individual QM/MM calculations.


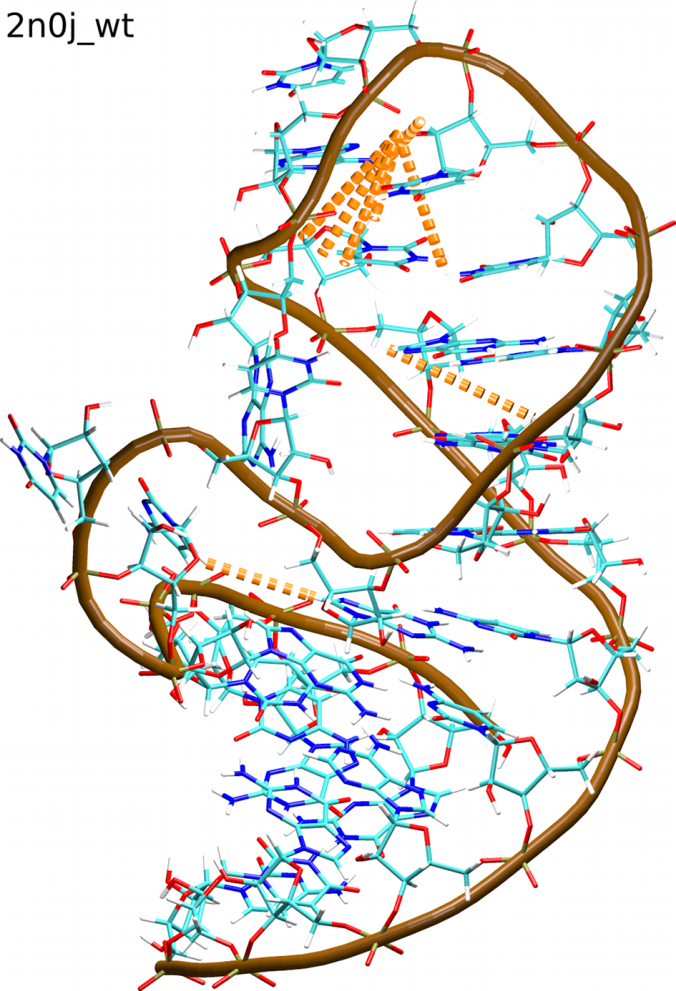

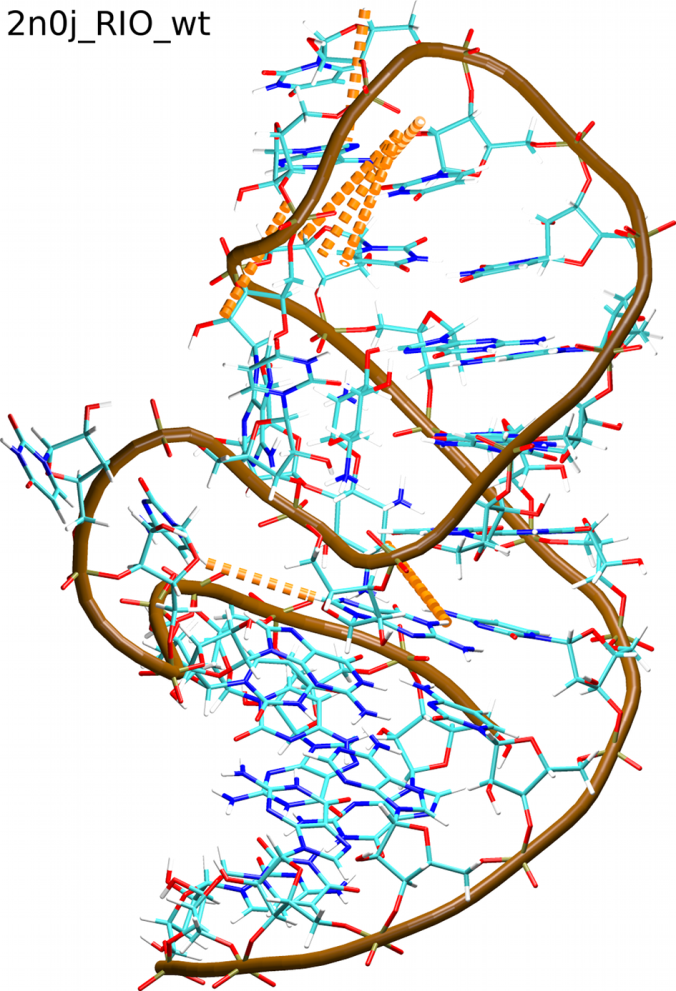

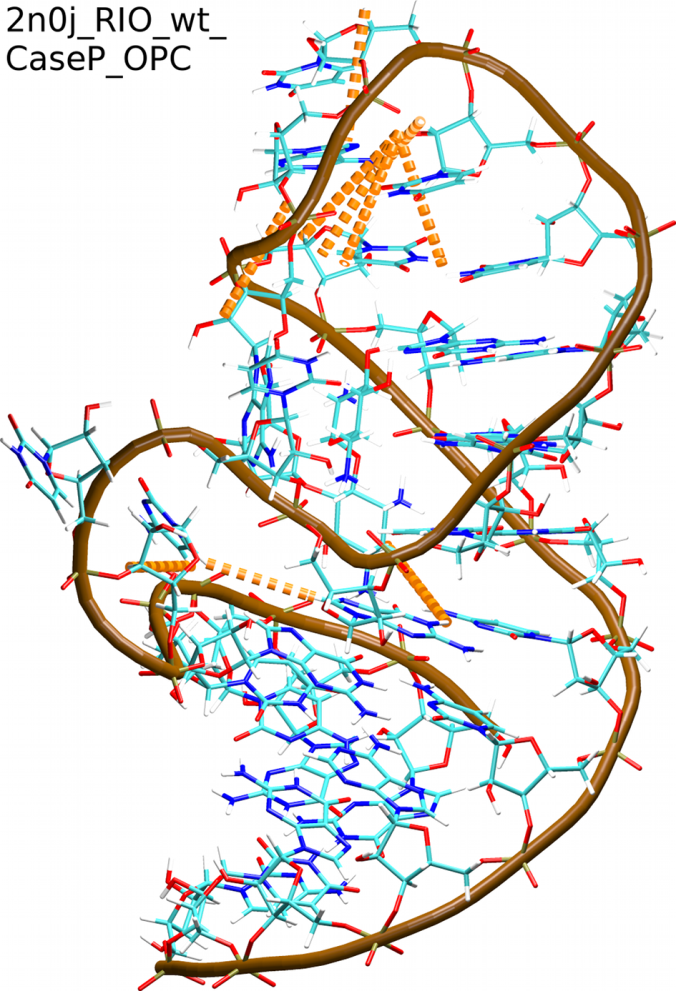

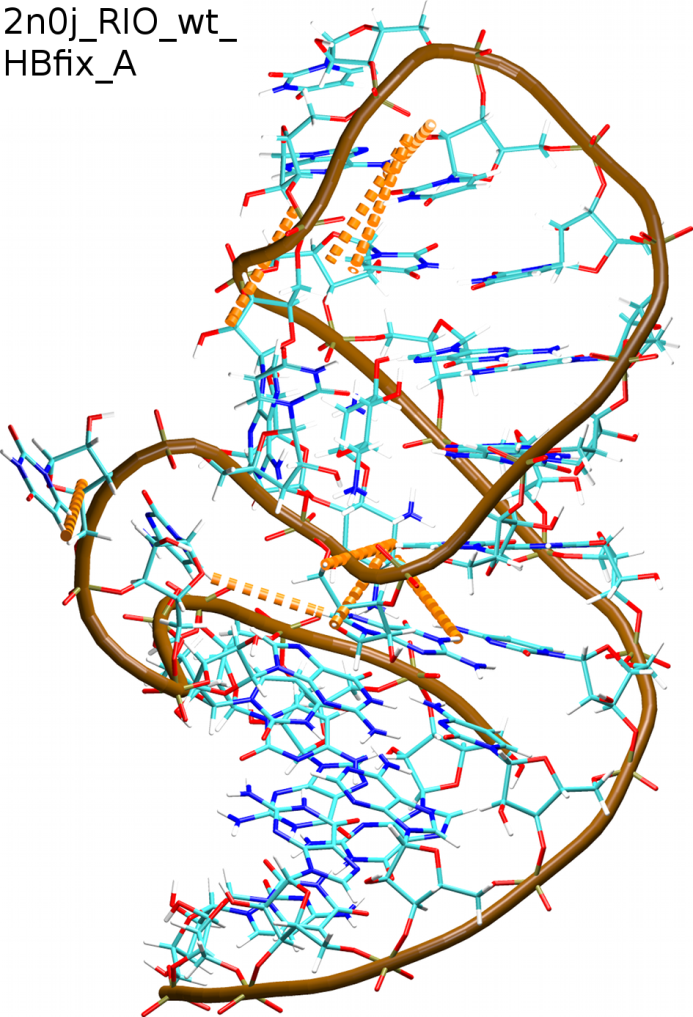


Figure S2. NOE violations. The dashed orange lines indicate violated hydrogen-hydrogen NOE distances (over 0.3 Å) in the individual simulation ensembles of the NSR system.


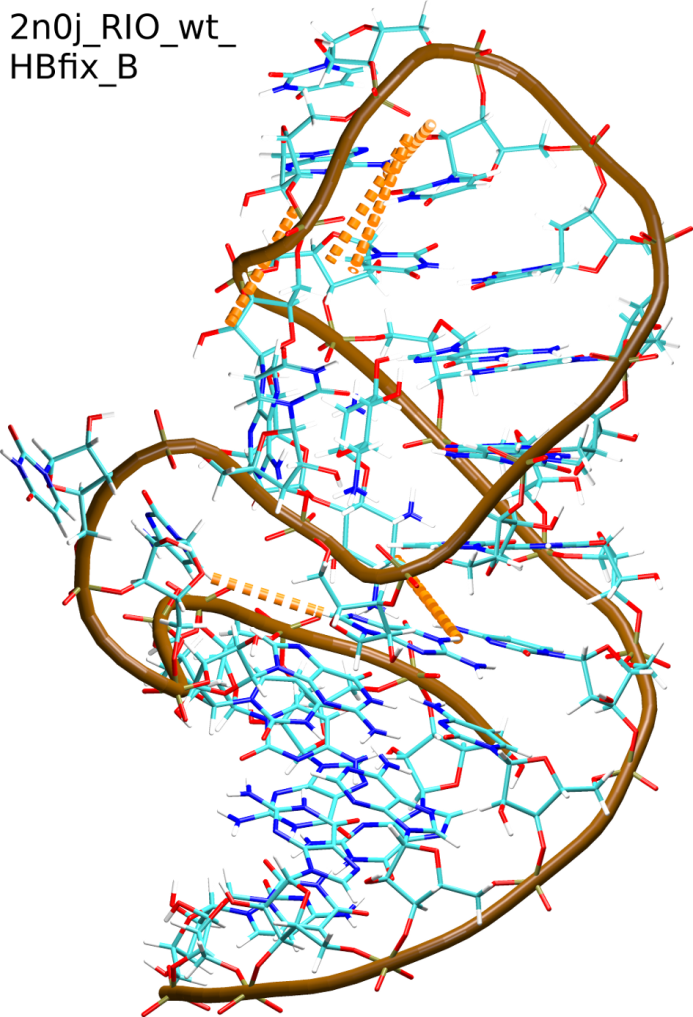

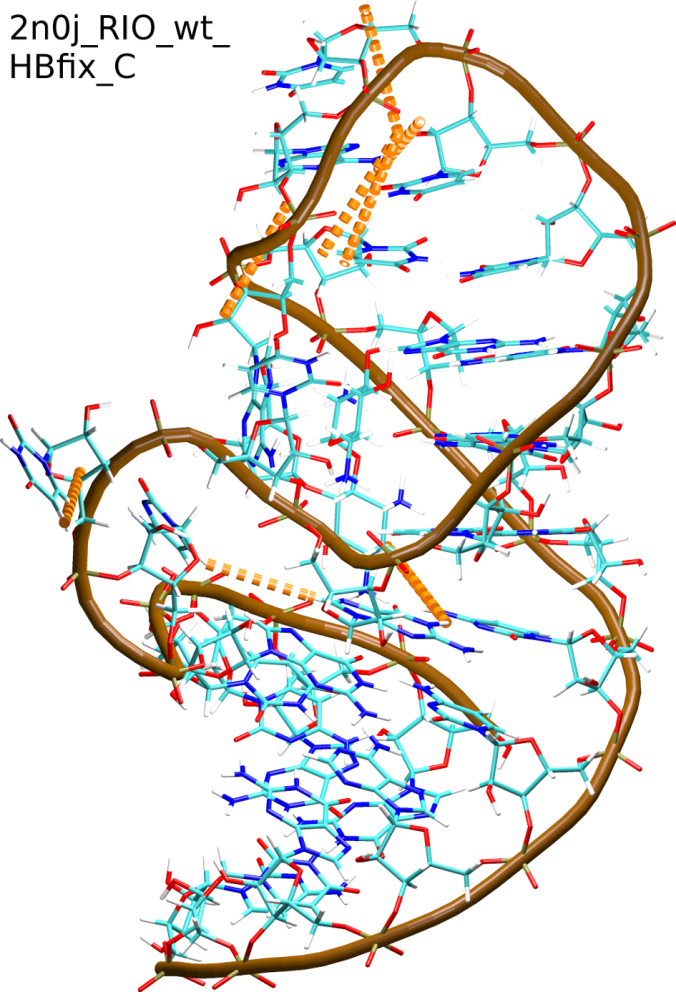

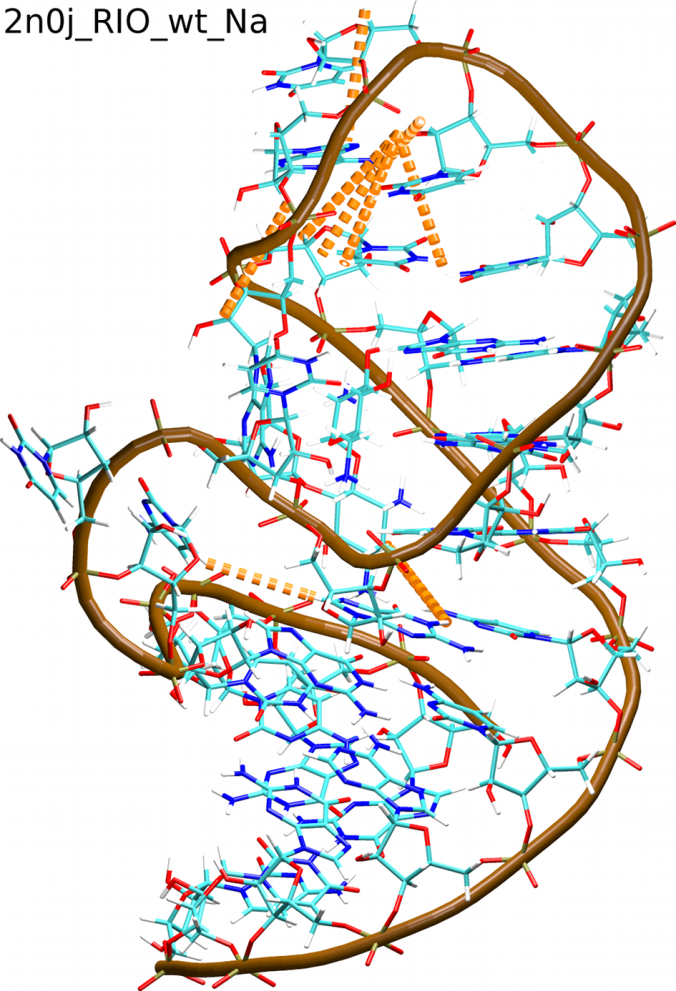

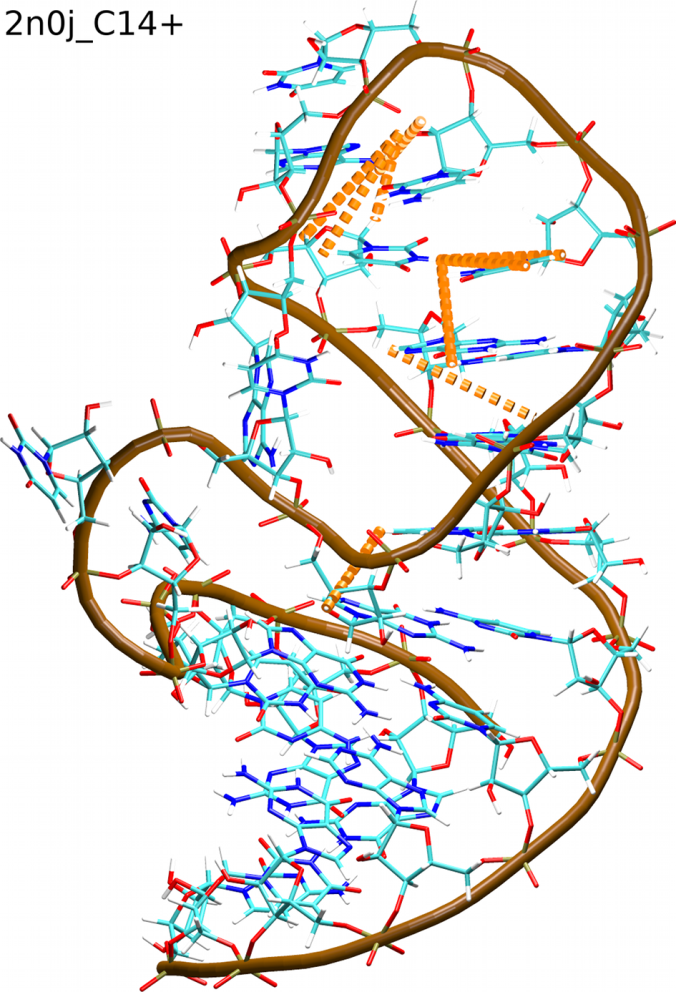


Figure S2. (continuation) NOE violations. The dashed orange lines indicate violated hydrogen-hydrogen NOE distances (over 0.3 Å) in the individual simulation ensembles of the NSR system.


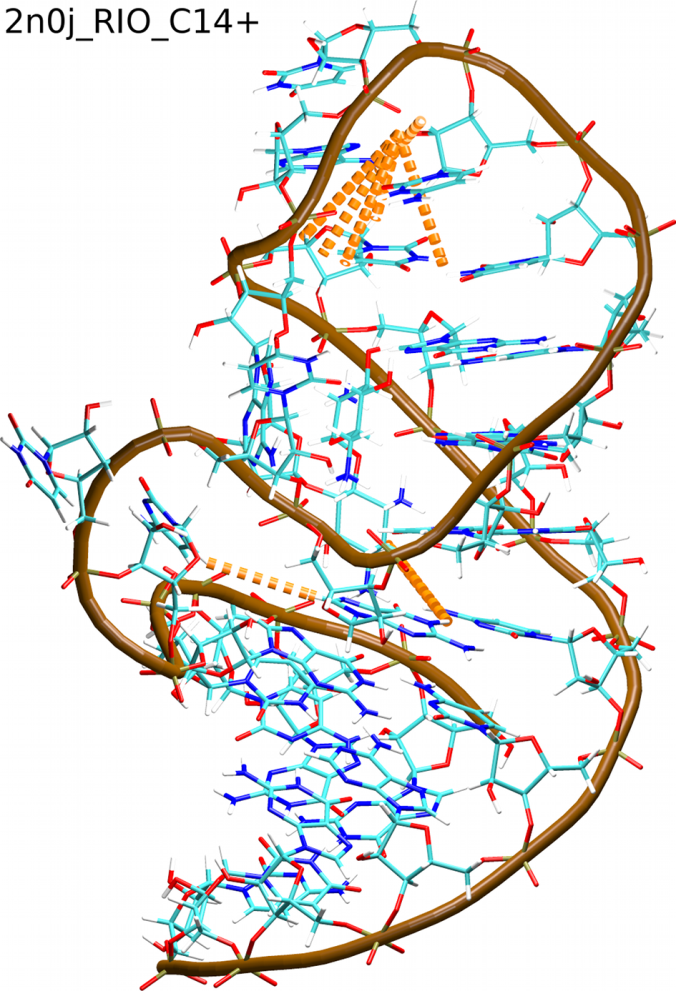


Figure S2. (continuation) NOE violations. The dashed orange lines indicate violated hydrogen-hydrogen NOE distances (over 0.3 Å) in the individual simulation ensembles of the NSR system.


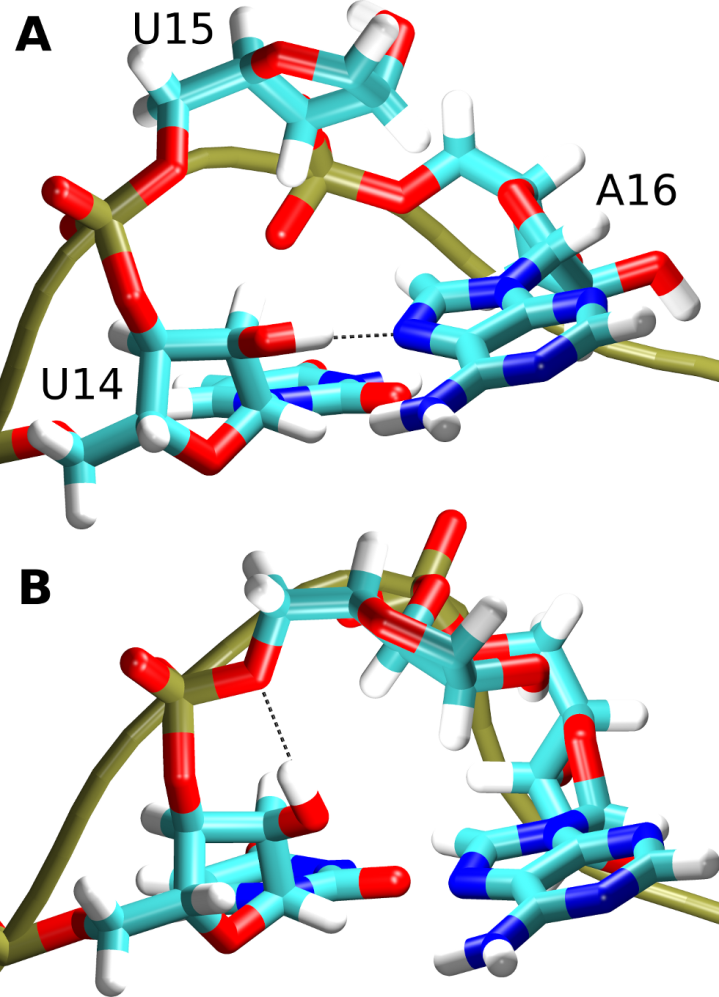


Figure S3. Behavior of the signature H-bond in MD simulations. (**A**) The U14(O2′)/A16(N7) signature H-bond interaction often changes into (**B**) spurious U14(O2′)/U15(O5′) interaction in MD simulations of the NSR system. The dashed black lines indicate H-bonds. For clarity, the U15 base is not shown.


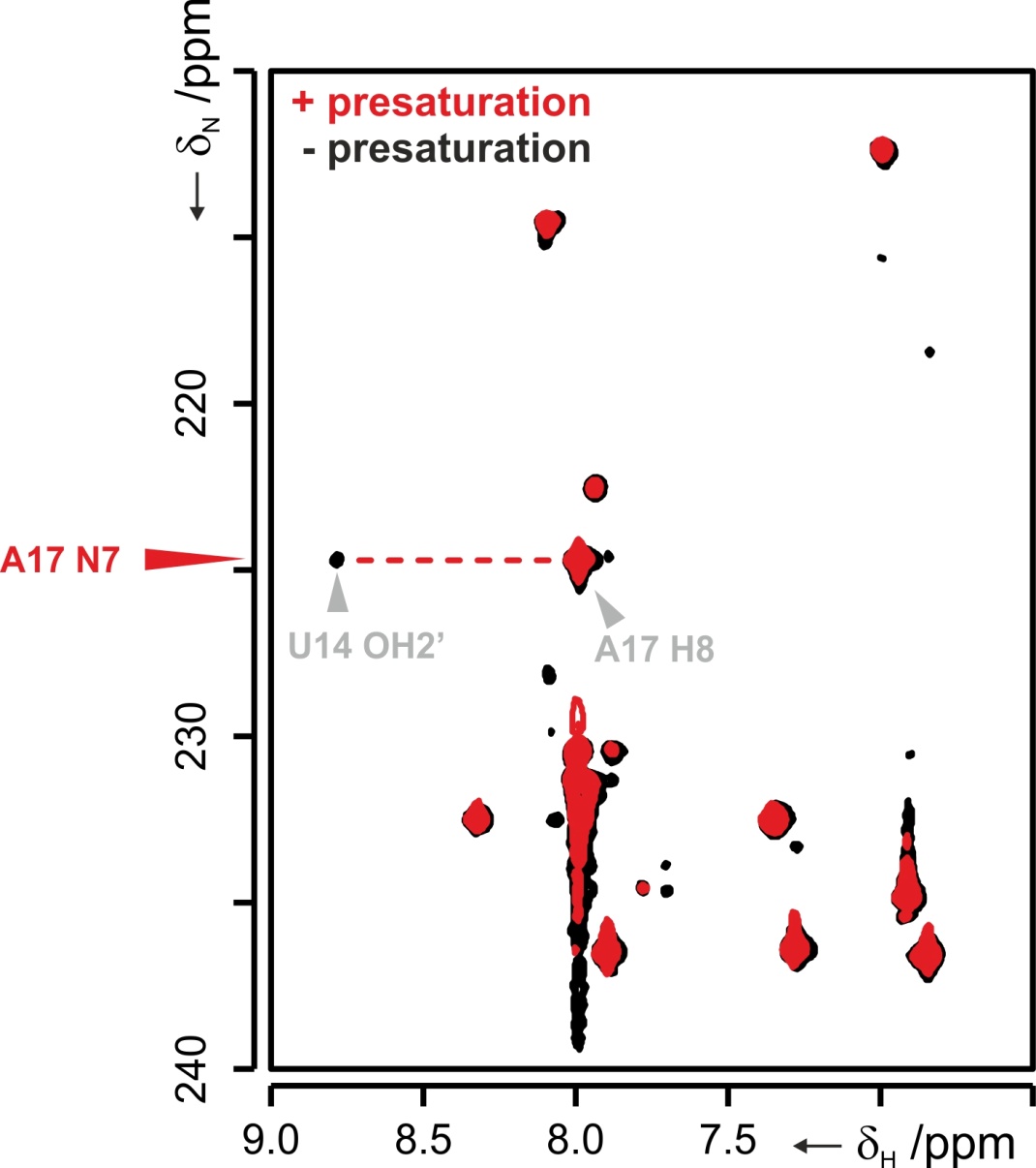


Figure S4. Presence of a hydrogen bond between the U14 2‘-OH group and A17(N7) atom, as detected by long range ^15^N-HSQC experiments. An overlay between a spectrum employing selective presaturation of the solvent water (red) and a spectrum without presaturation (black) is shown. The correlation signal between the U14 2’-OH proton and the A17(N7) nitrogen atom showing directly the presence of a hydrogen bond between these two moieties is highlighted and connected to the A17 H8-N7 intrabase correlation (dashed red line). The cross peak involving U14 2’-OH is missing in the spectrum recorded with presaturation due to exchange of 2’-OH proton with the solvent water, while the remaining cross peaks stemming from correlations involving non-exchangeable H2 and H8 atoms are retained. Spectra were recorded at 10 °C on a Bruker 600 MHz spectrometer with an H-N transfer delay of 33.3 ms.


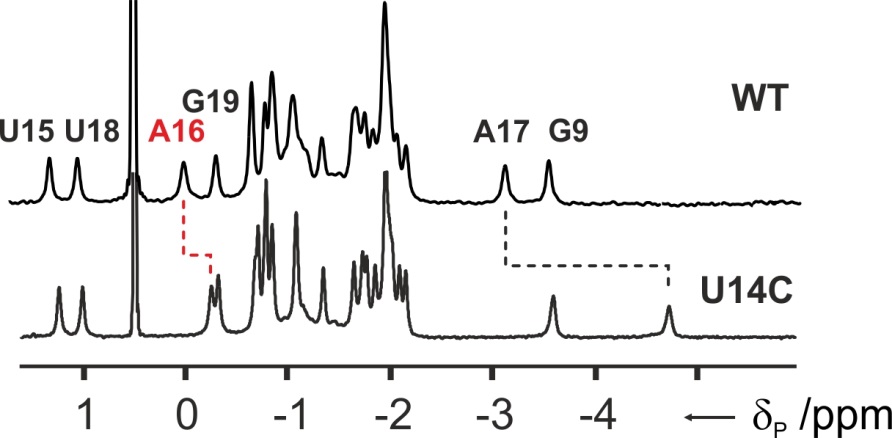


Figure S5. 1D-^31^P spectra of the wild-type neomycin riboswitch (top) and of the U14C+ mutant (bottom). Resolved phosphate resonances of the loop and the ligand-binding site are assigned. Labels correspond to the 5’-phosphate group of the respective nucleotide. The resonances of phosphate groups experiencing the most significant shifts between the wild-type and the U14C mutant are connected by dashed lines. The resonance of the 5’-phosphate group of A16 is highlighted in red. Its non-bridging oxygen OP2 forms a cation-π interaction with the aromatic base of nucleotide 14. The very large chemical shift difference observed for the 5’-phosphate group of A17 in the two RNAs is due its role as the acceptor group in the hydrogen bond with the imino group of either U14 or C14+. Spectra were recorded on a Bruker 600 MHz spectrometer at 25 °C.


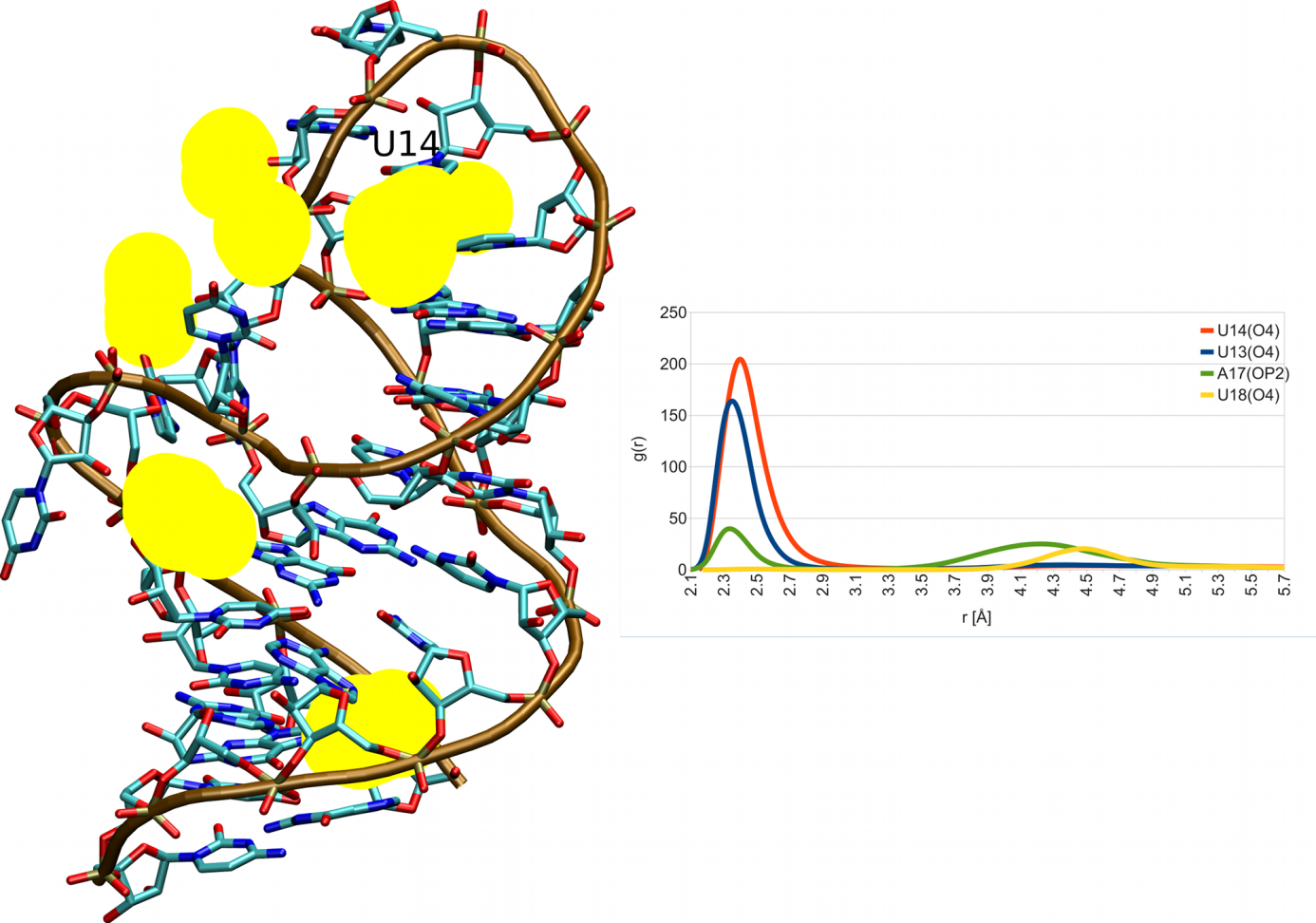


Figure S6. Ion binding in MD simulations with NaCl. The yellow spheres represent the highest density points for the Na^+^ ions in simulations of the wild-type NSR system with RIO (the ligand is not displayed). The graph shows normalized radial distribution functions of Na^+^ ions pair-wise distances for the individual coordinating atoms of the U14(O4) ion-binding site in the simulations.


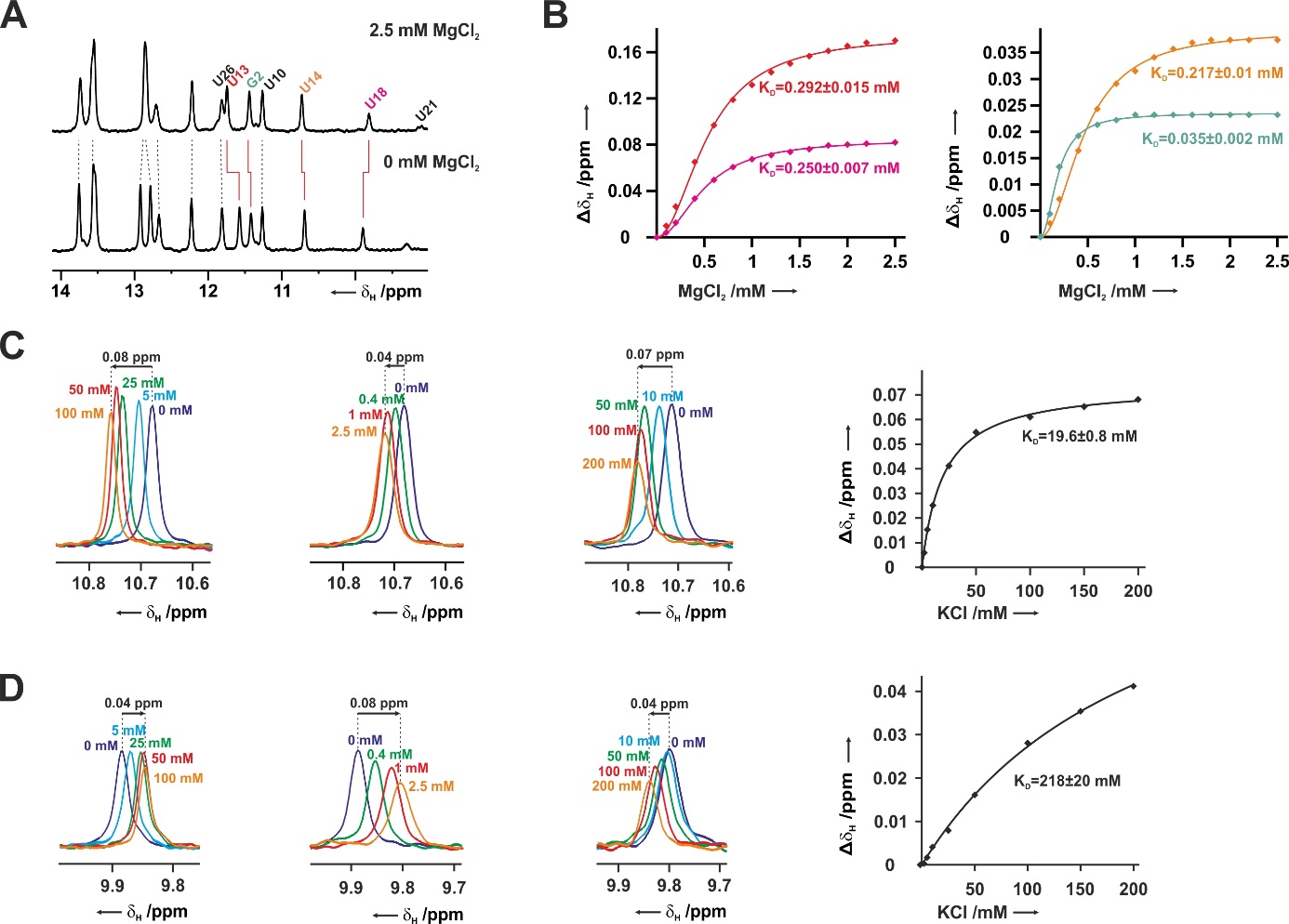


Figure S7**.** Mg^2+^ binding to the wild-type neomycin riboswitch ribostamycin complex. (A) comparison of 1D-^1^H imino proton spectra of the NSR/RIO complex in 50 mM BisTris buffer, pH 6.3, at 10° C, in the absence (bottom) and the presence (top) of saturating amounts (2.5 mM) of MgCl_2_ and in the absence of KCl. Imino proton assignments for the G/U wobble base pair G2/U26 and nucleotides from the U-turn are given and color coded. (B) Chemical shift changes as a function of Mg^2+^-concentrations for U13 and U18 (left) and U14 and G2 (right), respectively, using the same color code as in (A). (C) Chemical shift changes for the imino proton of U14 upon addition of KCl in the absence of MgCl_2_ (left), upon addition of MgCl_2_ in the absence of KCl (middle) and upon the addition of KCl in the presence of 2.5 mM MgCl_2_ (right). The corresponding binding isotherm for KCl in the presence of 2.5 mM MgCl_2_ is shown on the far right. (D) Chemical shift changes for the imino proton of U18 upon addition of KCl in the absence of MgCl_2_ (left), upon addition of MgCl_2_ in the absence of KCl (middle) and upon the addition of KCl in the presence of 2.5 mM MgCl_2_ (right). The corresponding binding isotherm for KCl in the presence of 2.5 mM MgCl_2_ is shown on the far right.


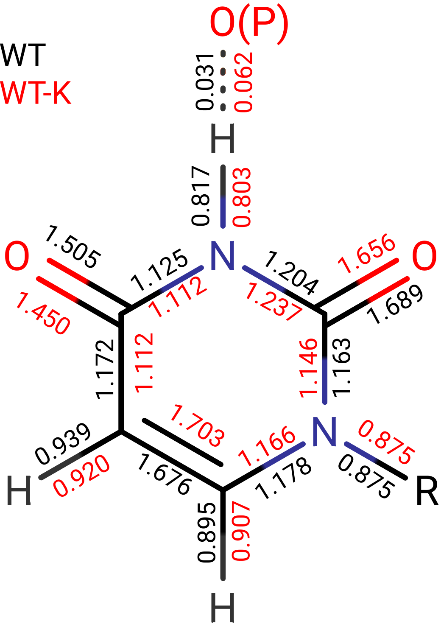


Figure S8. Wiberg bond orders  ([35](#_ENREF_35)) for U14 of the 2n0j_wt (black) and 2n0j_wt_K^+^ (red) QM/MM structures. The bond orders (BO) were computed from the QM/MM densities using Turbomole. BOs are a conceptual tool to rationalize differences in chemical bonding from a Lewis-structure perspective. However, their reliability is limited and as such, differences below 0.1 should only be analyzed with care. The reported BOs for the two U14 bases are very similar to each other with the difference often significantly below 0.1. However, the differences are systematic, and most BOs for the WT-K variant are slightly smaller than their WT counterparts. This suggests that a weak nucleobase polarization by K^+^ ion may exist but it is not clearly visible in the computed BOs.


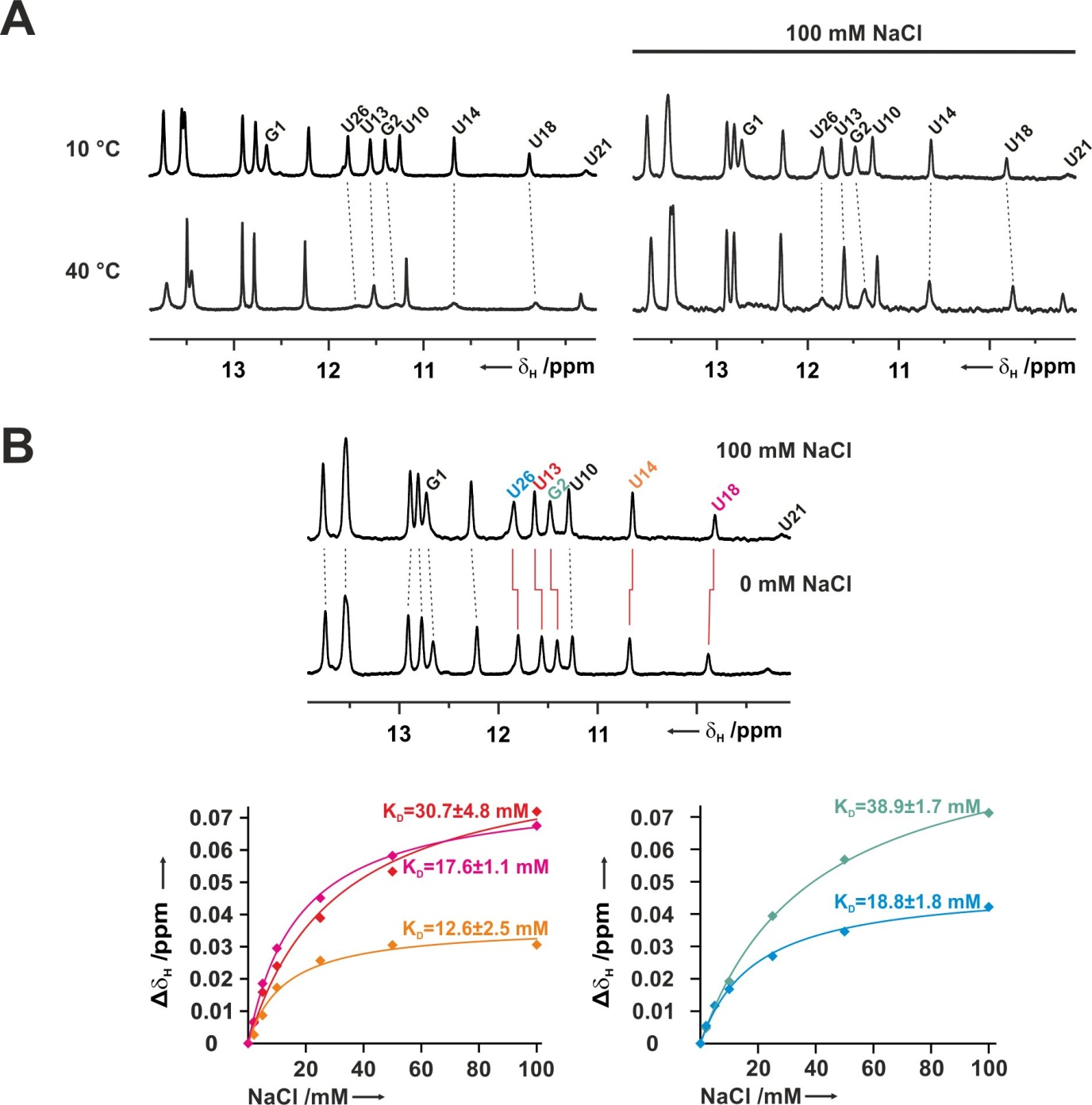


Figure S9. The effects of sodium ion binding on the wild-type NSR/RIO complex. (A) The presence of sodium ions stabilizes the complex. Comparison of the 1D-^1^H imino proton spectra at 10°C (top) and 40°C (bottom) in the absence (left) and the presence of 100 mM NaCl. In the presence of 100 mM NaCl the imino proton resonances of the U-turn U13, U14 and U18 become much less broadened upon increasing the temperature than in the absence of NaCl. (B) Effects of NaCl addition on the imino proton spectra of the wild-type NSR/RIO complex. The presence of sodium ions induces chemical shift changes for U13, U14 and U18 as well as for G2 and U26 of the G/U wobble base pair in the stem of the hairpin loop (top). Chemical shift changes as a function of NaCl concentration (bottom) reveal K_D_ values for sodium ion binding at the U-turn (U13, U14 and U18) similar to those for potassium ions. The color code for the individual binding curves corresponds to the color code used for indicating the signal assignments in the imino proton spectra (top).


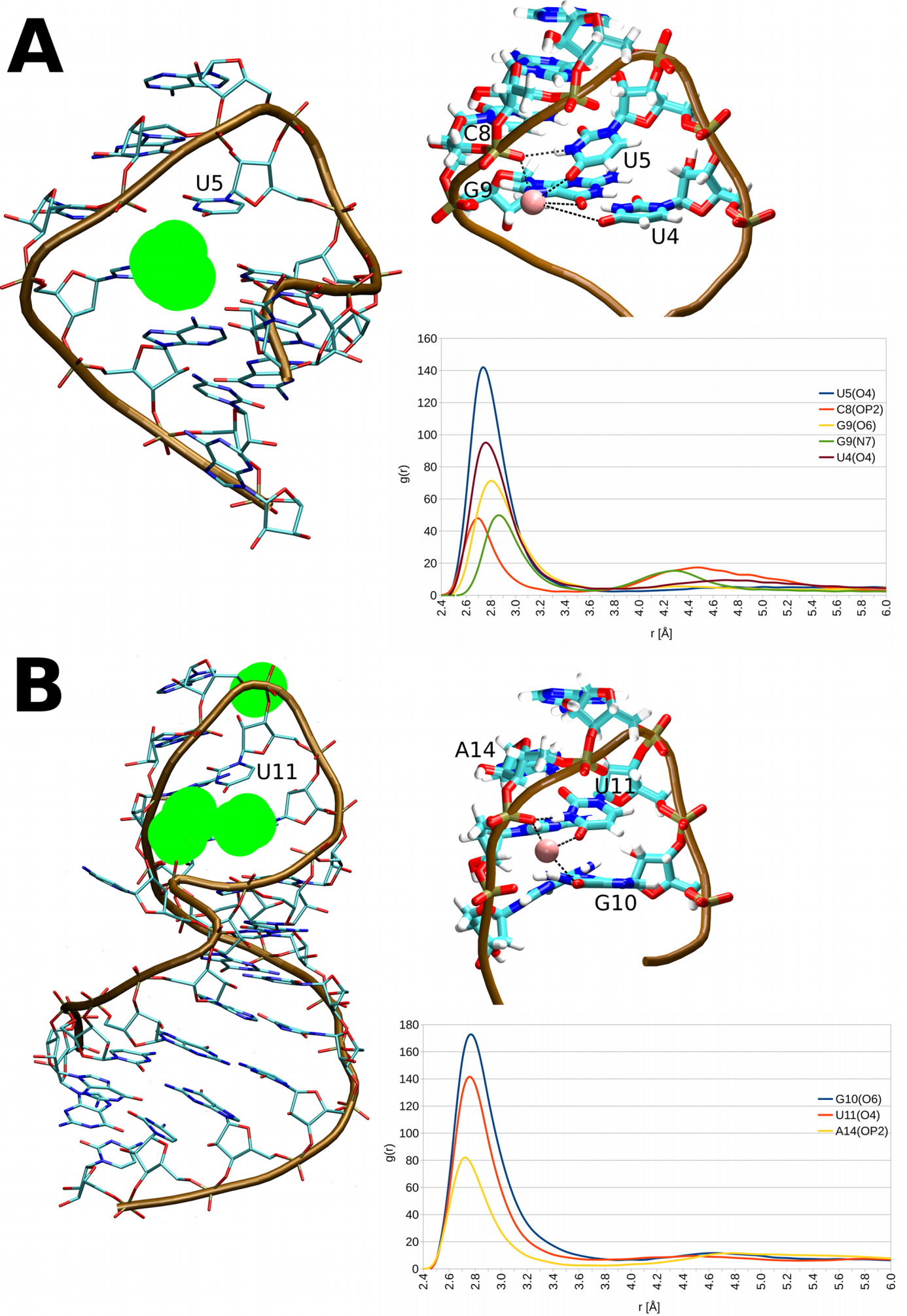


Figure S10. Analysis of the simulation ion binding site near the conserved uracil base in U-turn loops of the HIV-1 RNA A-Rich Hairpin Loop (**A**) (PDB: 1bvj) and a fragment (residues 47 to 47K) of the Mouse tRNA(Sec) (**B**) (PDB: 3rg5) The green spheres represent the highest density regions for the K^+^ ions in MD simulations. The detail of the key ion binding site in both systems is shown with the K^+^ ion in pink. The coordinating RNA residues are labeled. The ion coordination and the U-turn signature H-bond are indicated by black dashed lines. The graphs show normalized radial distribution functions of K^+^ ions pair-wise distances for the individual coordinating atoms in both systems.

**
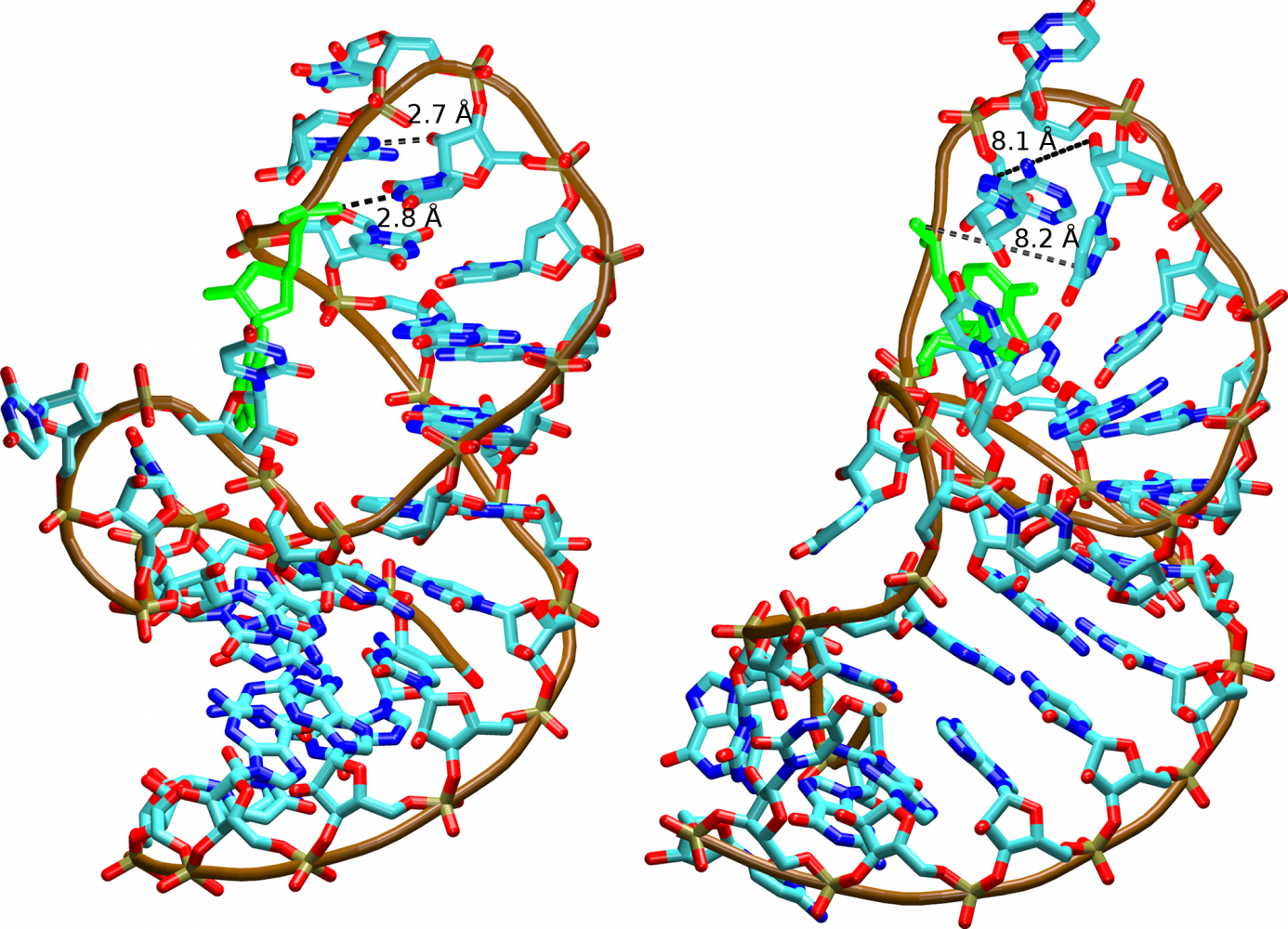
**

Figure S11. Left – initial structure of the wild-type NSR. Right – Permanently disrupted wild-type NSR structure without the RIO ligand, as observed at the end of a 10-μs-long simulation. The dashed black lines indicate signature H-bonds of the U-turn motif with their interatomic distances labeled. The A17 nucleotide is in green.


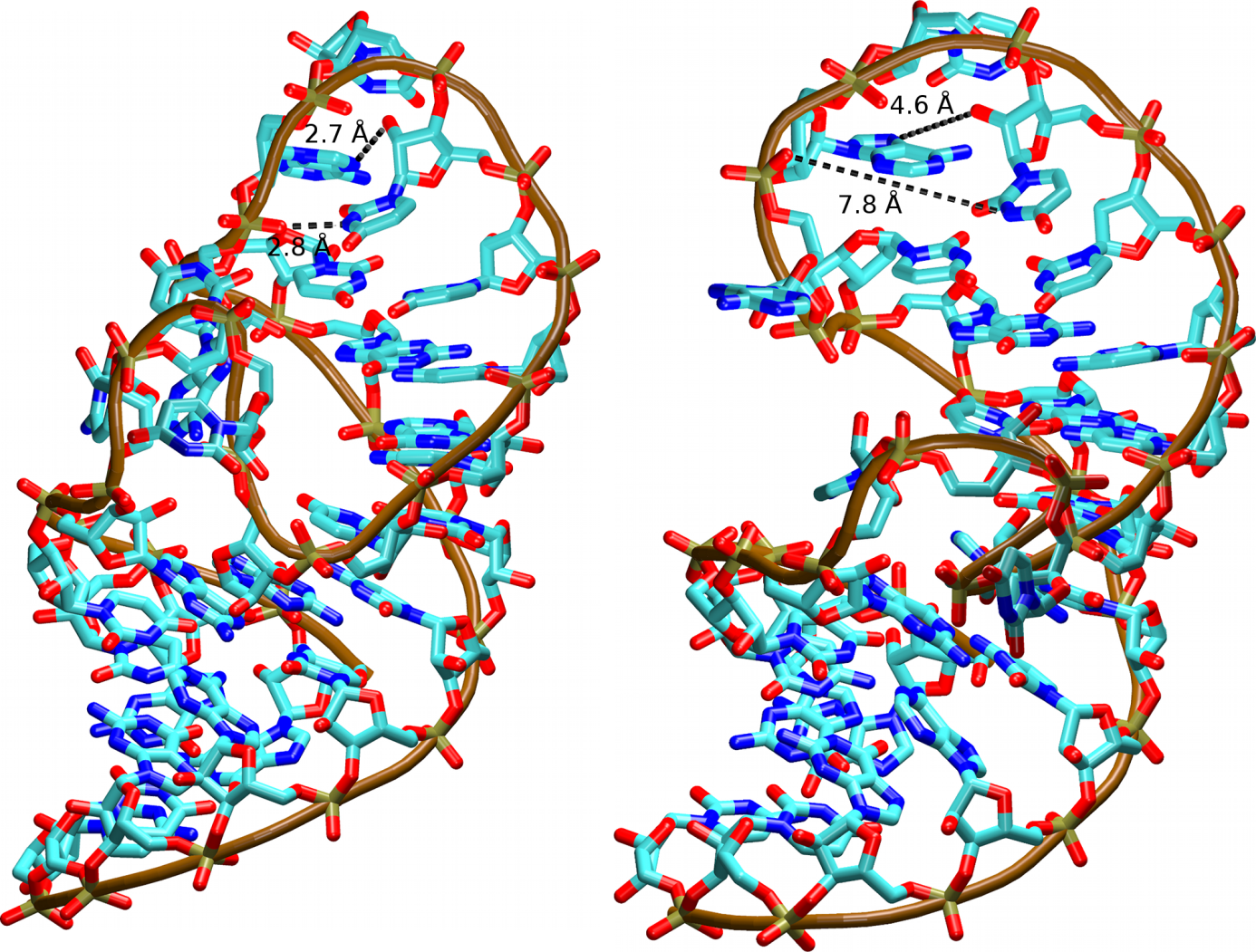


Figure S12. Left – initial structure of the wild-type NSR. Right – example of a temporary, reversible structural distortion of the U-turn loop observed in the MD simulations. The dashed black lines indicate signature H-bonds of the U-turn motif with their interatomic distances labeled.


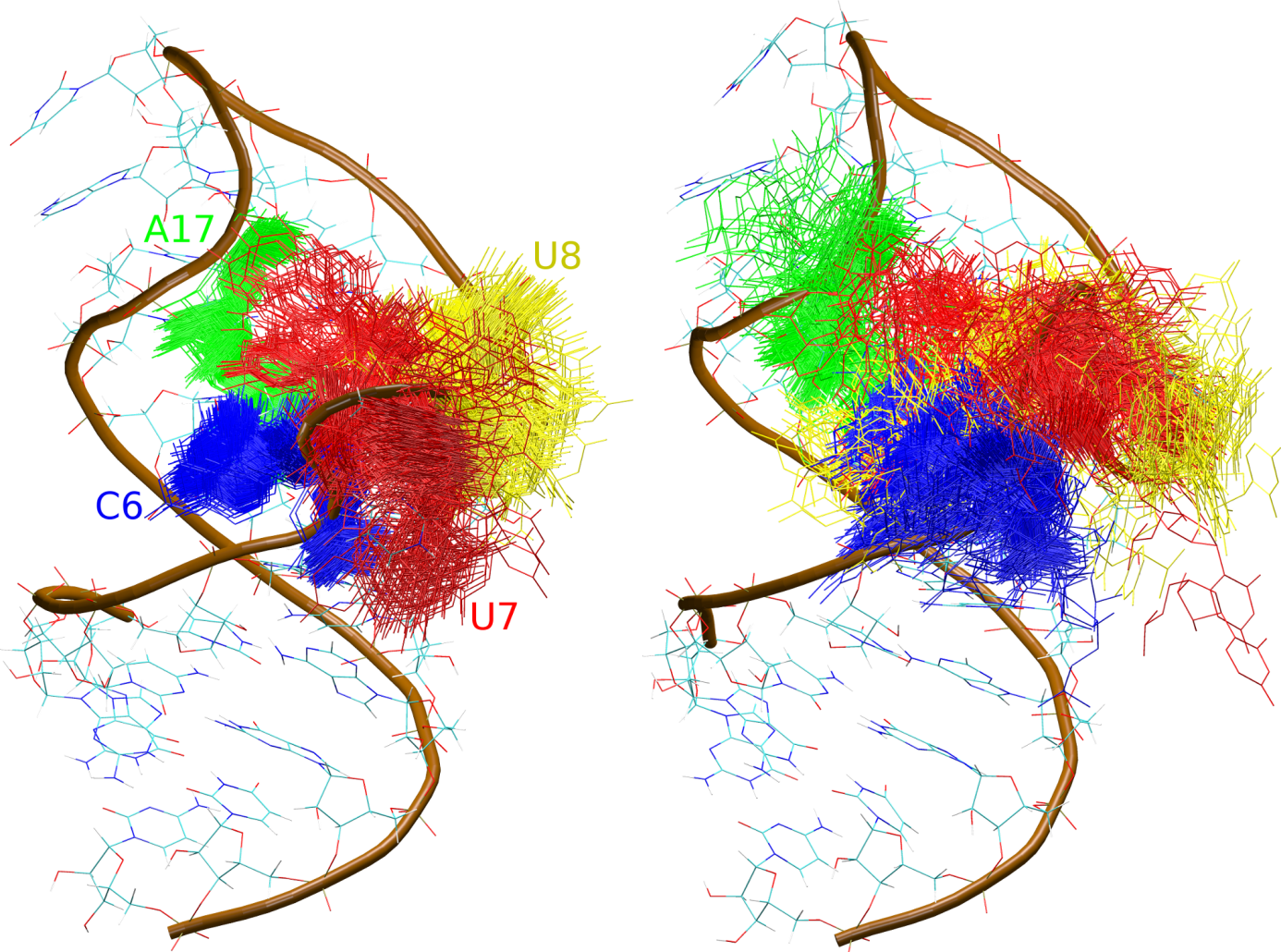


Figure S13. An overlay image of the C6 (blue), U7 (red), U8 (yellow), and A17 (green) nucleotides in frames of the MD trajectories of the wild-type NSR system, with (left) and without (right) the RIO ligand. An qualitatively identical result was obtained also for the C14+ mutant systems.


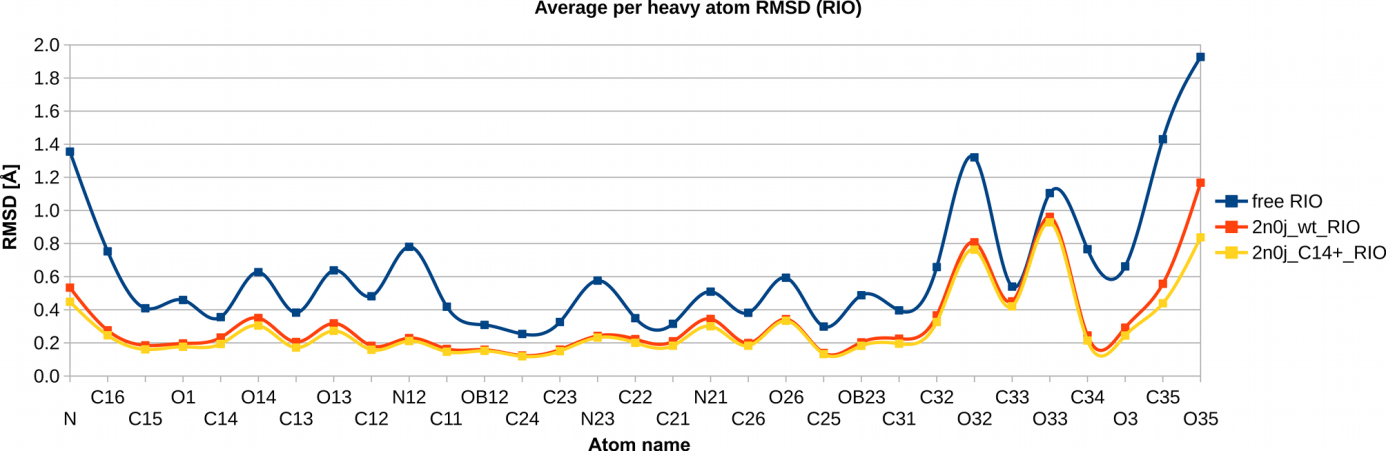


Figure S14. The graph of the average per heavy atom RMSD of the ribostamycin (RIO) ligand in the MD simulations of the individual systems. An averaged structure of each MD trajectory ensemble was used as the reference structure.


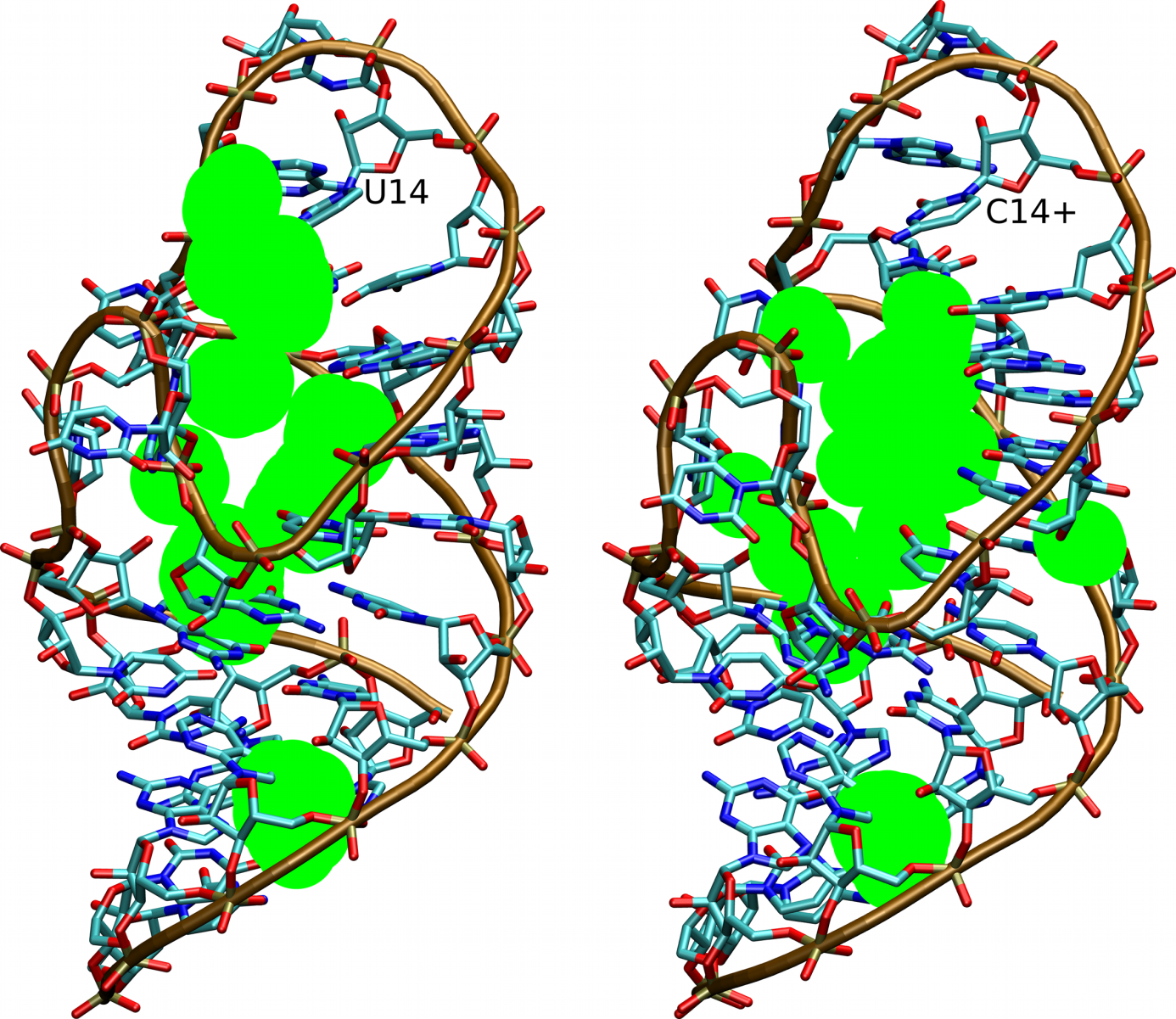


Figure S15. K^+^ binding to NSR in MD simulations in the absence of ligand. The green spheres represent the highest density points for the K^+^ ions in simulations of the wild-type NSR system (left) and the C14+ mutant (right) without RIO.


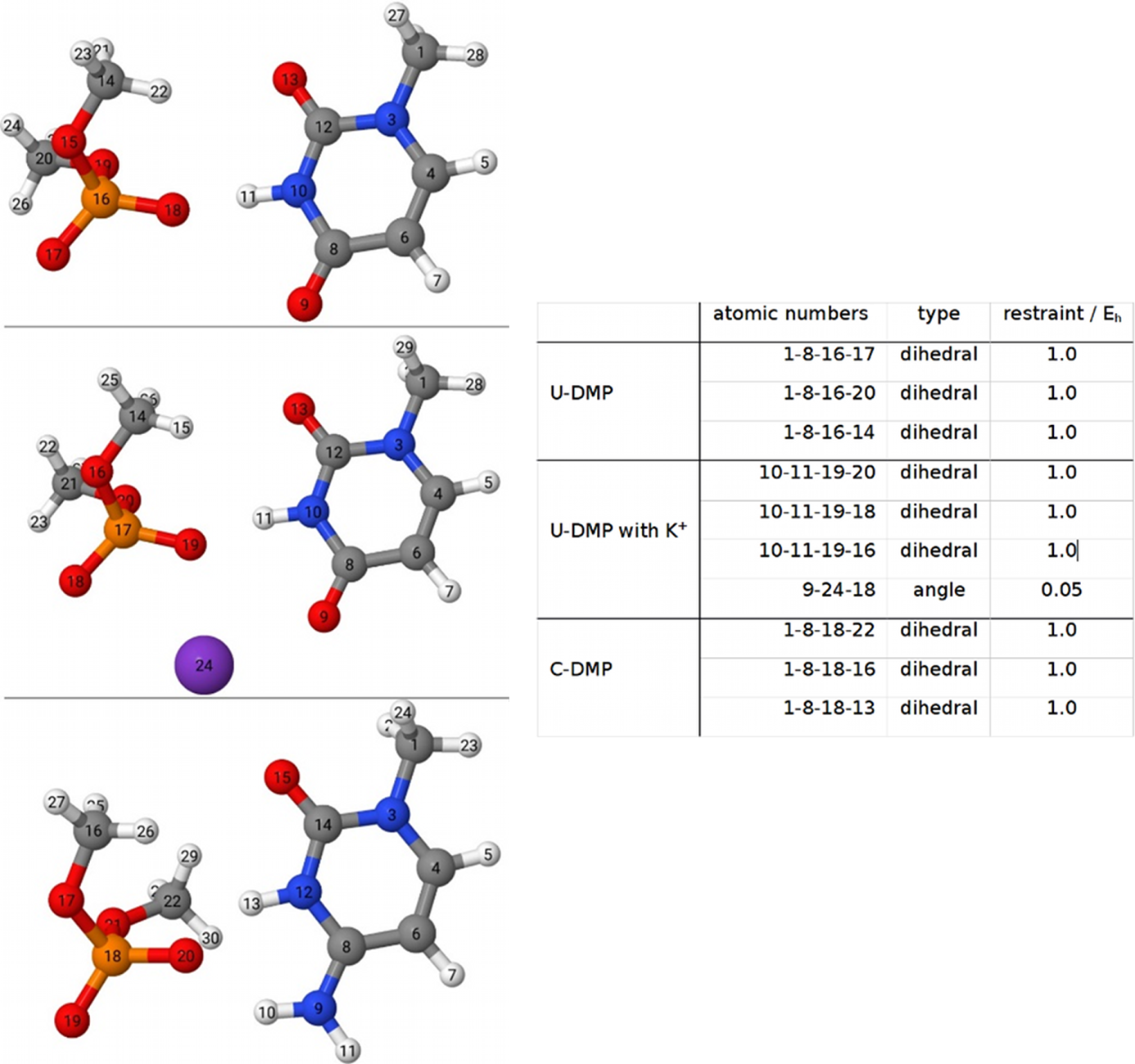


Figure S16. The model systems used in some of the QM calculations to study the U14(N3)/A17(OP2) (top and middle) and C14+(N3)/A17(OP2) interactions (bottom). The K^+^ ion is shown in purple. The atom numbers are labeled. The table lists restraints which were applied to the optimizations of the model systems as depicted in the figure.


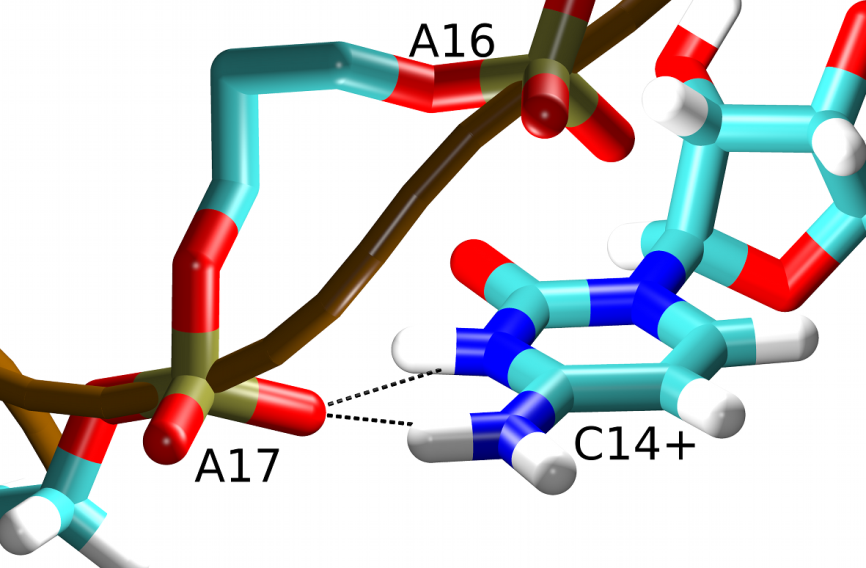


Figure S17. The C14+(N4,N3)/A17(OP2) bifurcated H-bond was often seen in the MD simulations. However, it was unstable in both the QM/MM and MM optimizations where the acceptor moved closer to the N3 atom. The dashed black lines indicate H-bonds.


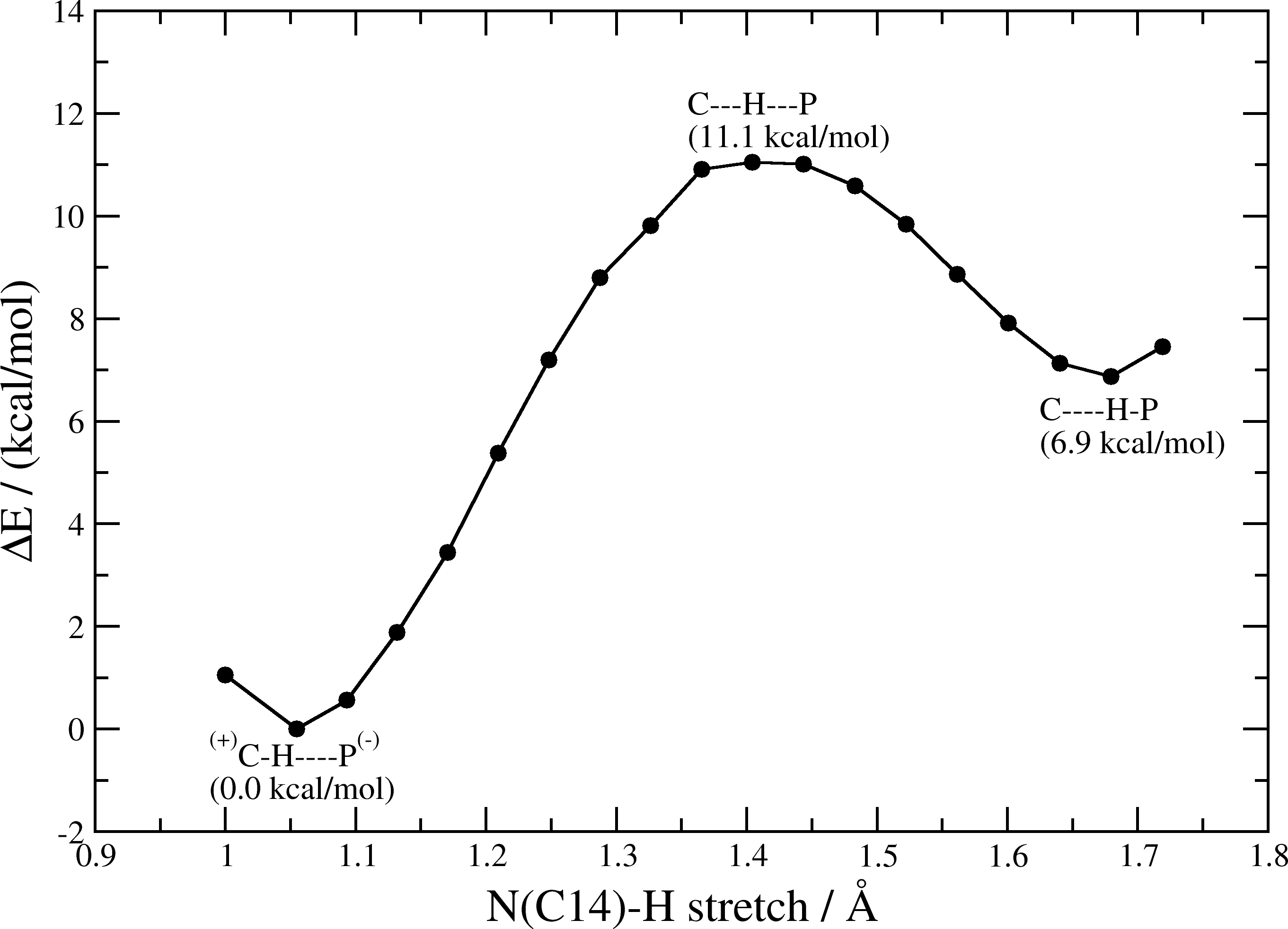


Figure S18. Relaxed potential energy surface scan of the proton transfer between C14+(N3) and A17(OP2) using DLPNO-CCSD(T)/def2-TZVP single point energies at B3LYP-D3/def2-TZVP structures (applying a continuum solvation model at both computational levels).


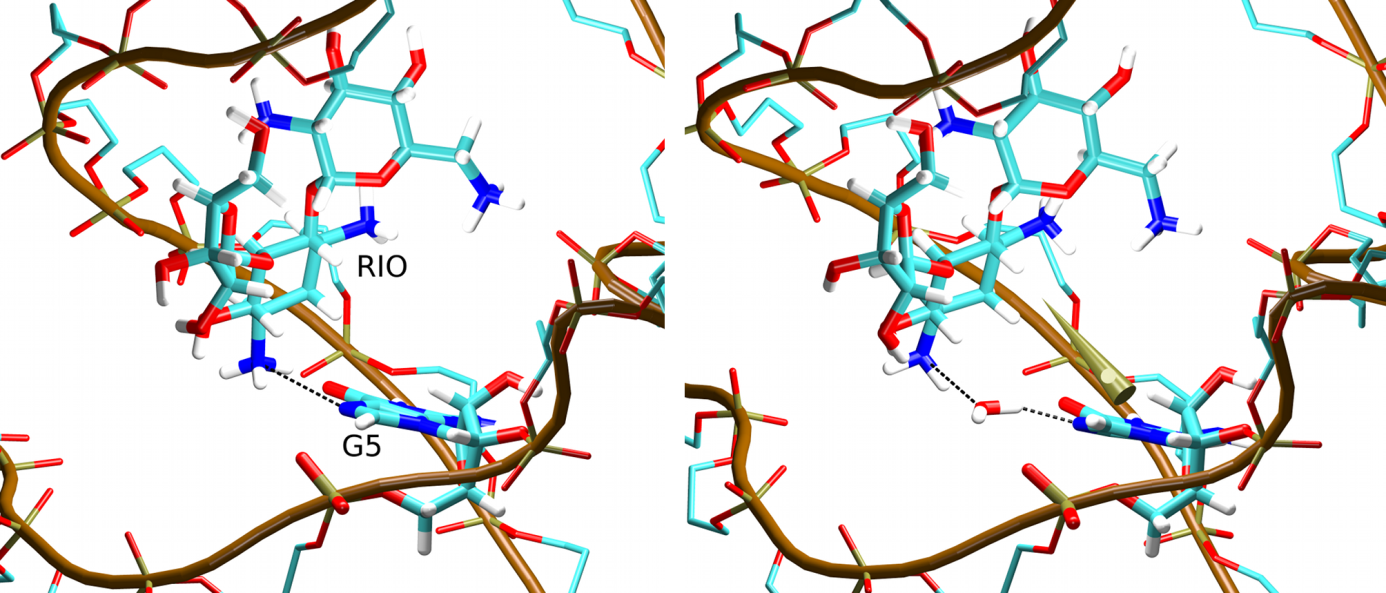


Figure S19. An example for a temporary transition from a direct (left) to a water-mediated (right) H-bond arrangement of one of the RNA/RIO H-bond interactions (see Table S2) in simulations of the NSR system.


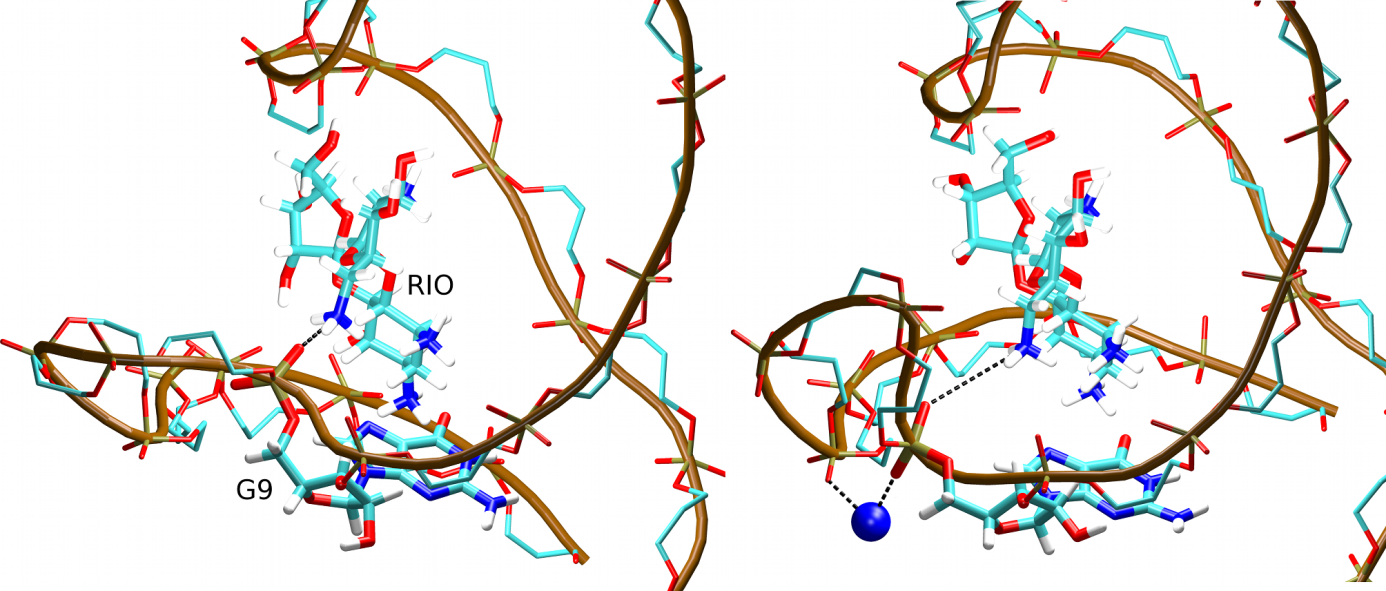


Figure S20. A sodium specific ion site often formed in the simulations of NSR containing sodium ions (right). This sometimes disturbed the native G9(OP1)/RIO(N) H-bond (left) by pulling the phosphate away from the ligand (see Table S2). The H-bond and the ion coordination are indicated by black dashed lines. This has been the only noticeable difference between simulations with K^+^ and Na^+^, as this ion binding site was absent in presence of K^+^.

# Supplementary Information Datasets

**Ribostamycin (RIO) parameters in mol2 format:**

@<TRIPOS>MOLECULE

generated by VMD

69 71 1 0 0

SMALL

USER_CHARGES

****

Energy = 0

@<TRIPOS>ATOM

1 N 3.9680 2.2680 1.8980 n4 101 RIO -0.062000

2 C16 3.7060 3.7000 1.8760 c3 101 RIO -0.161000

3 H161 4.1740 4.1340 0.9900 h1 101 RIO 0.175500

4 H162 4.1350 4.1630 2.7670 h1 101 RIO 0.175500

5 C15 2.2020 4.0010 1.8310 c3 101 RIO 0.005000

6 H15 1.7900 3.5570 0.9260 h1 101 RIO 0.151000

7 O1 1.5790 3.4130 2.9710 os 101 RIO -0.300000

8 C14 1.9750 5.5240 1.8070 c3 101 RIO -0.001000

9 H14 2.4410 5.9700 2.6880 h1 101 RIO 0.155000

10 O14 2.5520 6.0760 0.6370 oh 101 RIO -0.374000

11 HO14 2.3850 7.0200 0.6420 ho 101 RIO 0.266000

12 C13 0.4660 5.7980 1.8240 c3 101 RIO 0.000000

13 H13 0.0380 5.4220 0.9010 h1 101 RIO 0.136000

14 O13 0.2250 7.1910 1.8910 oh 101 RIO -0.328000

15 HO13 -0.1640 7.4650 1.0550 ho 101 RIO 0.271000

16 C12 -0.2010 5.0850 3.0080 c3 101 RIO -0.099000

17 H12 0.1410 5.5460 3.9280 hx 101 RIO 0.182000

18 N12 -1.6600 5.2500 2.9400 n4 101 RIO -0.059000

19 H121 -2.0860 4.8260 3.7500 hn 101 RIO 0.285000

20 H122 -2.0110 4.8040 2.1070 hn 101 RIO 0.285000

21 H123 -1.8910 6.2320 2.9160 hn 101 RIO 0.285000

22 C11 0.1710 3.6000 3.0860 c3 101 RIO 0.153000

23 H11 -0.1520 3.2590 4.0690 h2 101 RIO 0.184000

24 OB12 -0.5170 2.8740 2.0730 os 101 RIO -0.309000

25 C24 -0.5980 1.4490 2.2290 c3 101 RIO 0.016000

26 H24 0.2780 1.0670 2.7660 h1 101 RIO 0.138000

27 C23 -0.5710 0.8750 0.7960 c3 101 RIO -0.074000

28 H23 -1.4310 1.2520 0.2390 hx 101 RIO 0.179000

29 N23 0.6420 1.2720 0.0730 n4 101 RIO -0.069000

30 H231 0.6890 2.2760 0.0180 hn 101 RIO 0.279333

31 H232 0.6270 0.8870 -0.8580 hn 101 RIO 0.279333

32 H233 1.4550 0.9310 0.5590 hn 101 RIO 0.279333

33 C22 -0.6350 -0.6470 0.8330 c3 101 RIO -0.214000

34 H221 0.2230 -1.0360 1.3770 hc 101 RIO 0.145000

35 H222 -0.6230 -1.0390 -0.1830 hc 101 RIO 0.145000

36 C21 -1.9160 -1.0900 1.5300 c3 101 RIO -0.082000

37 H21 -2.7690 -0.7530 0.9410 hx 101 RIO 0.180000

38 N21 -1.9530 -2.5590 1.5990 n4 101 RIO -0.062000

39 H211 -1.9100 -2.9440 0.6650 hn 101 RIO 0.284333

40 H212 -2.8080 -2.8560 2.0490 hn 101 RIO 0.284333

41 H213 -1.1630 -2.8920 2.1330 hn 101 RIO 0.284333

42 C26 -1.9830 -0.4700 2.9340 c3 101 RIO -0.001000

43 H26 -1.1800 -0.8960 3.5400 h1 101 RIO 0.127000

44 O26 -3.2270 -0.8470 3.5030 oh 101 RIO -0.340000

45 HO26 -3.2680 -1.8040 3.5170 ho 101 RIO 0.293000

46 C25 -1.8490 1.0610 2.9500 c3 101 RIO -0.003000

47 H25 -2.7080 1.5330 2.4700 h1 101 RIO 0.133000

48 OB23 -1.6980 1.4670 4.3010 os 101 RIO -0.310000

49 C31 -2.8720 1.8820 4.9890 c3 101 RIO 0.103000

50 H31 -3.7630 1.3980 4.5830 h2 101 RIO 0.153000

51 C32 -2.7370 1.5490 6.4820 c3 101 RIO 0.002000

52 H32 -1.8770 0.9060 6.6530 h1 101 RIO 0.136000

53 O32 -3.9110 0.9030 6.9380 oh 101 RIO -0.336000

54 HO32 -4.6590 1.4800 6.7660 ho 101 RIO 0.272000

55 C33 -2.5190 2.9210 7.1180 c3 101 RIO -0.008000

56 H33 -1.4600 3.1620 7.0680 h1 101 RIO 0.128000

57 O33 -2.9450 2.9980 8.4640 oh 101 RIO -0.313000

58 HO33 -2.4380 2.3720 8.9860 ho 101 RIO 0.262000

59 C34 -3.2950 3.8350 6.1750 c3 101 RIO -0.022000

60 H34 -4.3650 3.7450 6.3670 h1 101 RIO 0.171000

61 O3 -3.0140 3.2960 4.8930 os 101 RIO -0.349000

62 C35 -2.8400 5.2960 6.2920 c3 101 RIO -0.039000

63 H351 -2.5170 5.6410 5.3110 h1 101 RIO 0.130500

64 H352 -2.0030 5.3620 6.9890 h1 101 RIO 0.130500

65 O35 -3.8880 6.1290 6.7480 oh 101 RIO -0.411000

66 HO35 -4.5760 6.1470 6.0790 ho 101 RIO 0.252000

67 HAW 3.5670 1.8360 1.0650 hn 1 RIO 0.276333

68 HBW 3.5450 1.8580 2.7310 hn 1 RIO 0.276333

69 HCW 4.8760 1.6660 1.9490 hn 1 RIO 0.276333

@<TRIPOS>BOND

1 1 2 1

2 1 68 1

3 1 67 1

4 1 69 1

5 2 3 1

6 2 4 1

7 2 5 1

8 5 6 1

9 5 7 1

10 5 8 1

11 7 22 1

12 8 9 1

13 8 10 1

14 8 12 1

15 10 11 1

16 12 13 1

17 12 14 1

18 12 16 1

19 14 15 1

20 16 17 1

21 16 18 1

22 16 22 1

23 18 19 1

24 18 20 1

25 18 21 1

26 22 23 1

27 22 24 1

28 24 25 1

29 25 26 1

30 25 27 1

31 25 46 1

32 27 28 1

33 27 29 1

34 27 33 1

35 29 30 1

36 29 31 1

37 29 32 1

38 33 34 1

39 33 35 1

40 33 36 1

41 36 37 1

42 36 38 1

43 36 42 1

44 38 39 1

45 38 40 1

46 38 41 1

47 42 43 1

48 42 44 1

49 42 46 1

50 44 45 1

51 46 47 1

52 46 48 1

53 48 49 1

54 49 50 1

55 49 51 1

56 49 61 1

57 51 52 1

58 51 53 1

59 51 55 1

60 53 54 1

61 55 56 1

62 55 57 1

63 55 59 1

64 57 58 1

65 59 60 1

66 59 61 1

67 59 62 1

68 62 63 1

69 62 64 1

70 62 65 1

71 65 66 1

@<TRIPOS>SUBSTRUCTURE

1 **** 1 TEMP 0 **** **** 0 ROOT

**N3 protonated cytosine (CP) parameters in mol2 format:**

@<TRIPOS>MOLECULE

CP

32 33 1 0 1

SMALL

USER_CHARGES

@<TRIPOS>ATOM

1 P 2.314489 1.776673 -0.478436 P 1 CP 1.1662 ****

2 OP1 1.933410 3.073374 -1.081549 O2 1 CP -0.7760 ****

3 OP2 3.156547 0.871841 -1.292450 O2 1 CP -0.7760 ****

4 O5' 2.994369 2.142475 0.922974 OS 1 CP -0.4989 ****

5 C5' 2.156035 2.551837 2.019887 CI 1 CP 0.0558 ****

6 H5' 1.452687 1.753841 2.257803 H1 1 CP 0.0679 ****

7 H5'' 1.604968 3.450028 1.741178 H1 1 CP 0.0679 ****

8 C4' 3.008252 2.847921 3.243186 CT 1 CP 0.1065 ****

9 H4' 2.447118 3.477373 3.933851 H1 1 CP 0.1174 ****

10 O4' 3.228121 1.605168 3.977231 OS 1 CP -0.3548 ****

11 C1' 4.558614 1.150950 3.777548 CT 1 CP 0.0935 ****

12 H1' 5.072002 1.095281 4.737461 H2 1 CP 0.2029 ****

13 N1 4.485689 -0.234531 3.276984 N* 1 CP 0.0638 ****

14 C6 4.434822 -0.525287 1.944988 C4 1 CP -0.0501 ****

15 H6 4.450328 0.280846 1.226944 H4 1 CP 0.2390 ****

16 C5 4.343212 -1.803676 1.520274 C4 1 CP -0.3510 ****

17 H5 4.291174 -2.014169 0.462316 HA 1 CP 0.2146 ****

18 C4 4.319830 -2.824428 2.526674 CA 1 CP 0.5581 ****

19 N4 4.226588 -4.113038 2.170429 N2 1 CP -0.8894 ****

20 H41 4.213907 -4.831230 2.880581 H 1 CP 0.4740 ****

21 H42 4.169275 -4.366118 1.194777 H 1 CP 0.4740 ****

22 N3 4.390522 -2.522967 3.828979 NA 1 CP -0.3181 ****

23 H3 4.378261 -3.256188 4.508751 H 1 CP 0.3406 ****

24 C2 4.475763 -1.230819 4.236789 C 1 CP 0.5454 ****

25 O2 4.540867 -0.935393 5.427913 O 1 CP -0.4814 ****

26 C3' 4.415606 3.377827 2.961306 CT 1 CP 0.2022 ****

27 H3' 4.412072 3.941489 2.028369 H1 1 CP 0.0615 ****

28 C2' 5.230443 2.095696 2.779425 CT 1 CP 0.0670 ****

29 H2' 5.668211 2.081313 1.781301 H1 1 CP 0.0972 ****

30 O2' 6.272982 2.049941 3.757136 OH 1 CP -0.6139 ****

31 HO2' 6.759278 1.235228 3.610987 HO 1 CP 0.4186 ****

32 O3' 5.029898 4.128649 3.998279 OS 1 CP -0.5246 ****

@<TRIPOS>BOND

1 1 2 1

2 1 3 1

3 1 4 1

4 4 5 1

5 5 6 1

6 5 7 1

7 5 8 1

8 8 9 1

9 8 10 1

10 8 26 1

11 10 11 1

12 11 12 1

13 11 13 1

14 11 28 1

15 13 14 1

16 13 24 1

17 14 15 1

18 14 16 1

19 16 17 1

20 16 18 1

21 18 19 1

22 18 22 1

23 19 20 1

24 19 21 1

25 22 23 1

26 22 24 1

27 24 25 1

28 26 27 1

29 26 28 1

30 26 32 1

31 28 29 1

32 28 30 1

33 30 31 1

@<TRIPOS>SUBSTRUCTURE

1 CP 1 **** 0 **** ****

**N3 protonated cytosine (CP) parameters in frcmod format (force-field modification for the bsc0χ_OL3_ RNA force field as implemented in AMBER16):**

# force field modification for CP

ANGLE

CM-CA-NA 70.0 120.50

C4-CA-NA 70.0 120.50
